# Supplementary figures and images for: Serine ADP-ribosylation marks nucleosomes for ALC1-dependent chromatin remodeling
Source: eLife. 2021 Dec 7;10:e71502. doi: 10.7554/eLife.71502 (PMC8683085; doi:10.7554/eLife.71502)

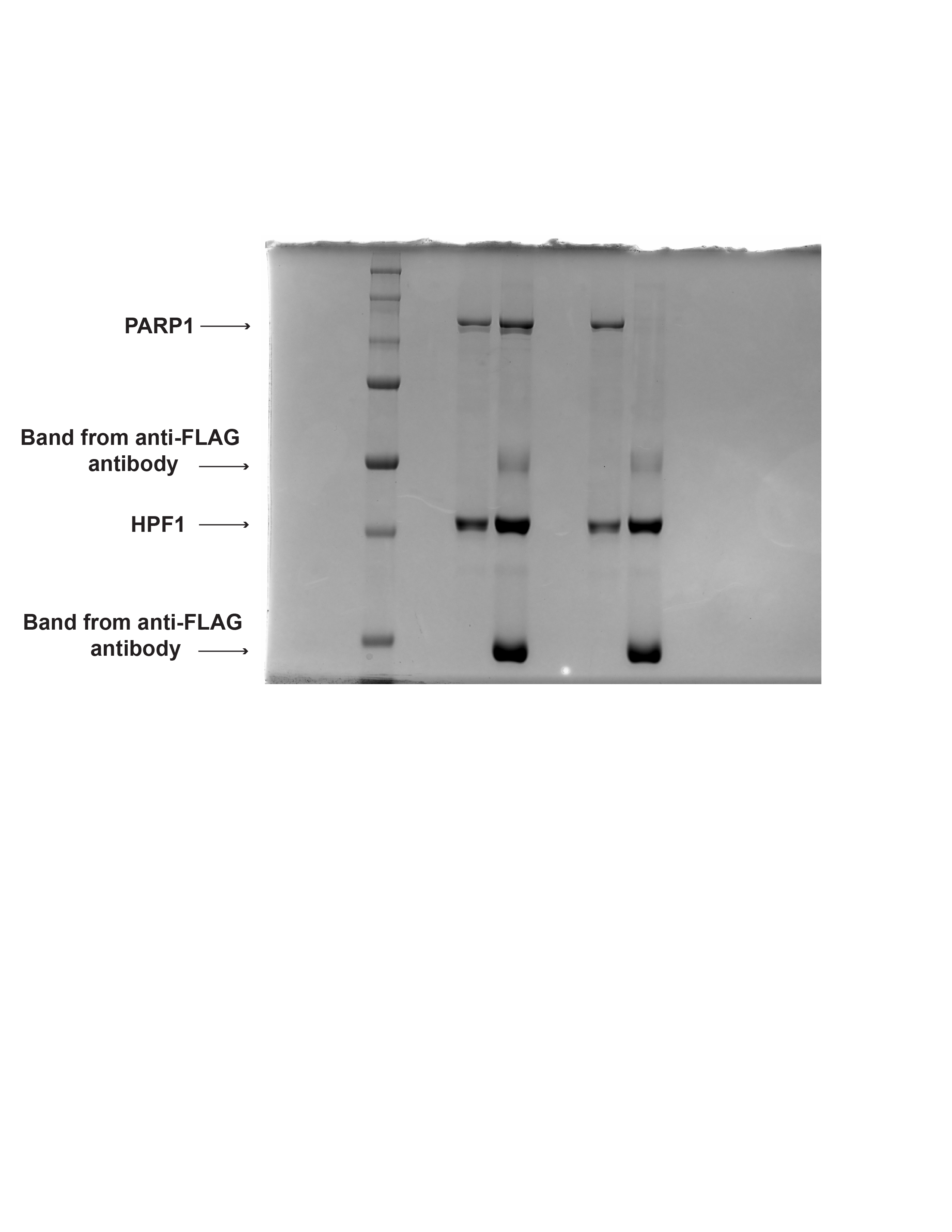

Supplement: Figure 2—figure supplement 1—source data 1. [file elife-71502-fig2-figsupp1-data1.zip › Figure 2-figure supplement 1D-source data 1.tif]

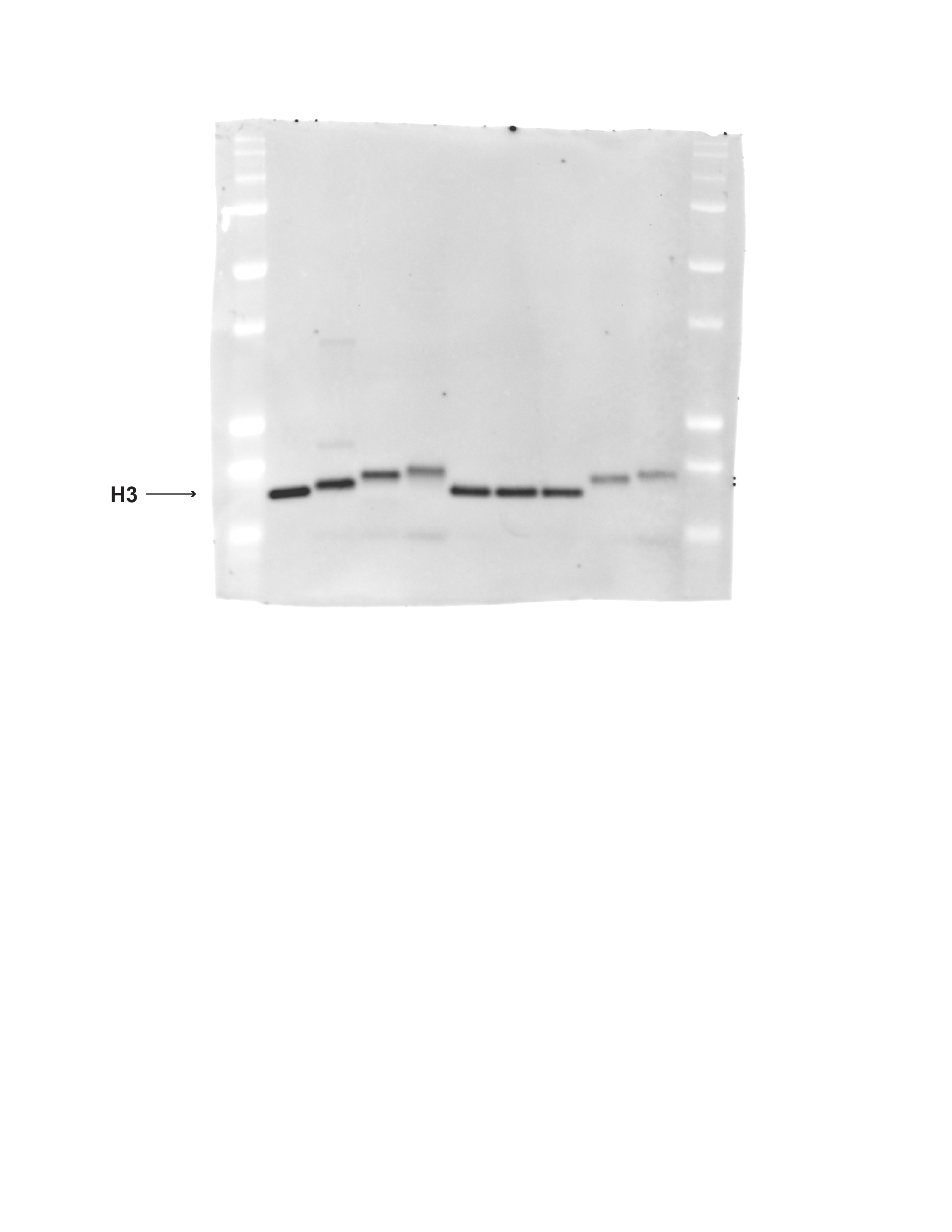

Supplement: Figure 4—source data 1. [file elife-71502-fig4-data1.zip › Figure 4-source data 1/Figure 4C-source data 1.tif]

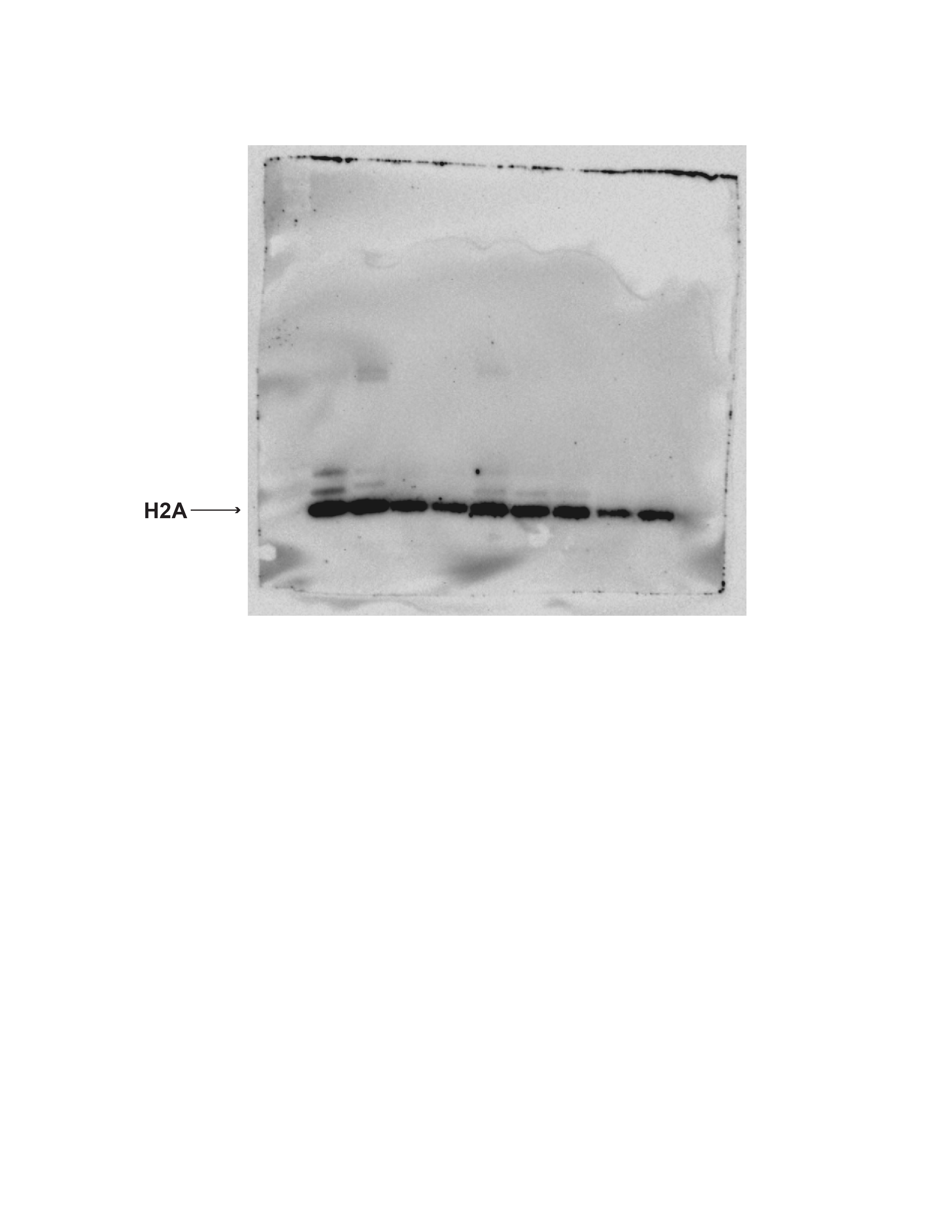

Supplement: Figure 4—source data 1. [file elife-71502-fig4-data1.zip › Figure 4-source data 1/Figure 4C-source data 2.tif]

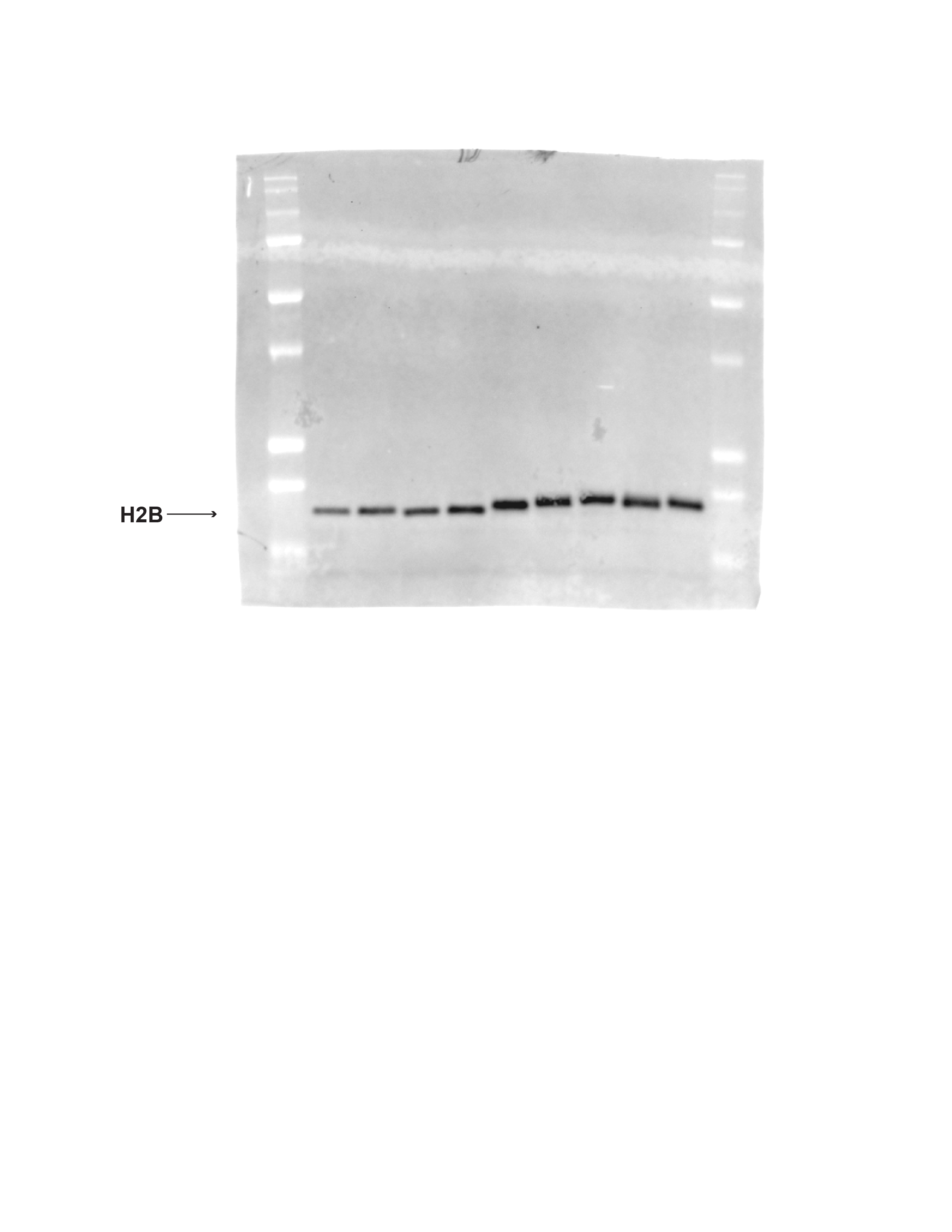

Supplement: Figure 4—source data 1. [file elife-71502-fig4-data1.zip › Figure 4-source data 1/Figure 4D-source data 1.tif]

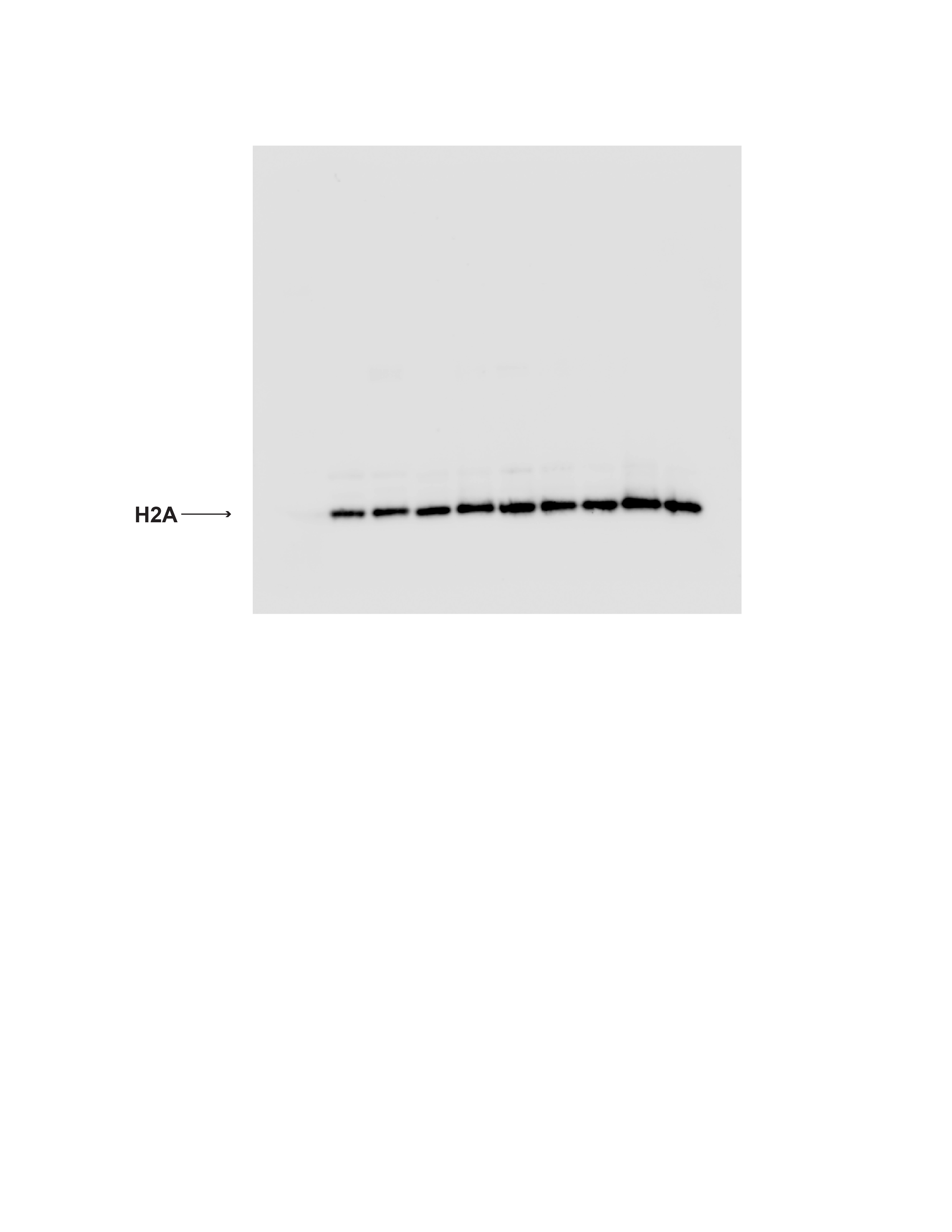

Supplement: Figure 4—source data 1. [file elife-71502-fig4-data1.zip › Figure 4-source data 1/Figure 4D-source data 2.tif]

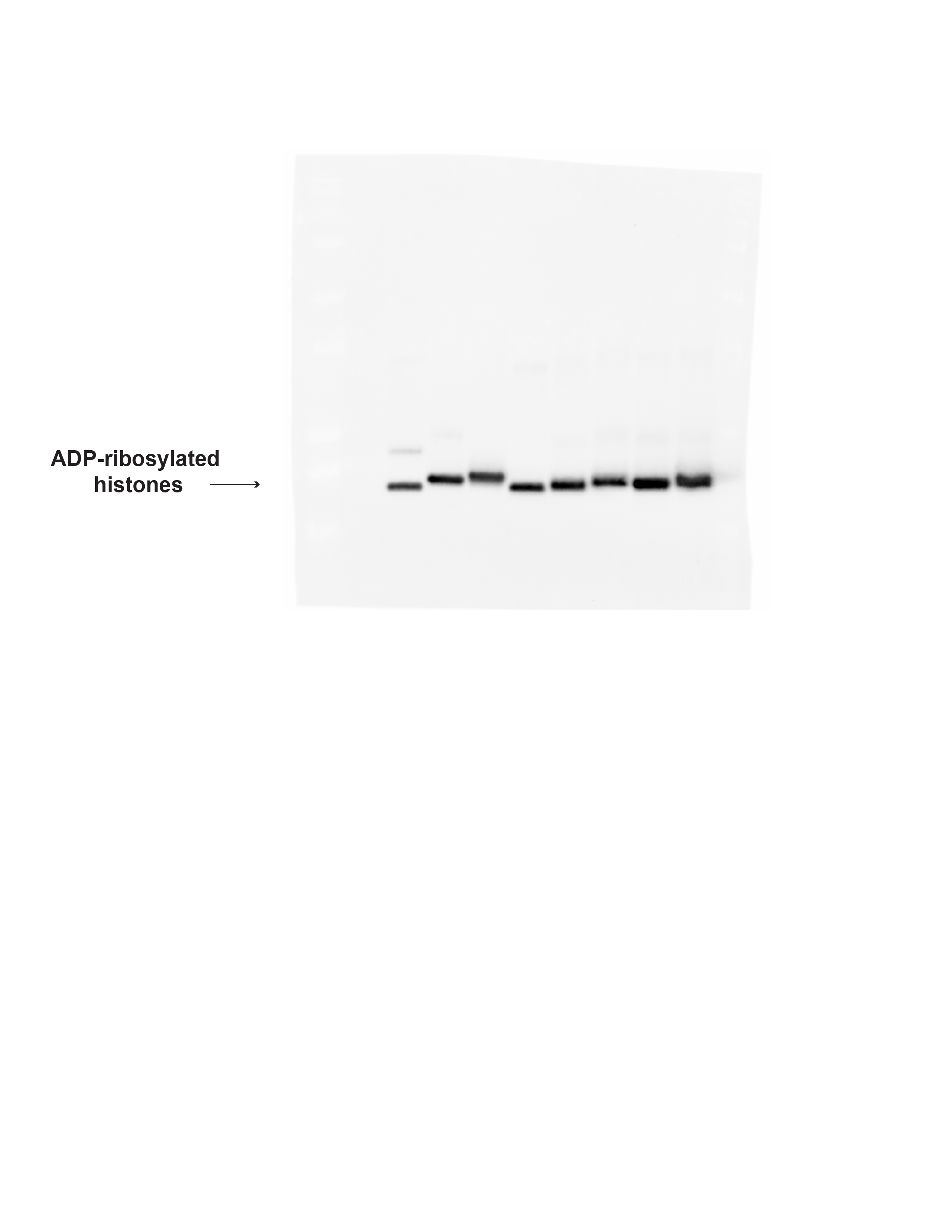

Supplement: Figure 4—source data 1. [file elife-71502-fig4-data1.zip › Figure 4-source data 1/Figure 4E-source data 1.tif]

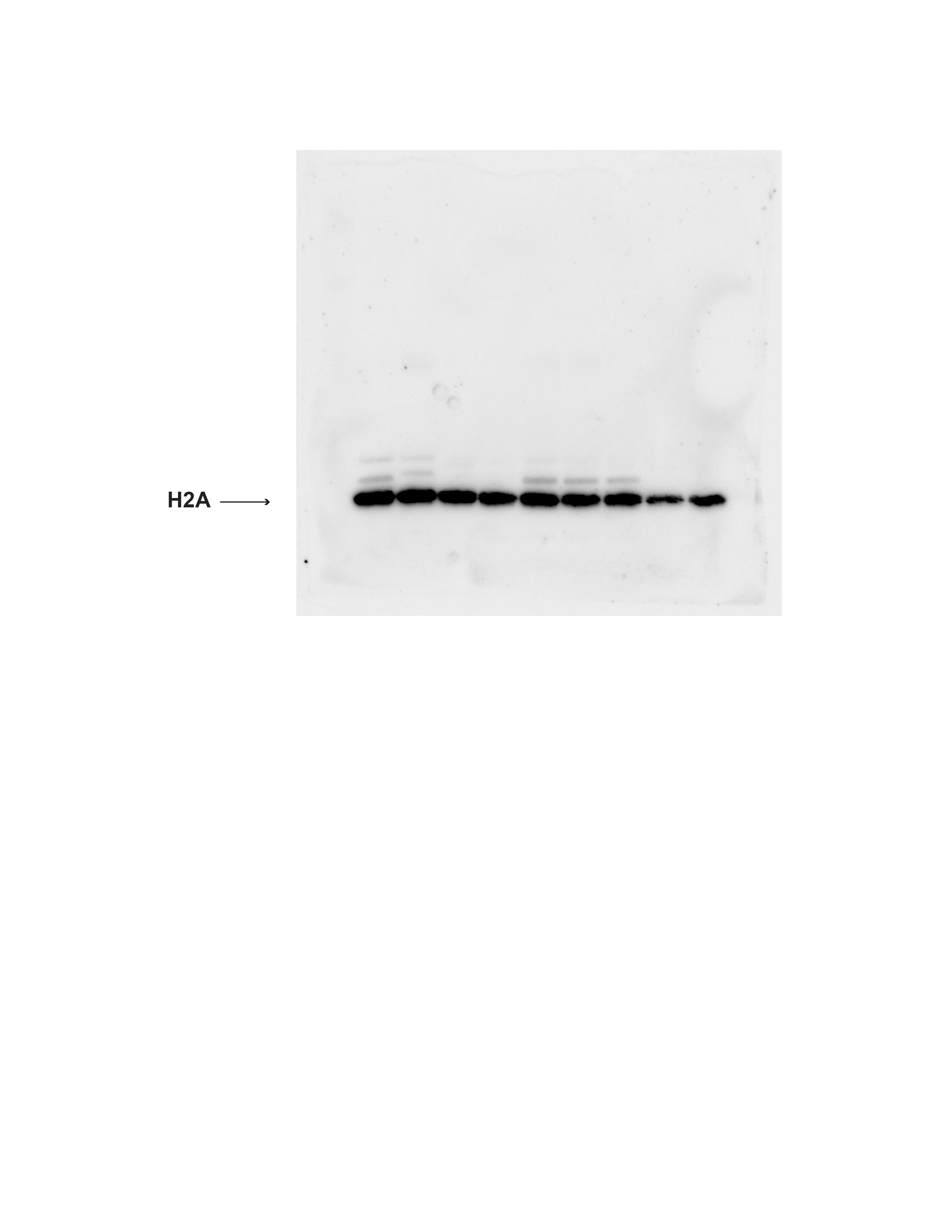

Supplement: Figure 4—source data 1. [file elife-71502-fig4-data1.zip › Figure 4-source data 1/Figure 4E-source data 2.tif]

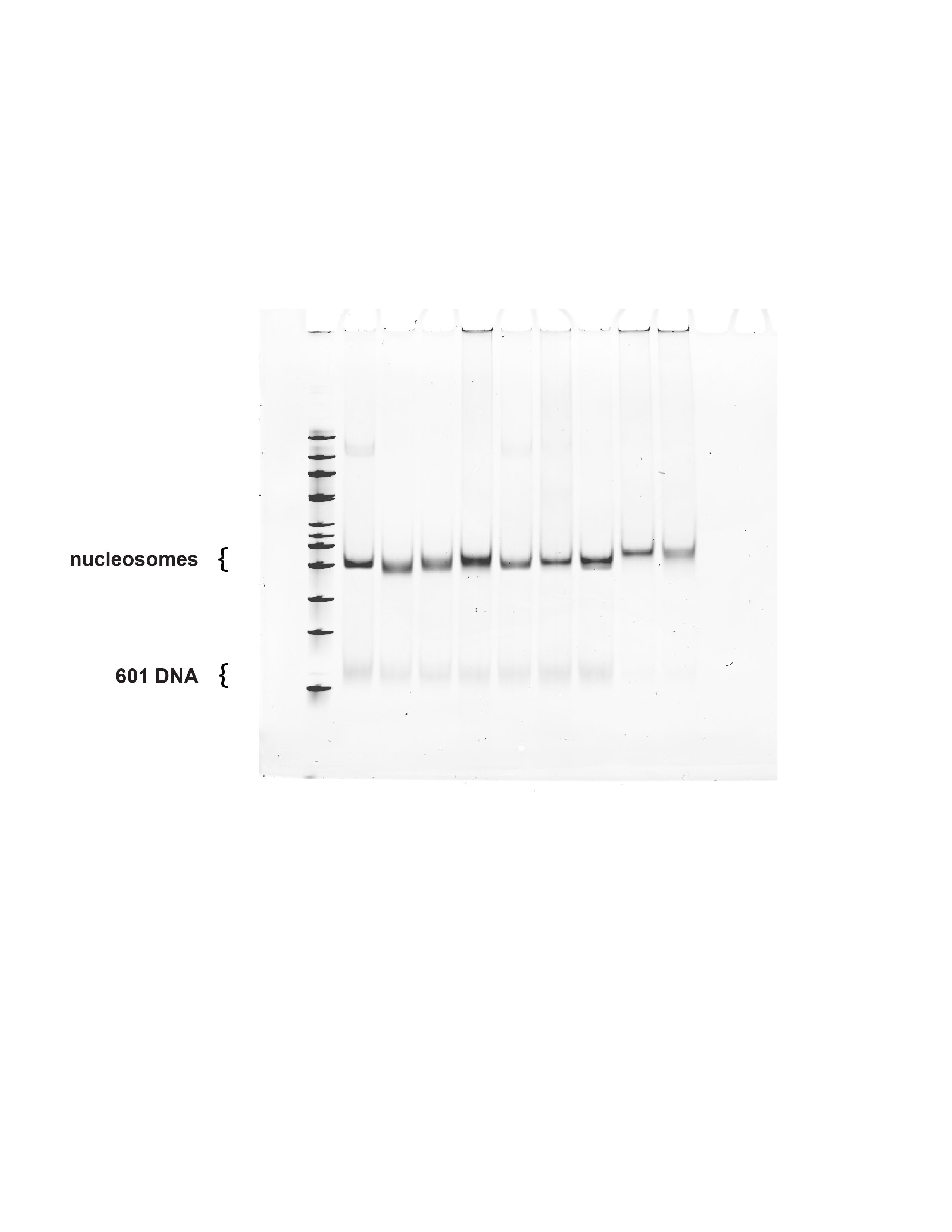

Supplement: Figure 4—source data 1. [file elife-71502-fig4-data1.zip › Figure 4-source data 1/Figure 4F-source data 1.tif]

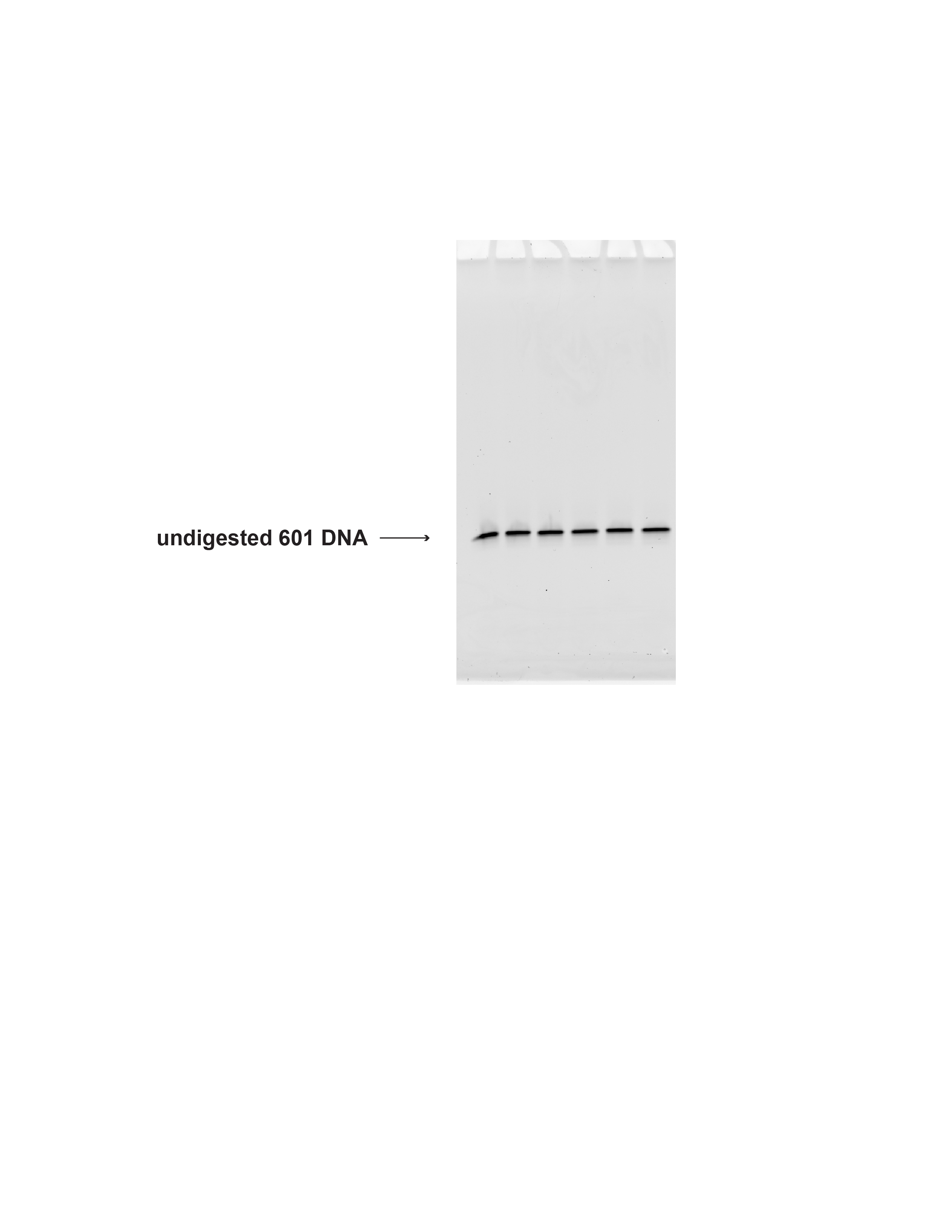

Supplement: Figure 5—source data 1. [file elife-71502-fig5-data1.zip › Figure 5A-source data 1.tif]

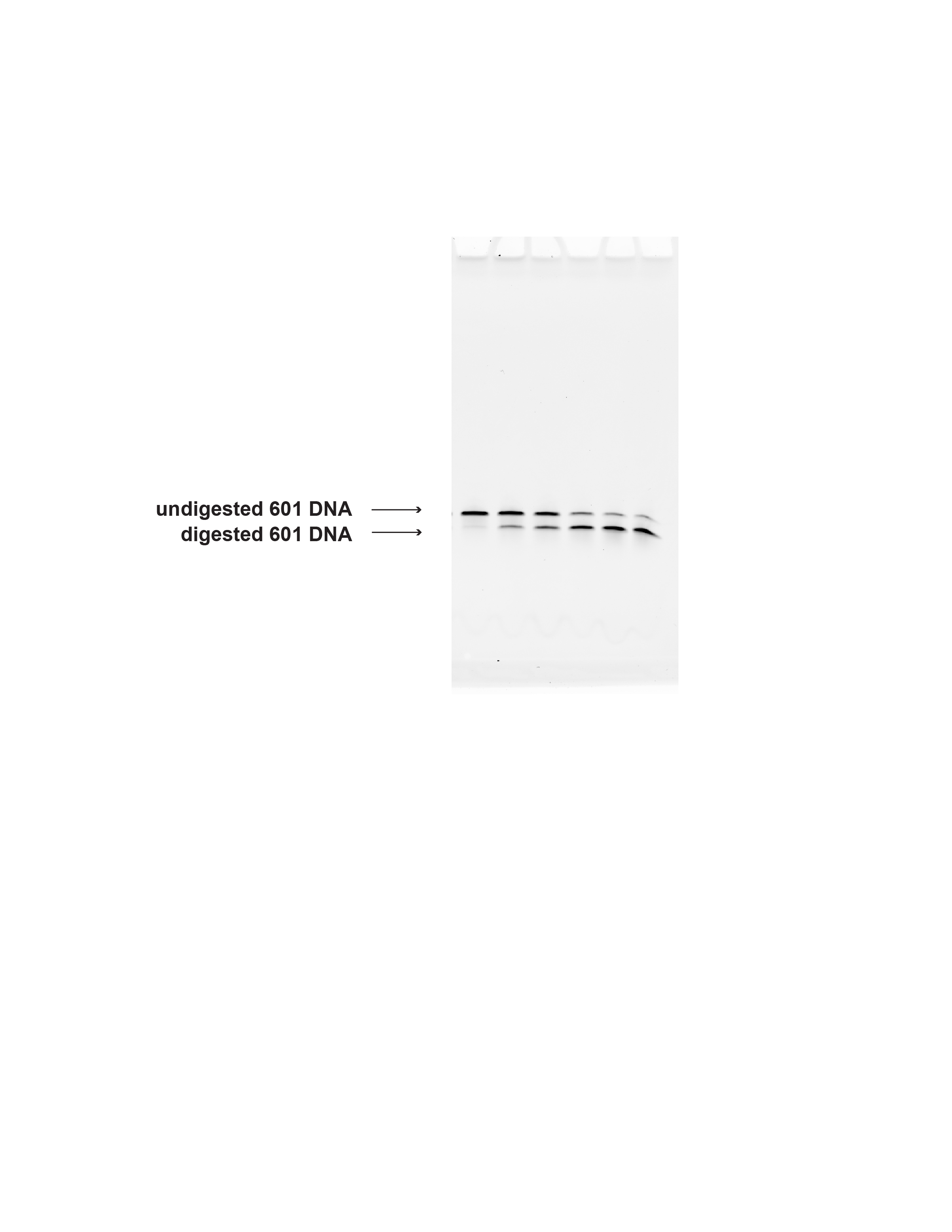

Supplement: Figure 5—source data 1. [file elife-71502-fig5-data1.zip › Figure 5A-source data 2.tif]

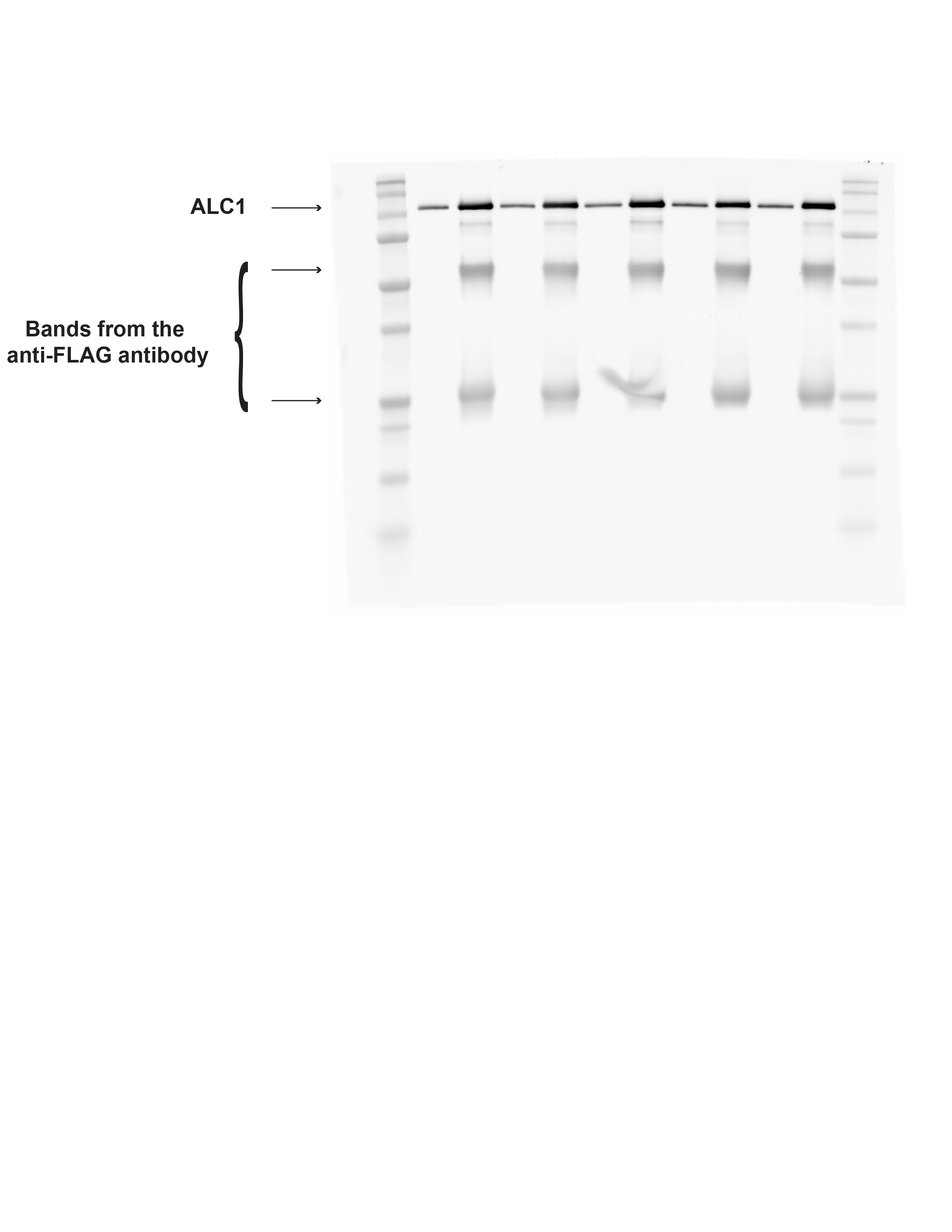

Supplement: Figure 5—source data 1. [file elife-71502-fig5-data1.zip › Figure 5D-source data 1.tif]

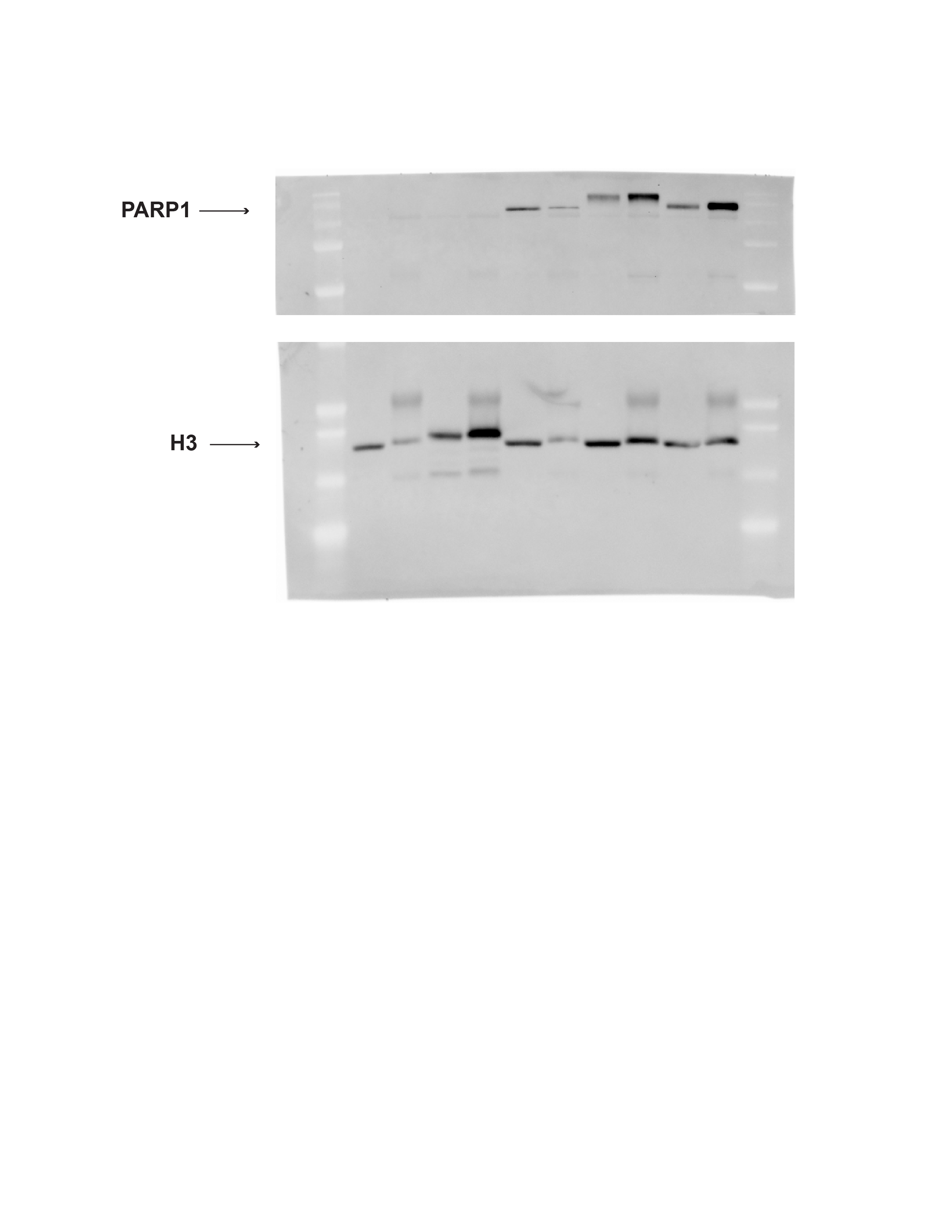

Supplement: Figure 5—source data 1. [file elife-71502-fig5-data1.zip › Figure 5D-source data 2.tif]

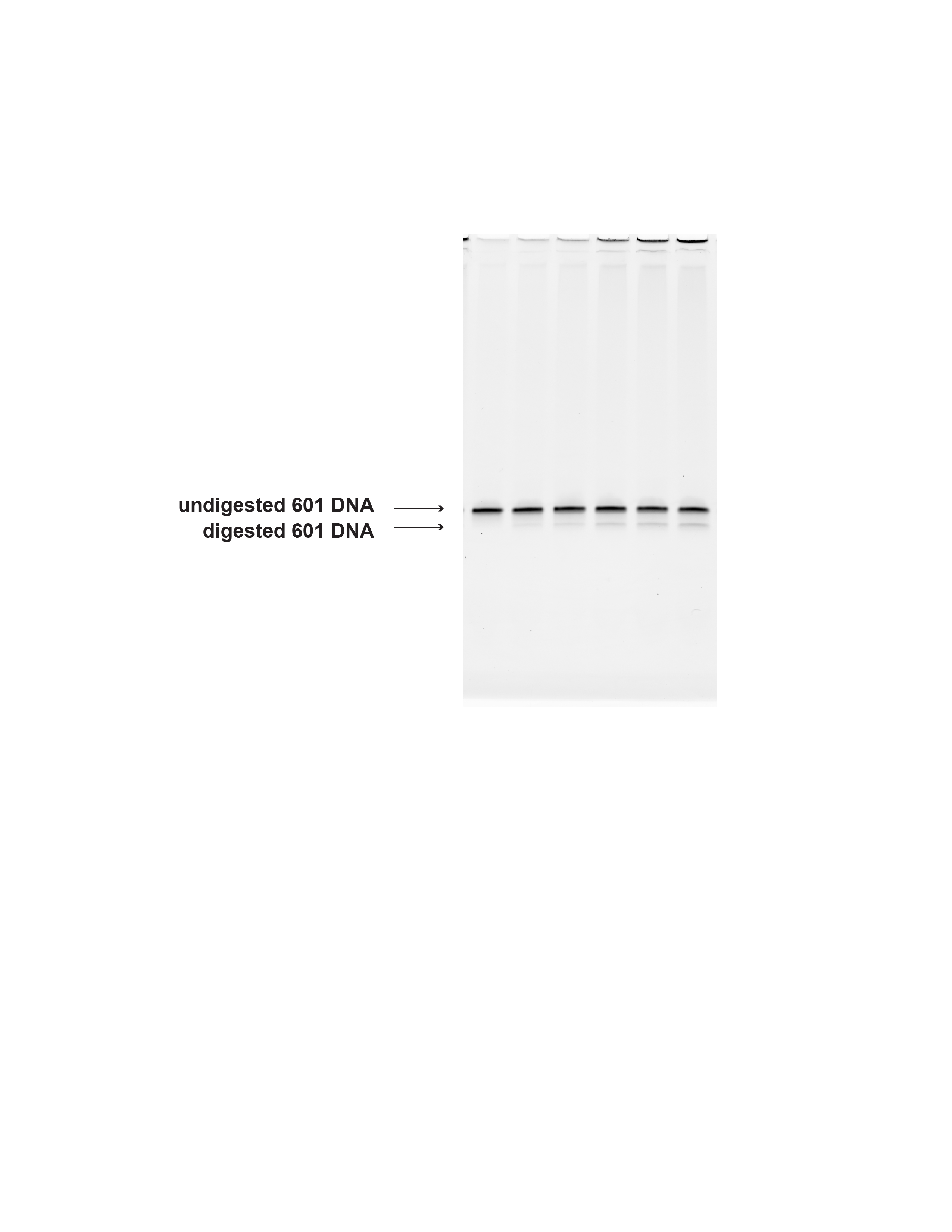

Supplement: Figure 5—figure supplement 1—source data 1. [file elife-71502-fig5-figsupp1-data1.zip › Figure 5-figure supplement 1-source data 1/Figure 5-figure supplement 1A-source data 5.tif]

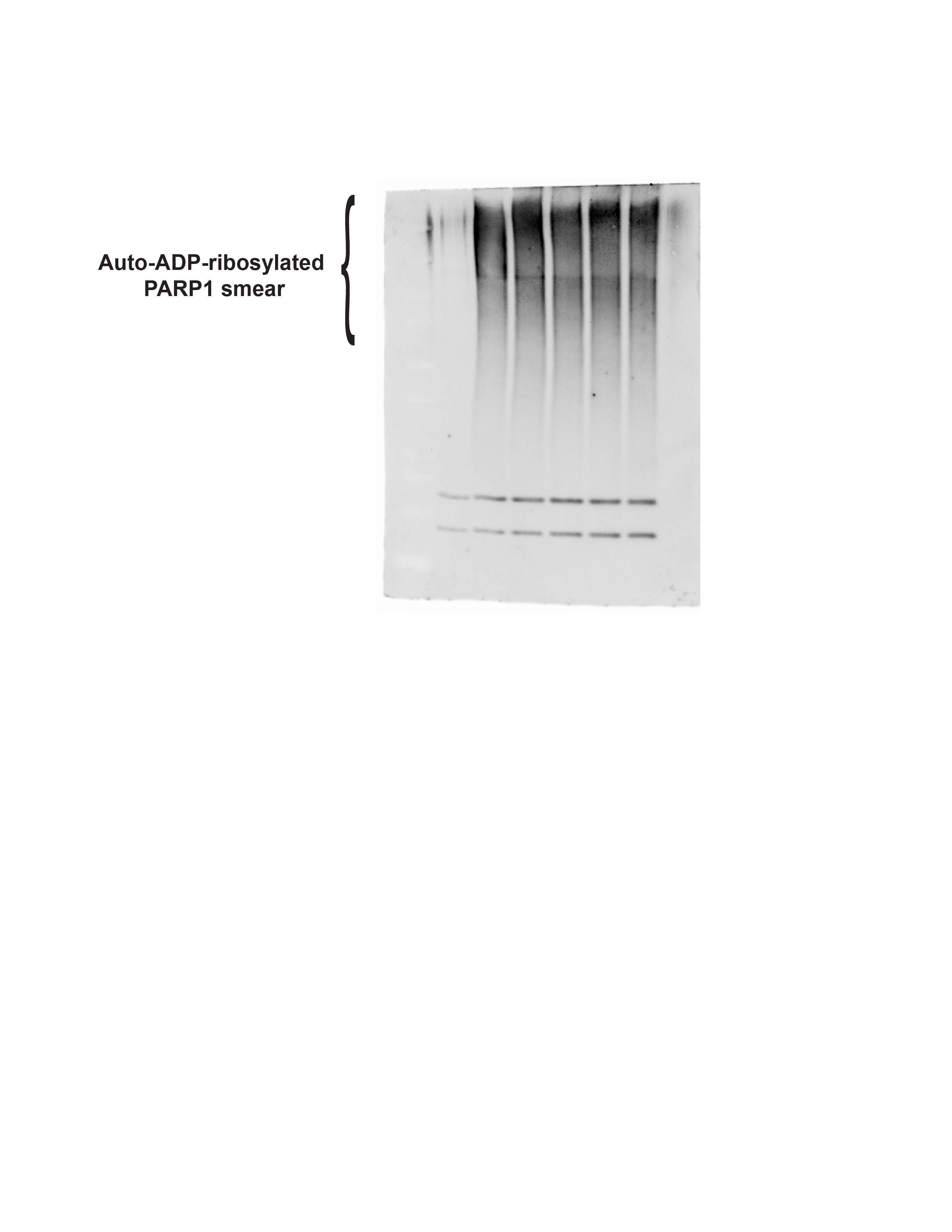

Supplement: Figure 5—figure supplement 1—source data 1. [file elife-71502-fig5-figsupp1-data1.zip › Figure 5-figure supplement 1-source data 1/Figure 5-figure supplement 1E-source data 1.tif]

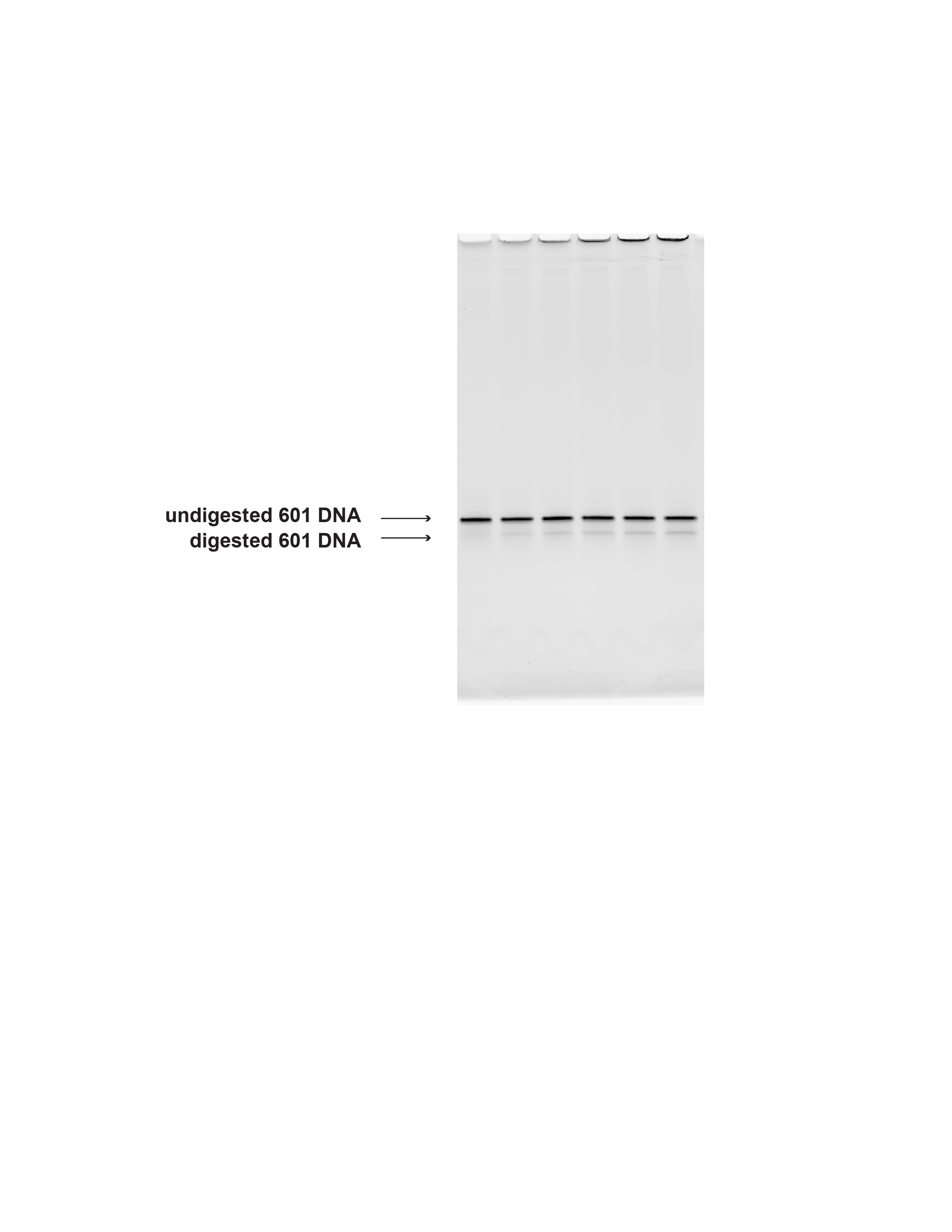

Supplement: Figure 5—figure supplement 1—source data 1. [file elife-71502-fig5-figsupp1-data1.zip › Figure 5-figure supplement 1-source data 1/Figure 5-figure supplement 1A-source data 4.tif]

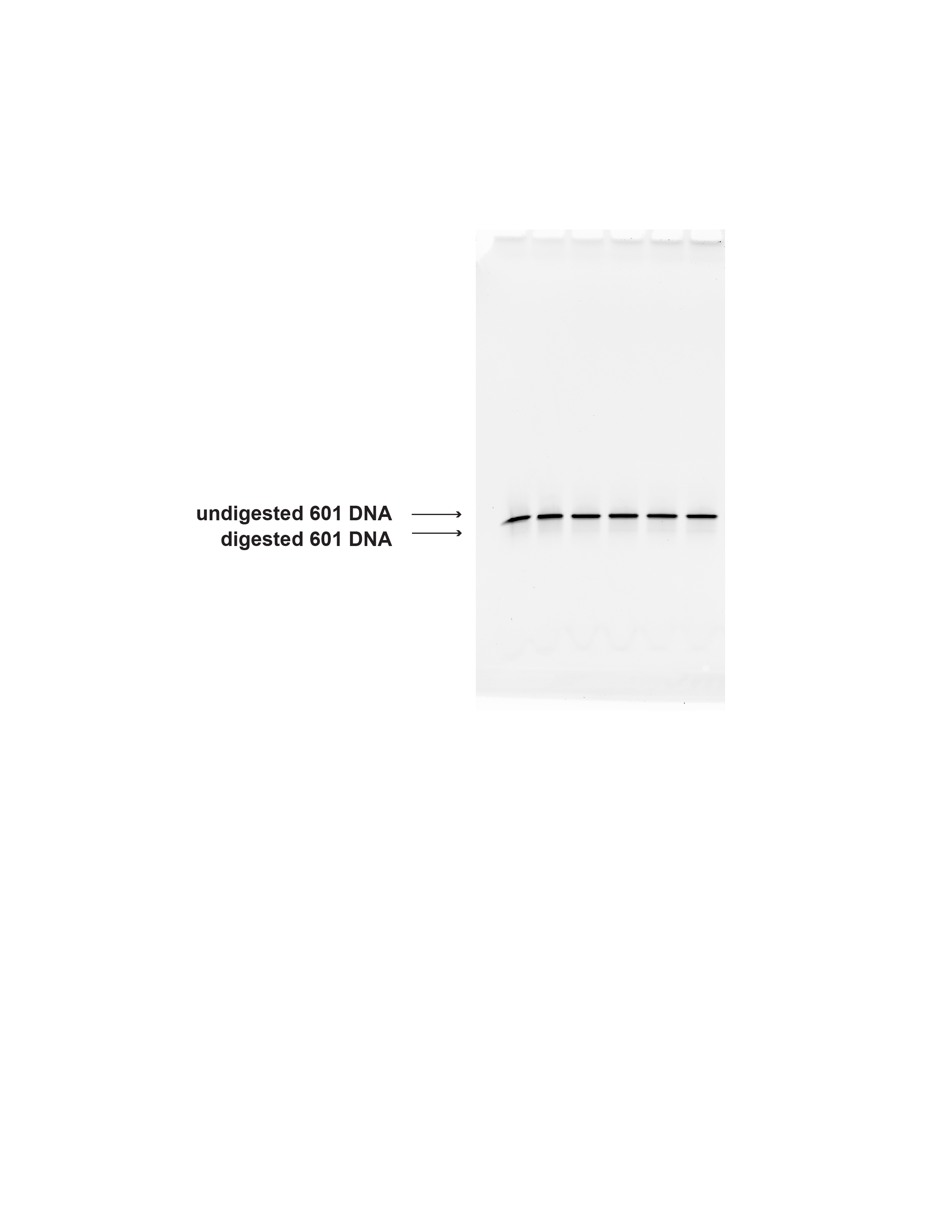

Supplement: Figure 5—figure supplement 1—source data 1. [file elife-71502-fig5-figsupp1-data1.zip › Figure 5-figure supplement 1-source data 1/Figure 5-figure supplement 1A-source data 6.tif]

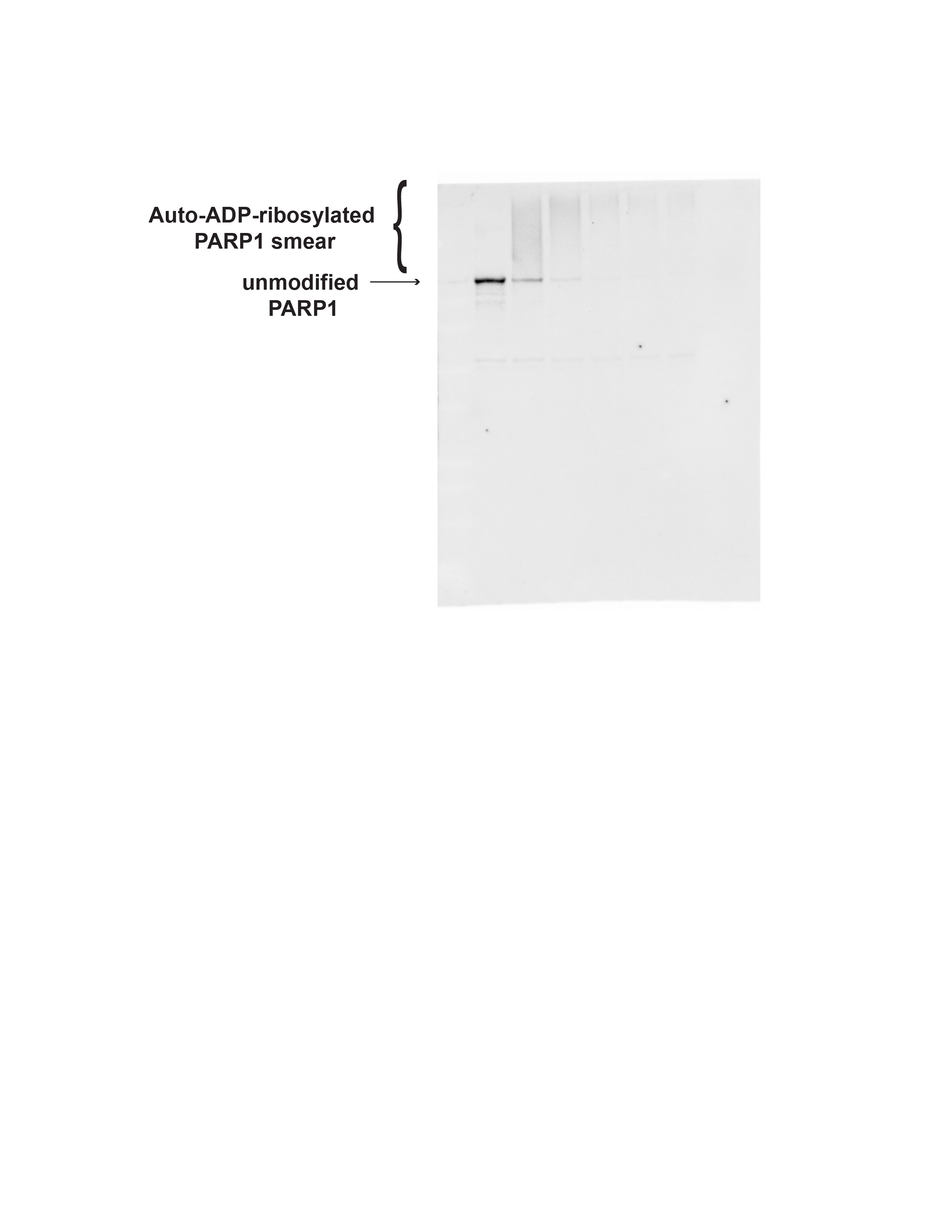

Supplement: Figure 5—figure supplement 1—source data 1. [file elife-71502-fig5-figsupp1-data1.zip › Figure 5-figure supplement 1-source data 1/Figure 5-figure supplement 1E-source data 2.tif]

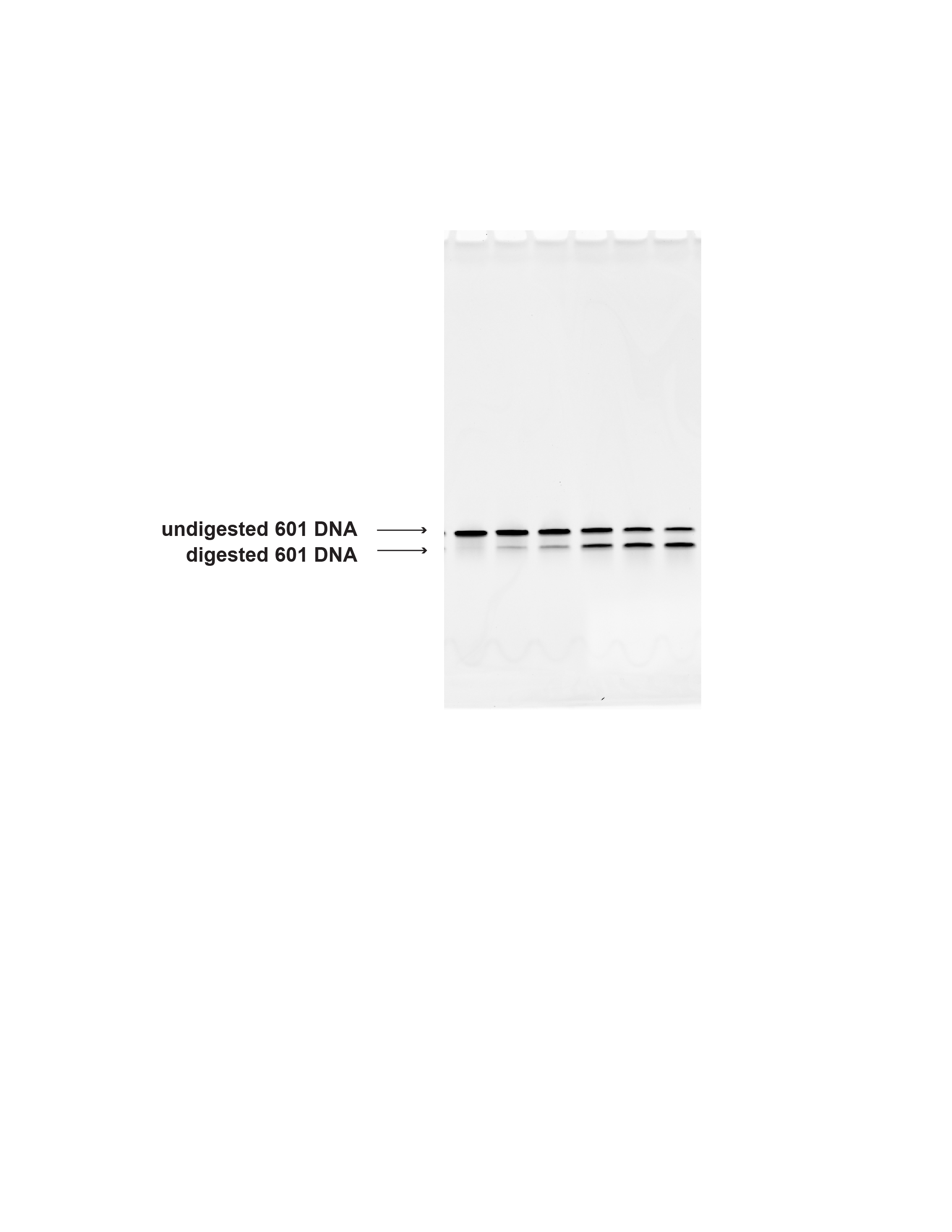

Supplement: Figure 5—figure supplement 1—source data 1. [file elife-71502-fig5-figsupp1-data1.zip › Figure 5-figure supplement 1-source data 1/Figure 5-figure supplement 1A-source data 7.tif]

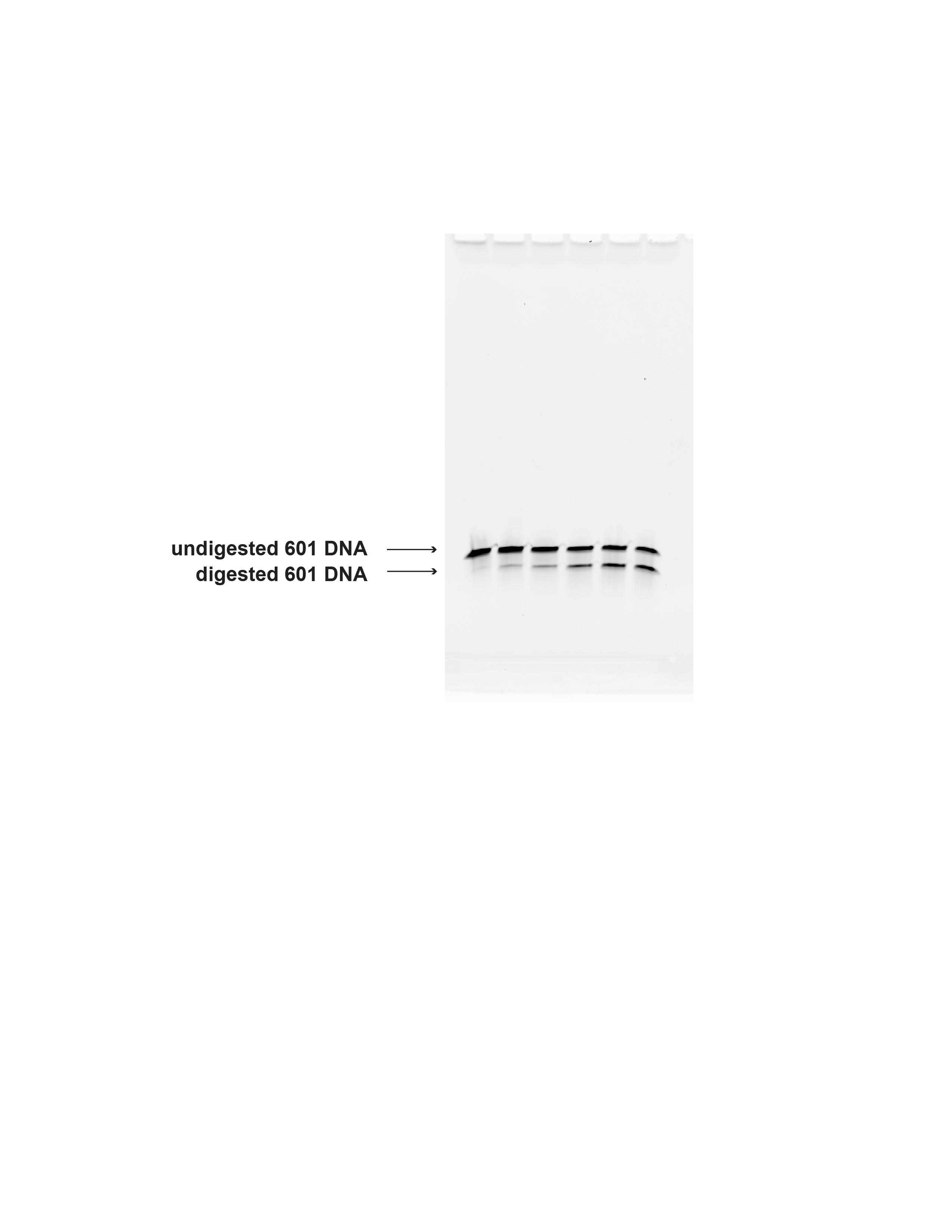

Supplement: Figure 5—figure supplement 1—source data 1. [file elife-71502-fig5-figsupp1-data1.zip › Figure 5-figure supplement 1-source data 1/Figure 5-figure supplement 1A-source data 3.tif]

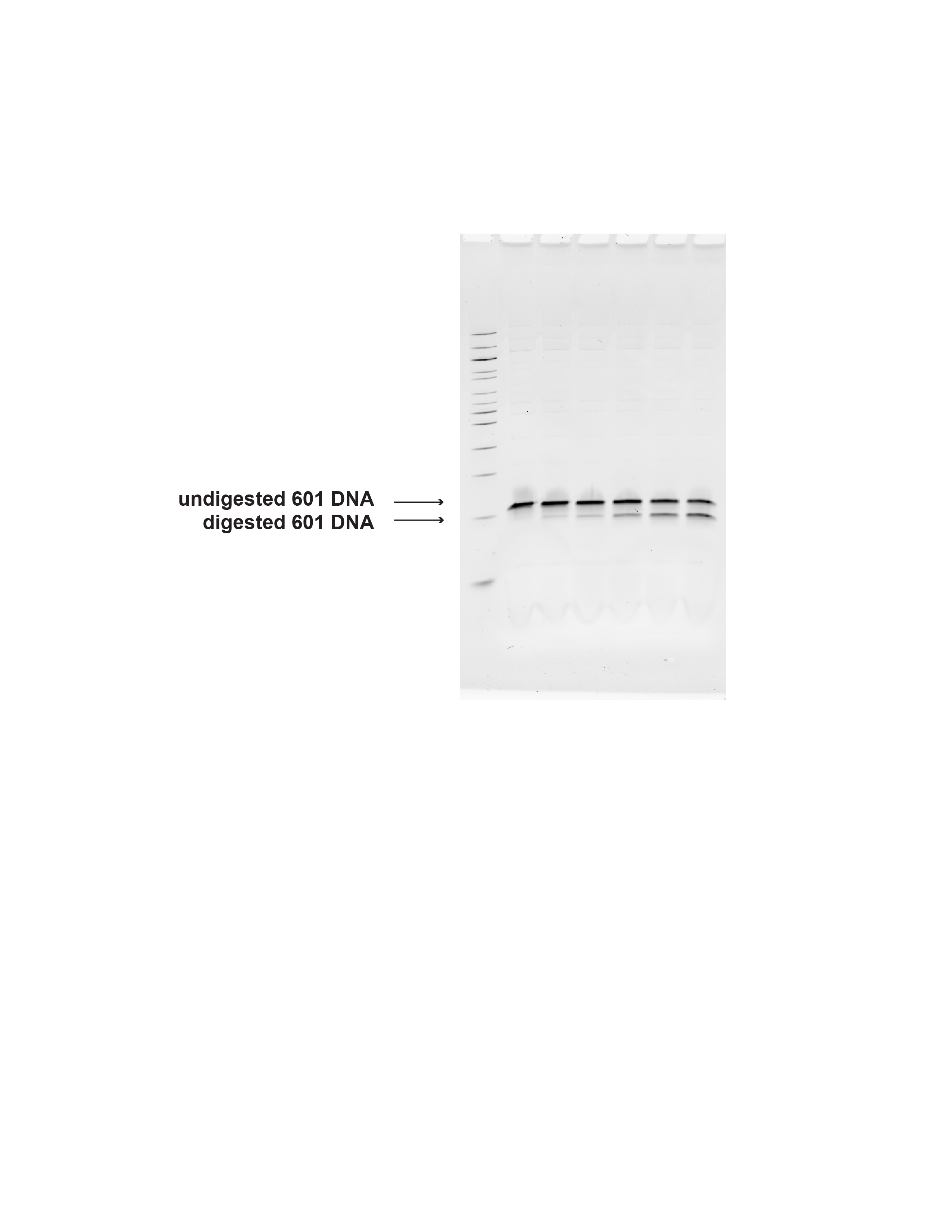

Supplement: Figure 5—figure supplement 1—source data 1. [file elife-71502-fig5-figsupp1-data1.zip › Figure 5-figure supplement 1-source data 1/Figure 5-figure supplement 1A-source data 2.tif]

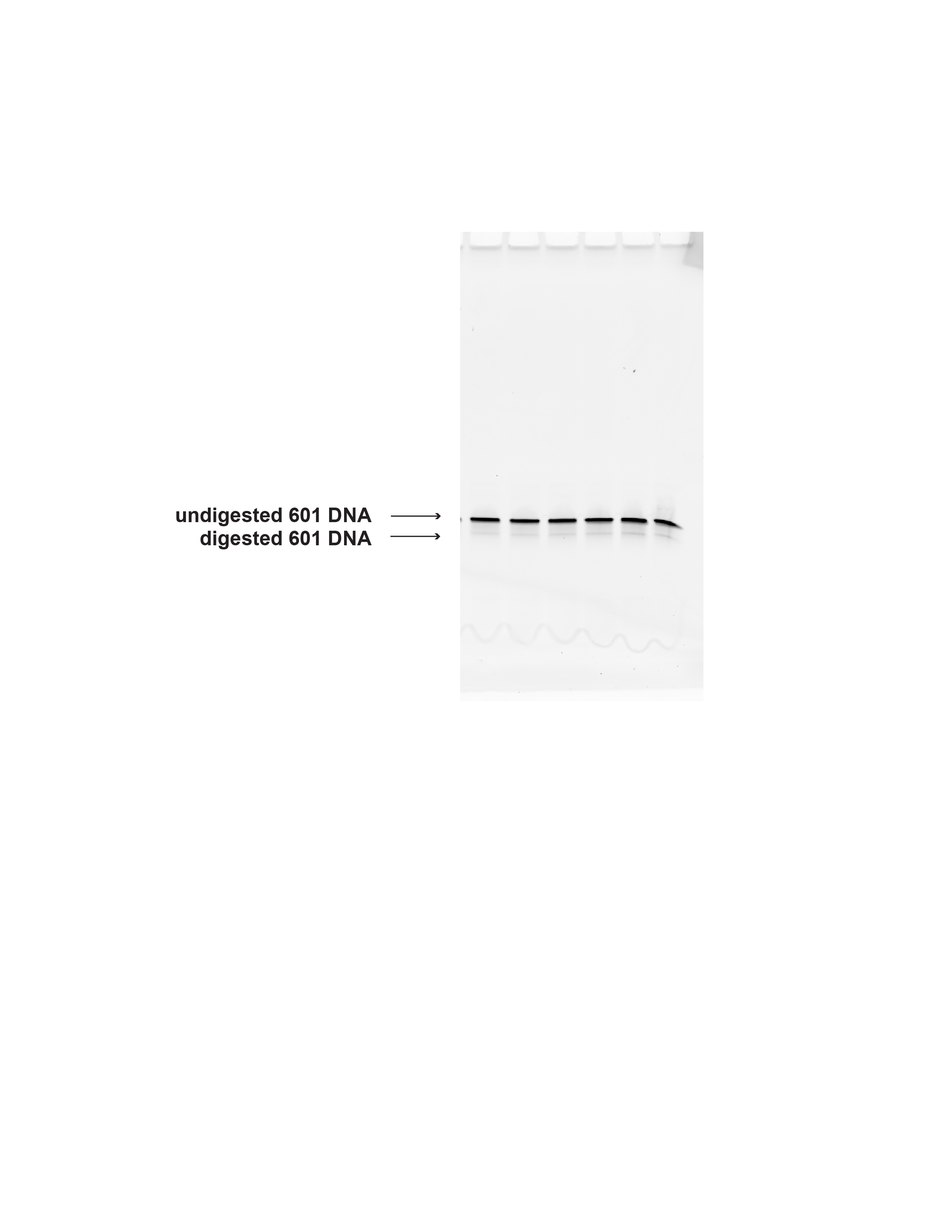

Supplement: Figure 5—figure supplement 1—source data 1. [file elife-71502-fig5-figsupp1-data1.zip › Figure 5-figure supplement 1-source data 1/Figure 5-figure supplement 1A-source data 1.tif]

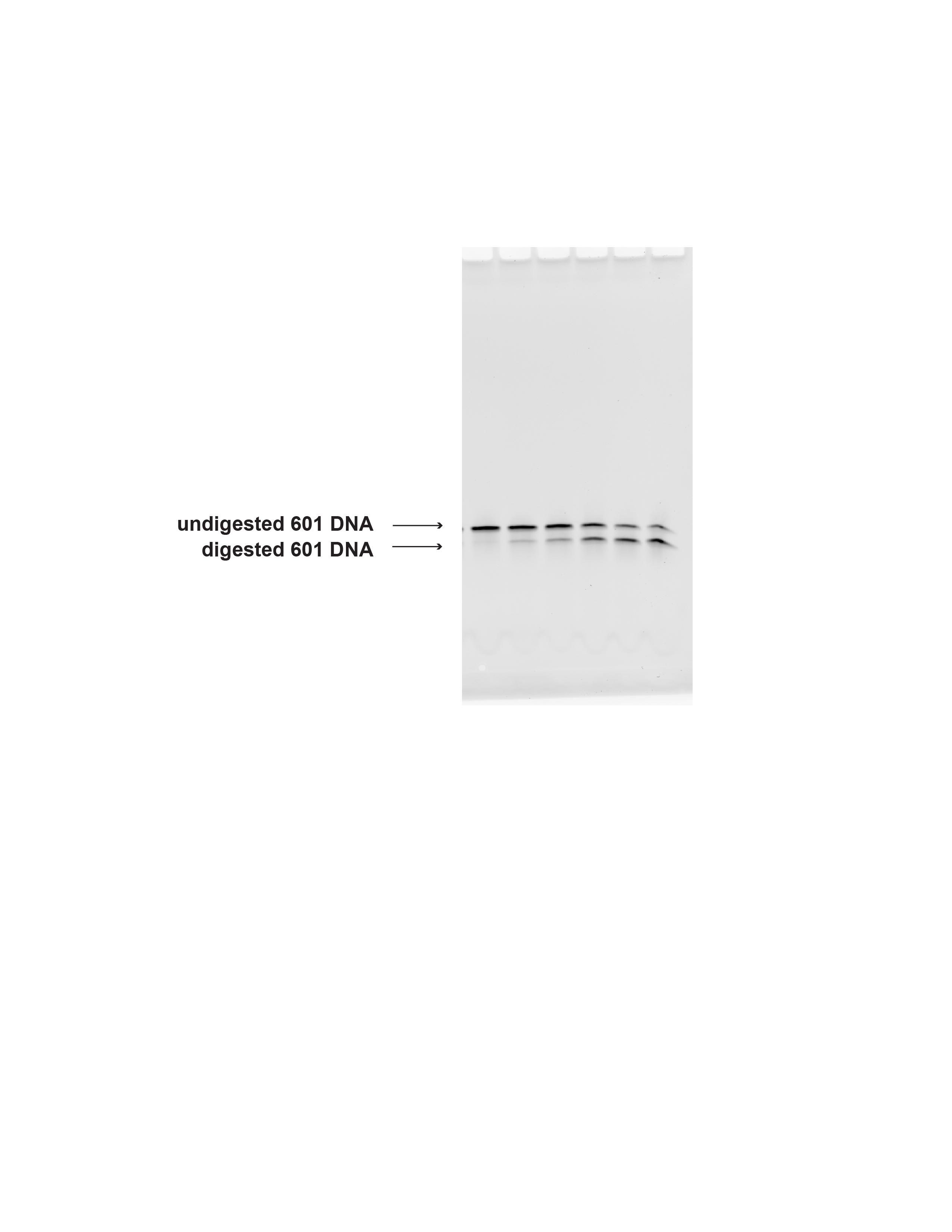

Supplement: Figure 5—figure supplement 1—source data 1. [file elife-71502-fig5-figsupp1-data1.zip › Figure 5-figure supplement 1-source data 1/Figure 5-figure supplement 1A-source data 9.tif]

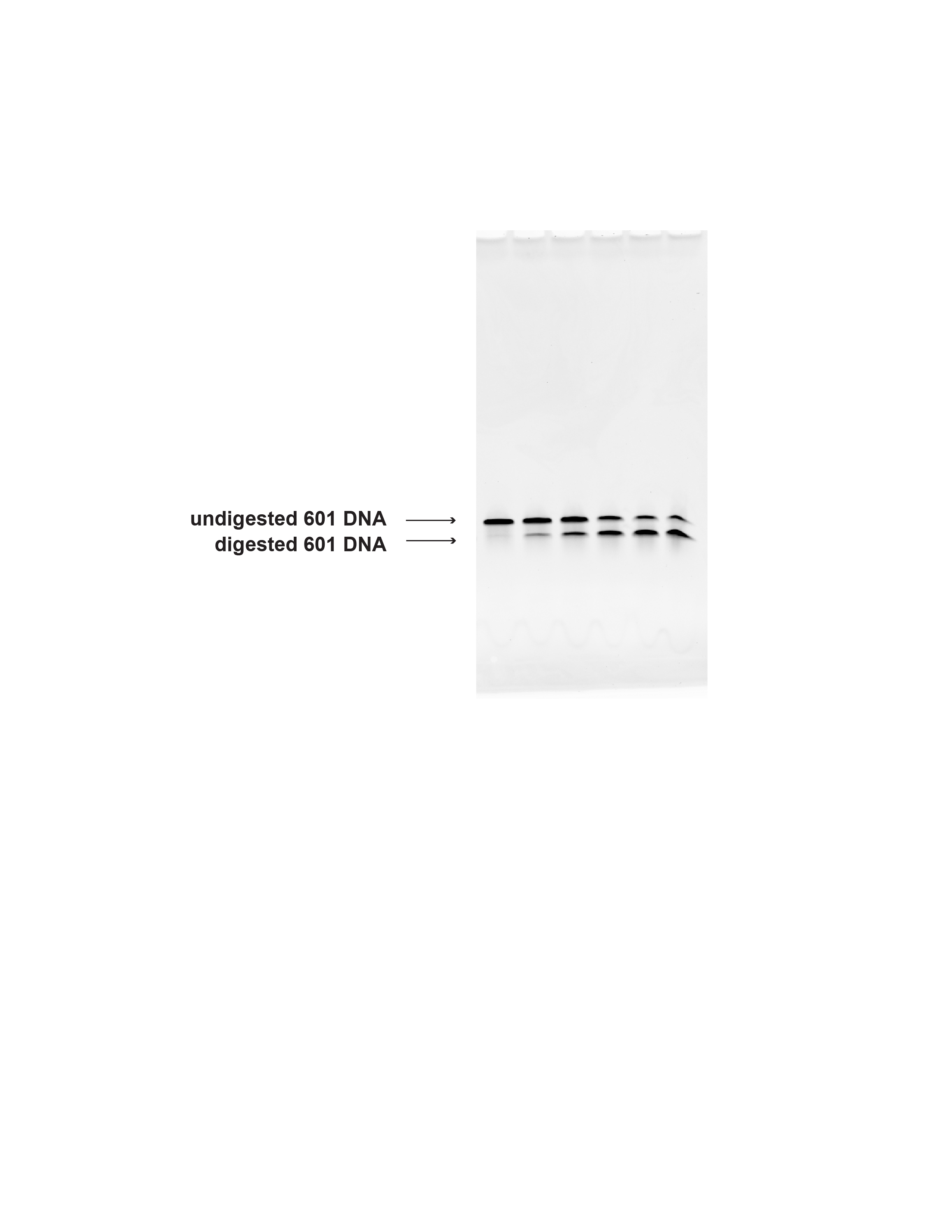

Supplement: Figure 5—figure supplement 1—source data 1. [file elife-71502-fig5-figsupp1-data1.zip › Figure 5-figure supplement 1-source data 1/Figure 5-figure supplement 1A-source data 8.tif]

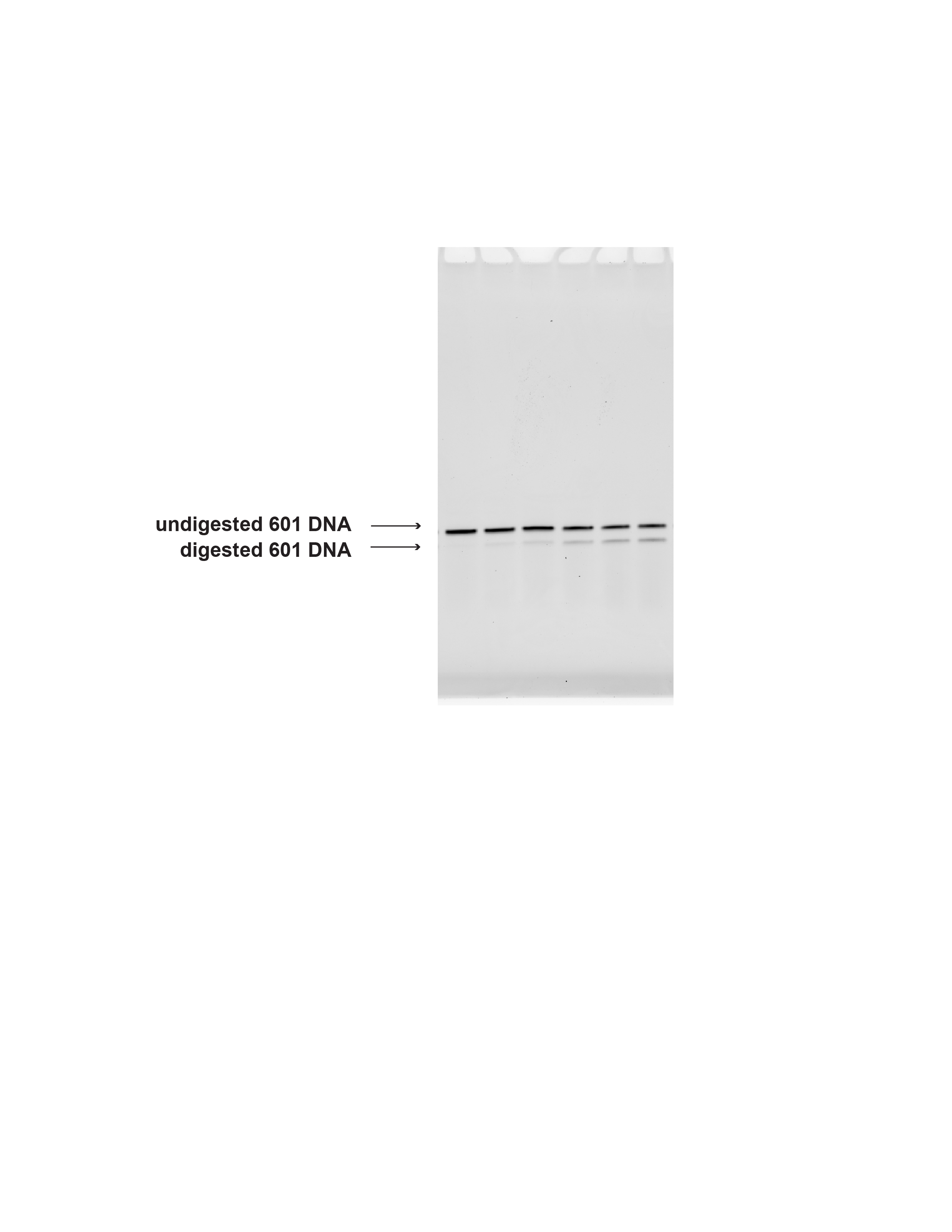

Supplement: Figure 5—figure supplement 1—source data 1. [file elife-71502-fig5-figsupp1-data1.zip › Figure 5-figure supplement 1-source data 1/Figure 5-figure supplement 1A-source data 10.tif]

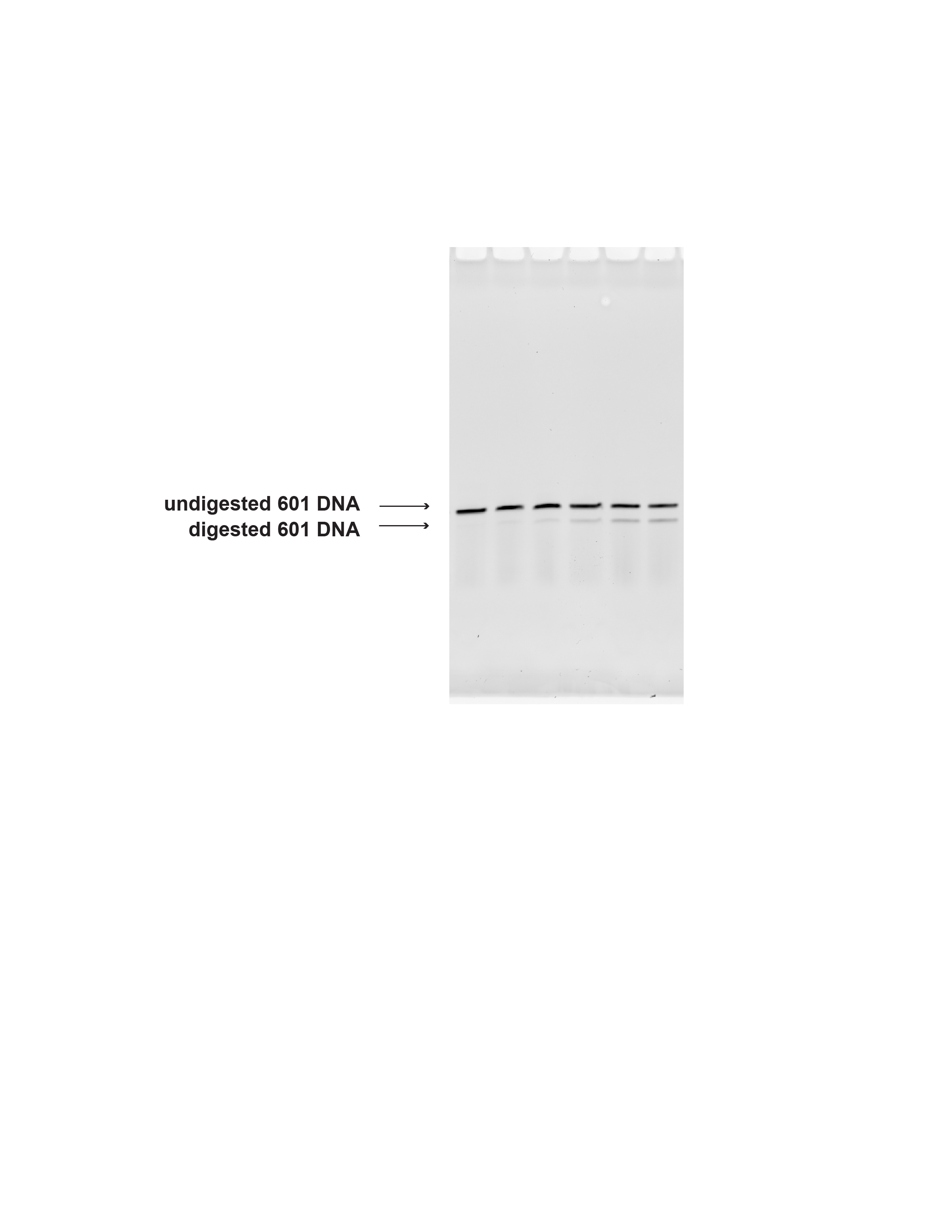

Supplement: Figure 5—figure supplement 1—source data 1. [file elife-71502-fig5-figsupp1-data1.zip › Figure 5-figure supplement 1-source data 1/Figure 5-figure supplement 1A-source data 11.tif]

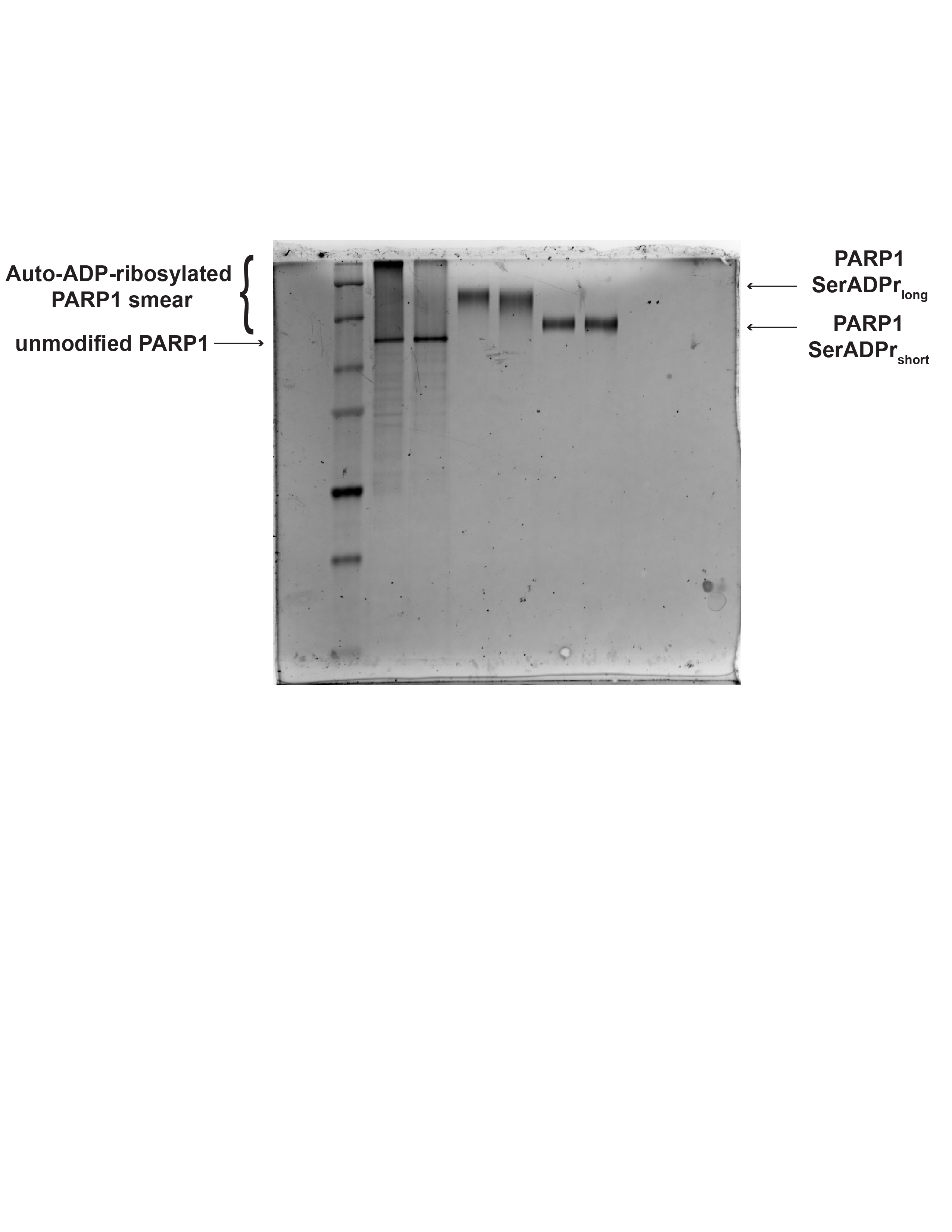

Supplement: Figure 5—figure supplement 2—source data 1. [file elife-71502-fig5-figsupp2-data1.zip › Figure 5-figure supplement 2-source data 1/Figure 5-figure supplement 2D-source data 1.tif]

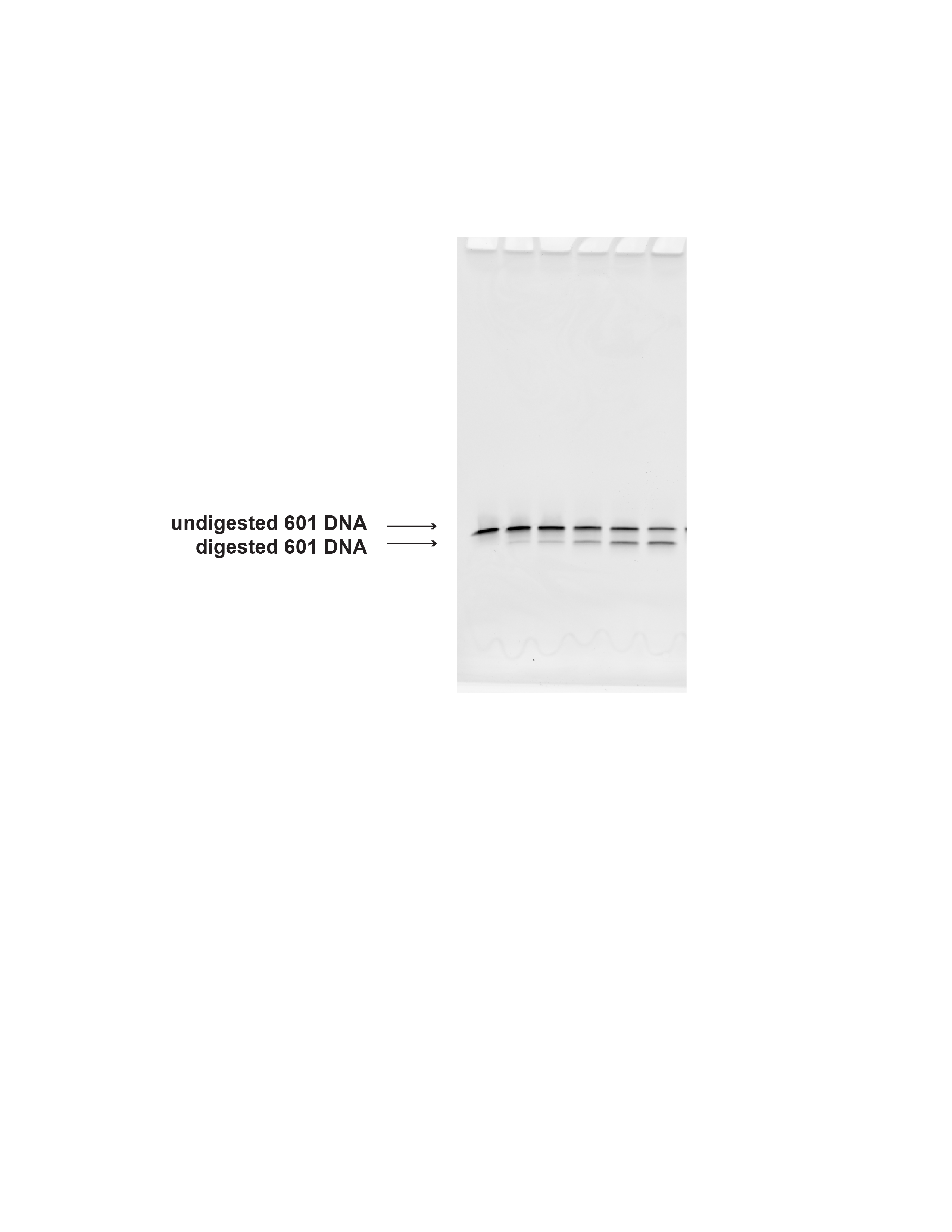

Supplement: Figure 7—source data 1. [file elife-71502-fig7-data1.zip › Figure 7A-source data 1.tif]

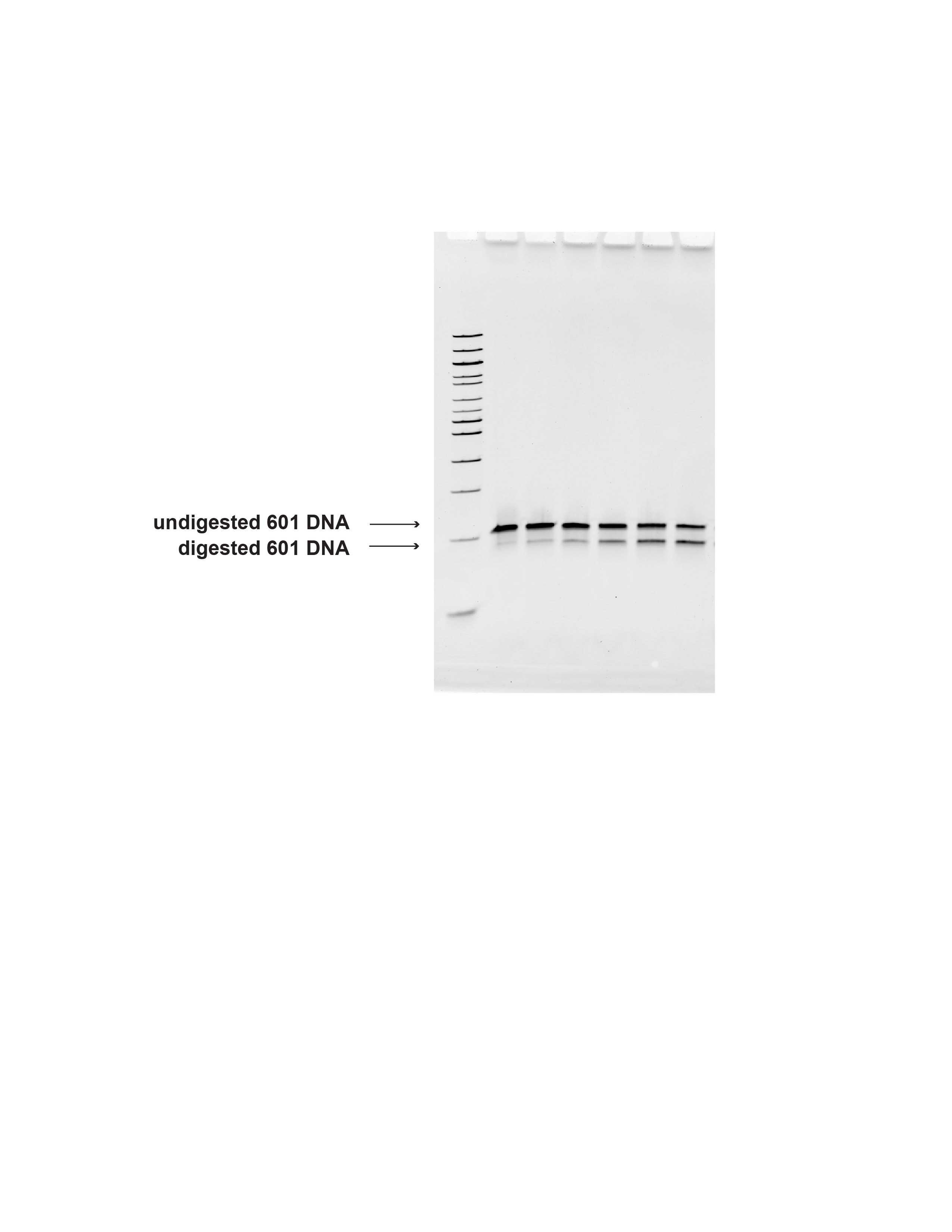

Supplement: Figure 7—source data 1. [file elife-71502-fig7-data1.zip › Figure 7A-source data 2.tif]

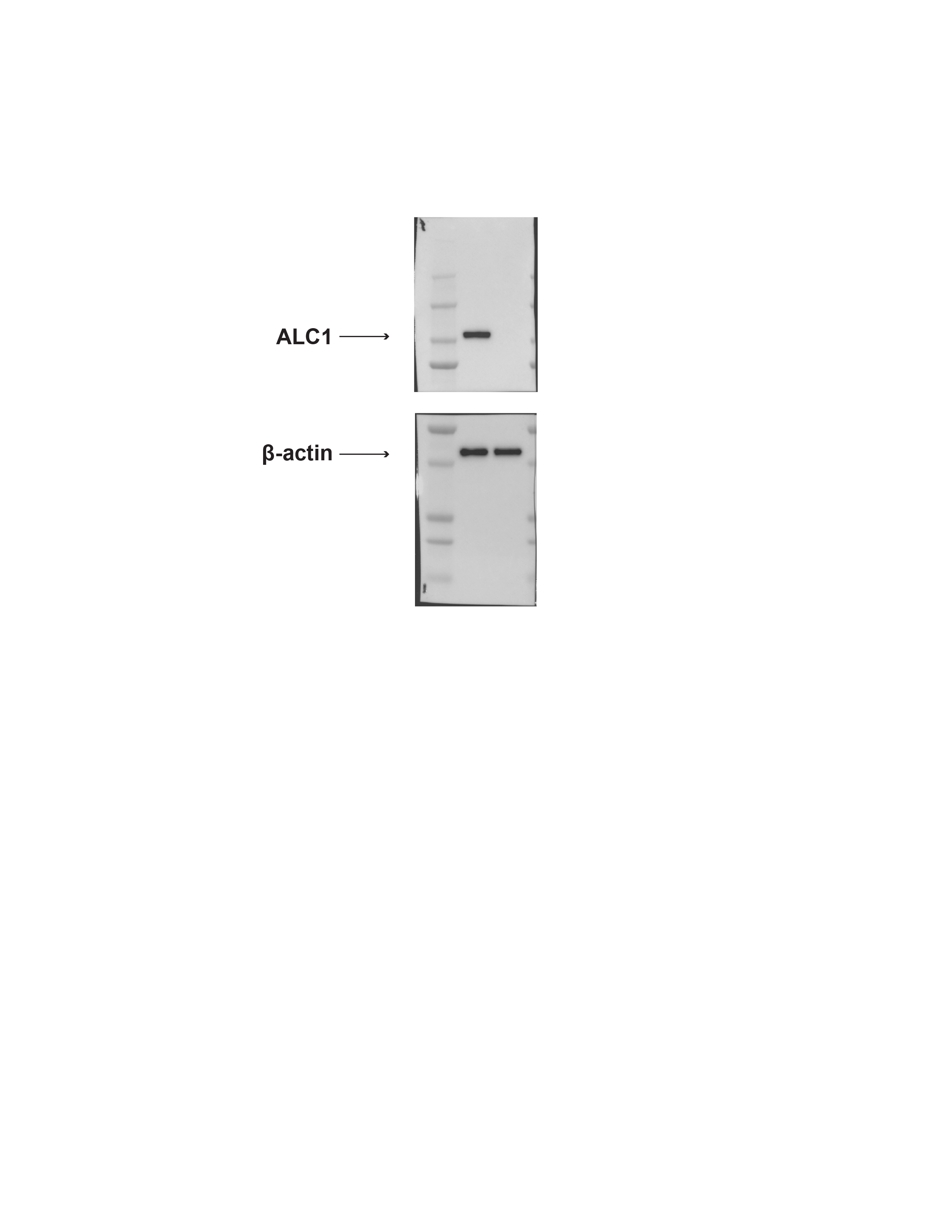

Supplement: Figure 7—source data 1. [file elife-71502-fig7-data1.zip › Figure 7C-source data 1.tif]

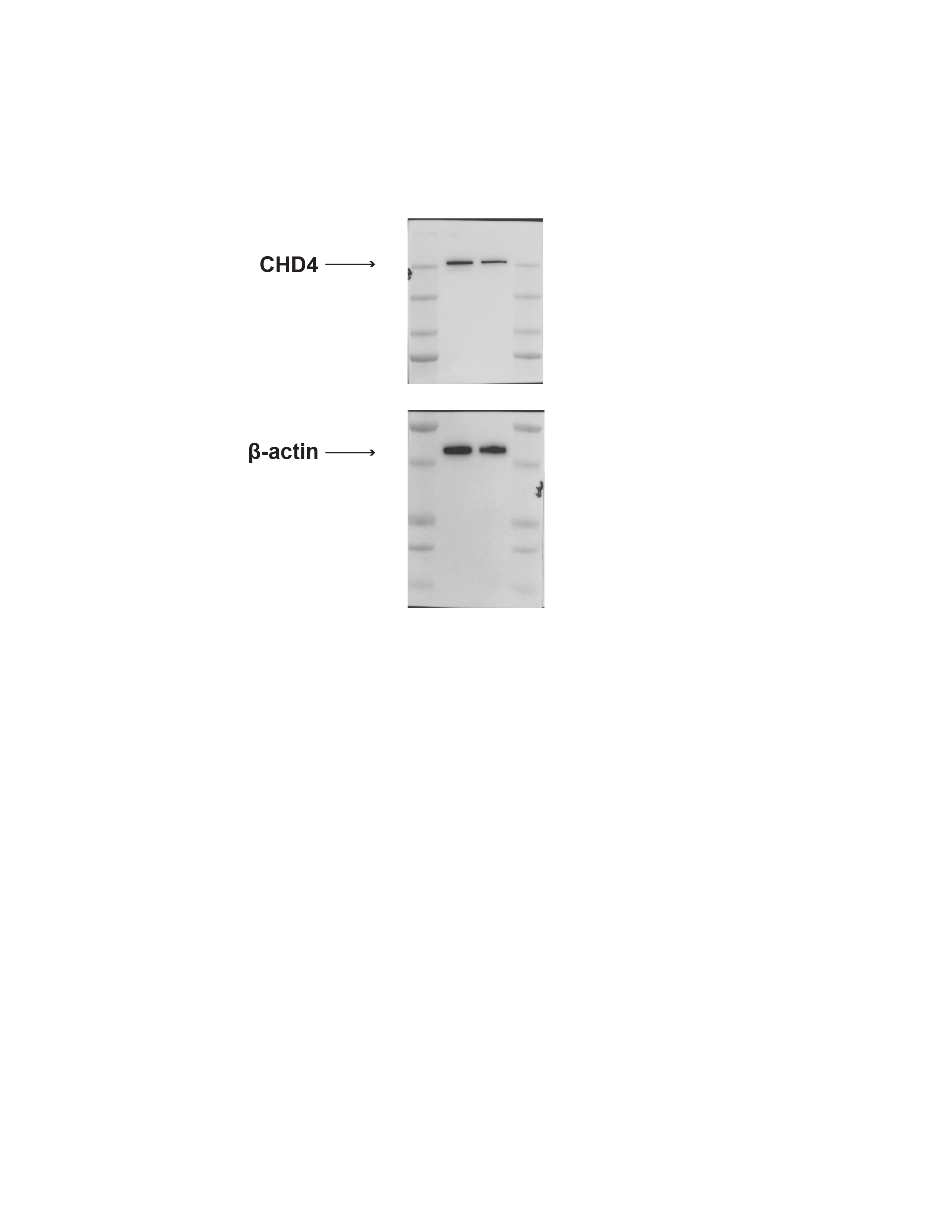

Supplement: Figure 7—source data 1. [file elife-71502-fig7-data1.zip › Figure 7C-source data 2.tif]

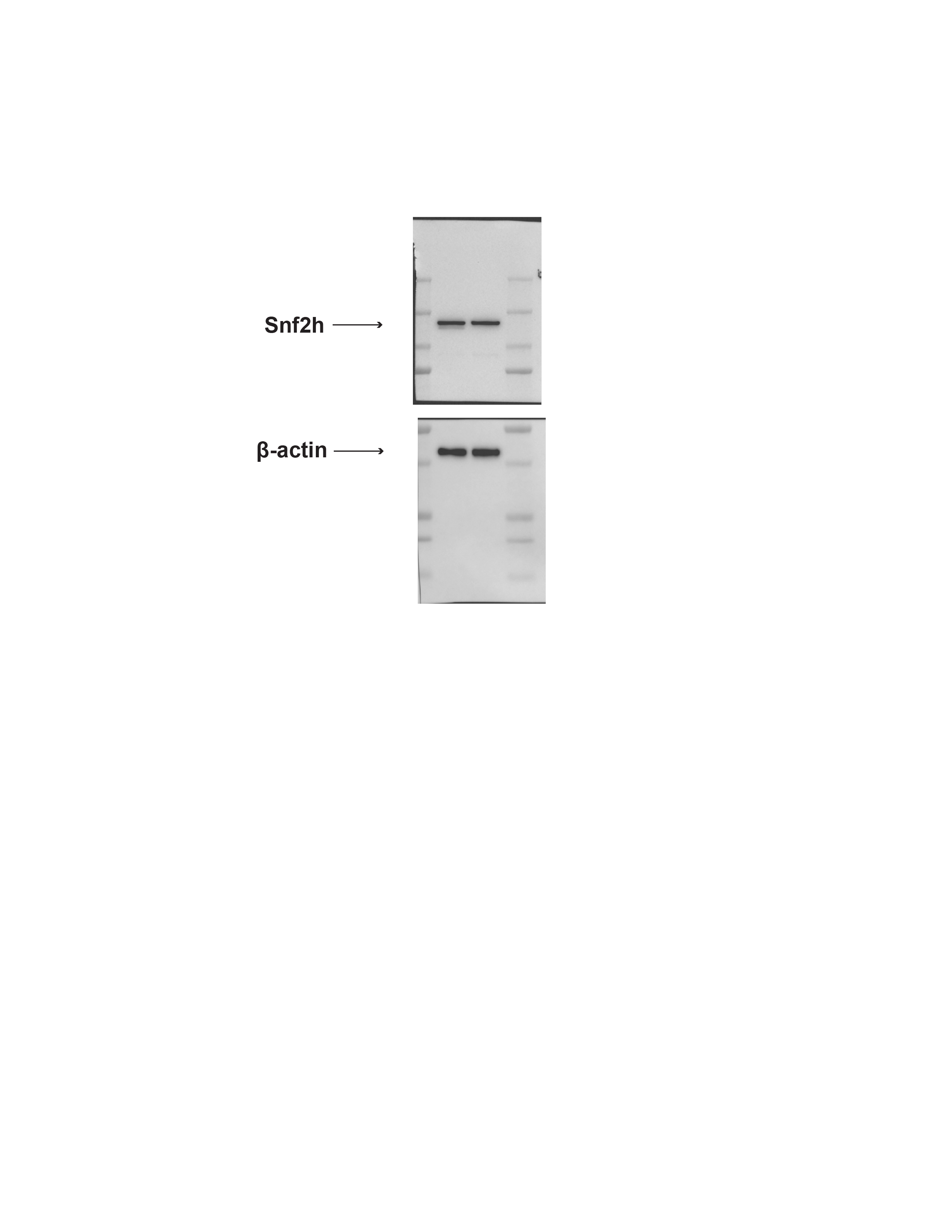

Supplement: Figure 7—source data 1. [file elife-71502-fig7-data1.zip › Figure 7C-source data 3.tif]

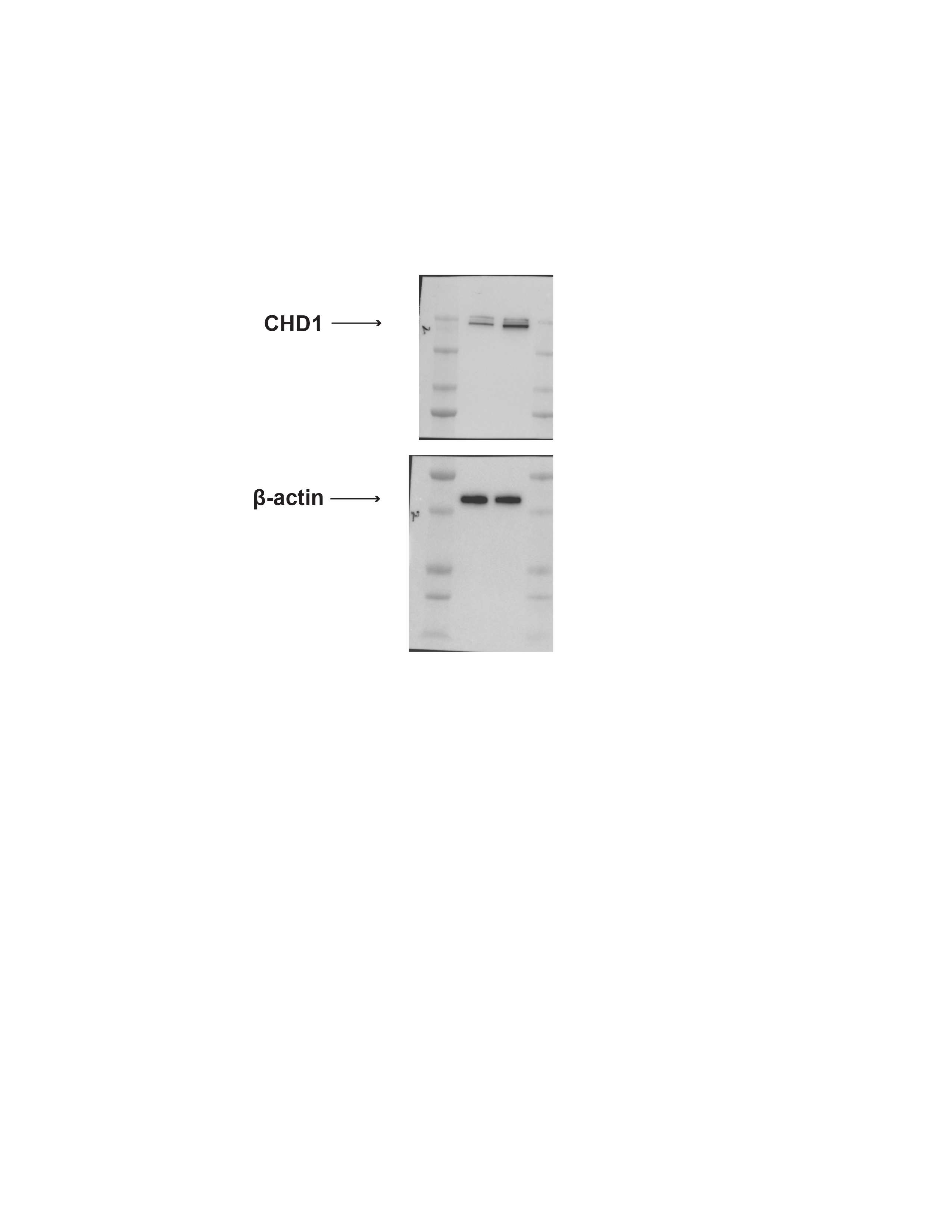

Supplement: Figure 7—source data 1. [file elife-71502-fig7-data1.zip › Figure 7C-source data 4.tif]

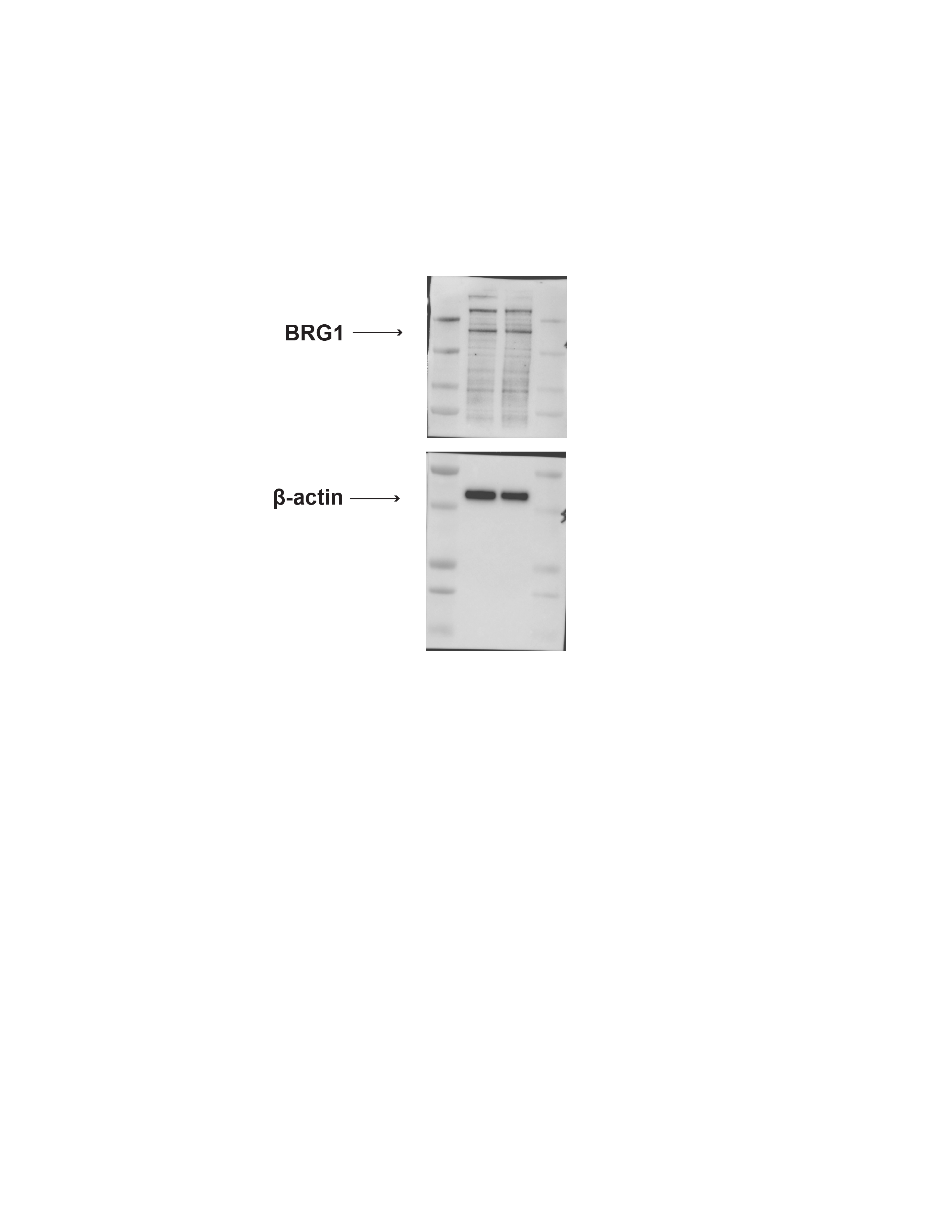

Supplement: Figure 7—source data 1. [file elife-71502-fig7-data1.zip › Figure 7C-source data 5.tif]

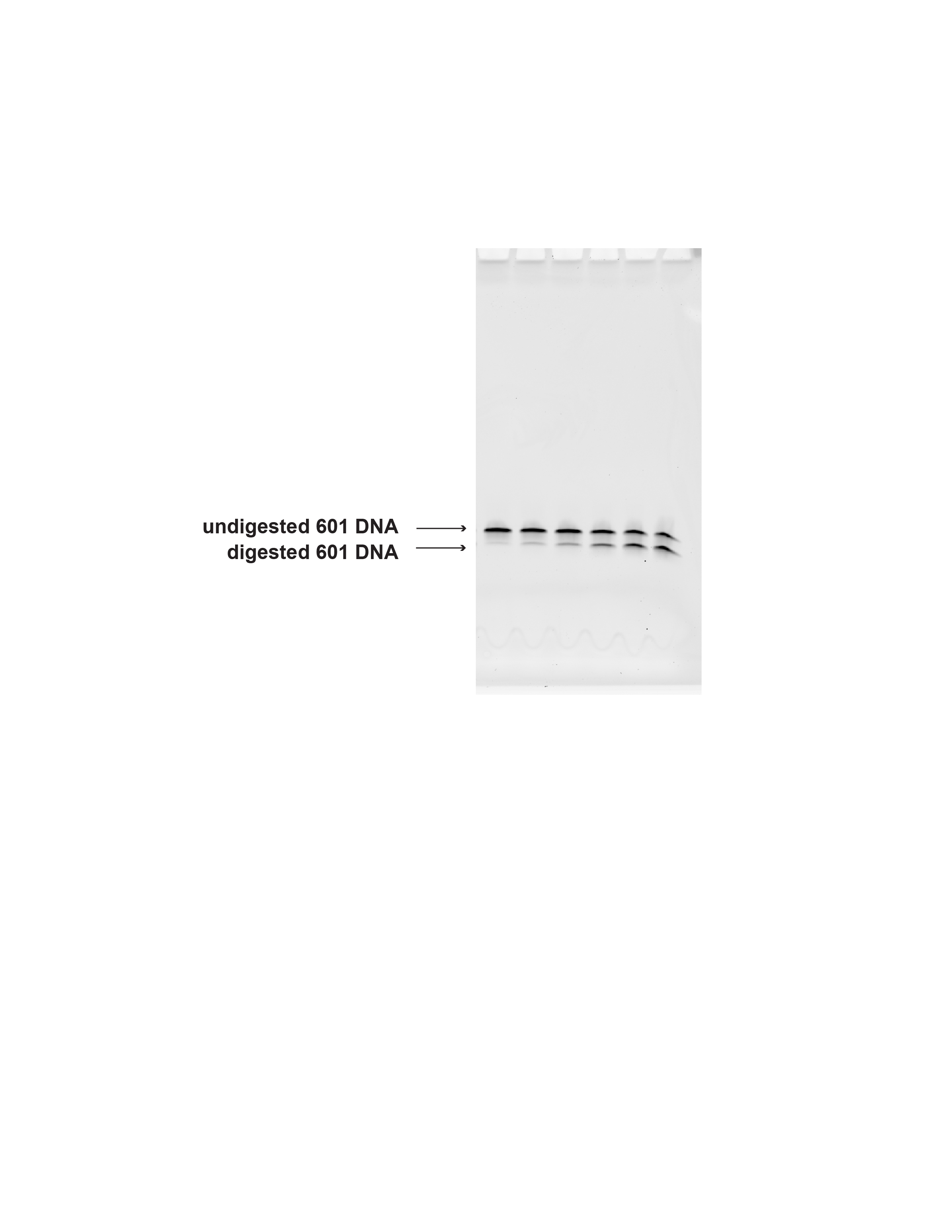

Supplement: Figure 7—figure supplement 1—source data 1. [file elife-71502-fig7-figsupp1-data1.zip › Figure 7-figure supplement 1B-source data 1.tif]

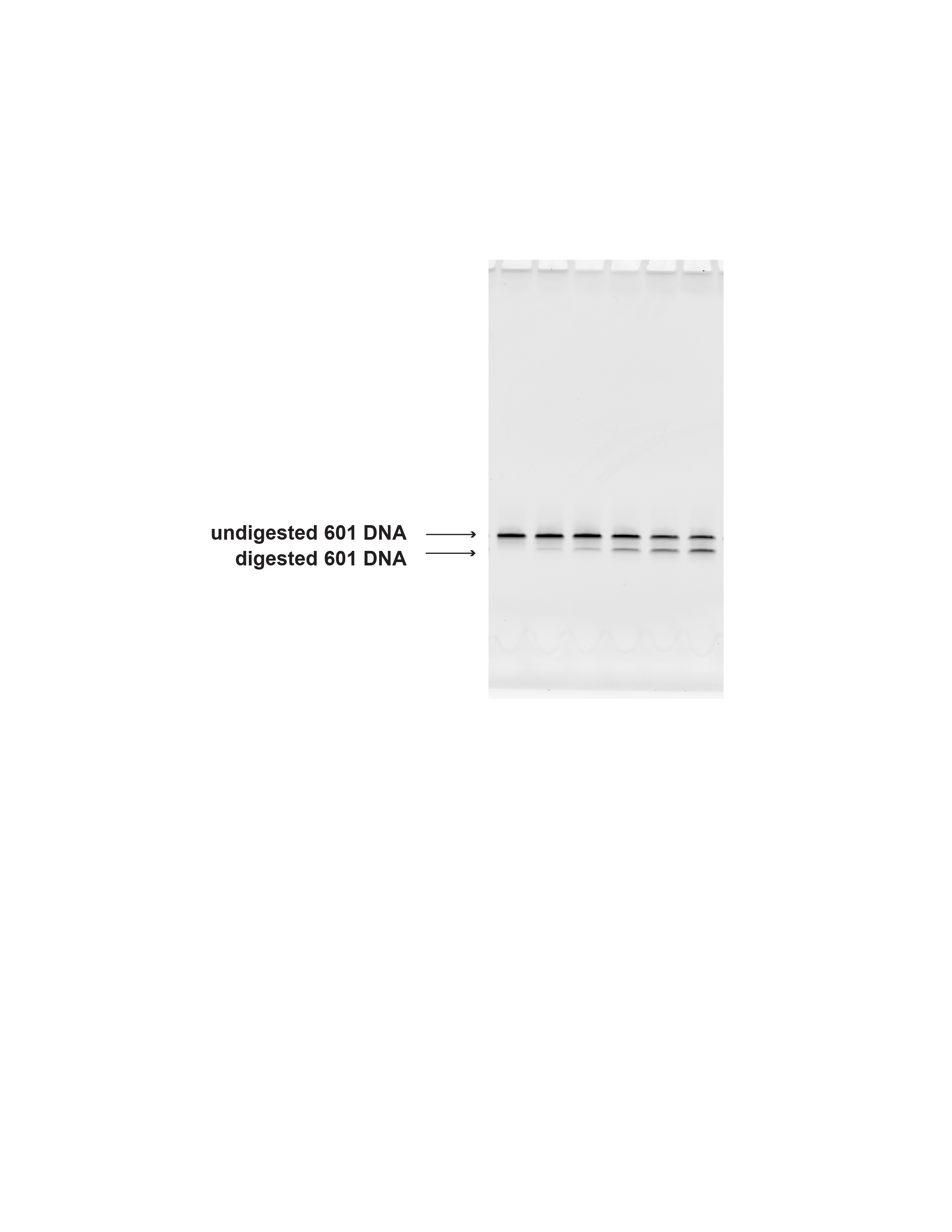

Supplement: Figure 7—figure supplement 1—source data 1. [file elife-71502-fig7-figsupp1-data1.zip › Figure 7-figure supplement 1B-source data 2.tif]

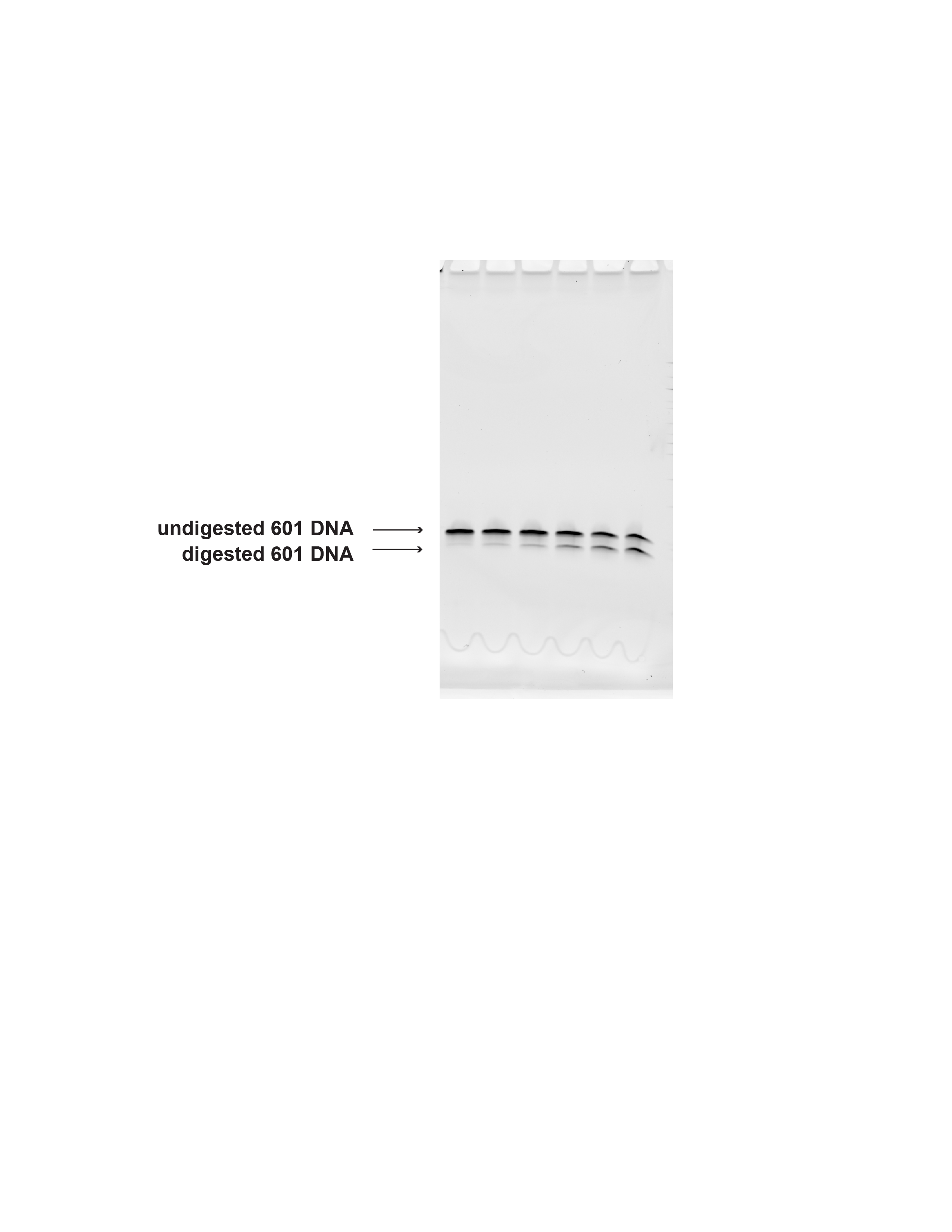

Supplement: Figure 7—figure supplement 1—source data 1. [file elife-71502-fig7-figsupp1-data1.zip › Figure 7-figure supplement 1B-source data 3.tif]

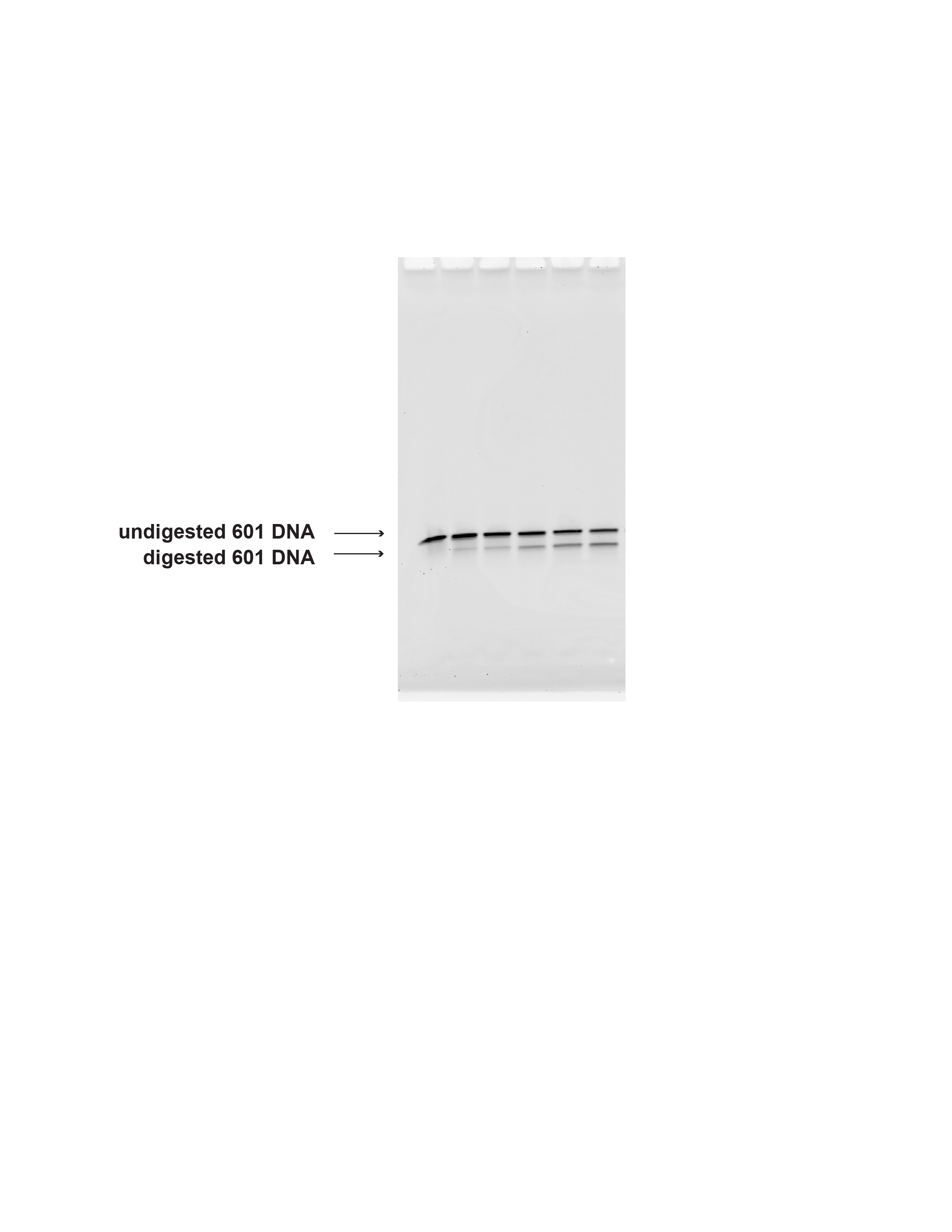

Supplement: Figure 7—figure supplement 1—source data 1. [file elife-71502-fig7-figsupp1-data1.zip › Figure 7-figure supplement 1B-source data 4.tif]

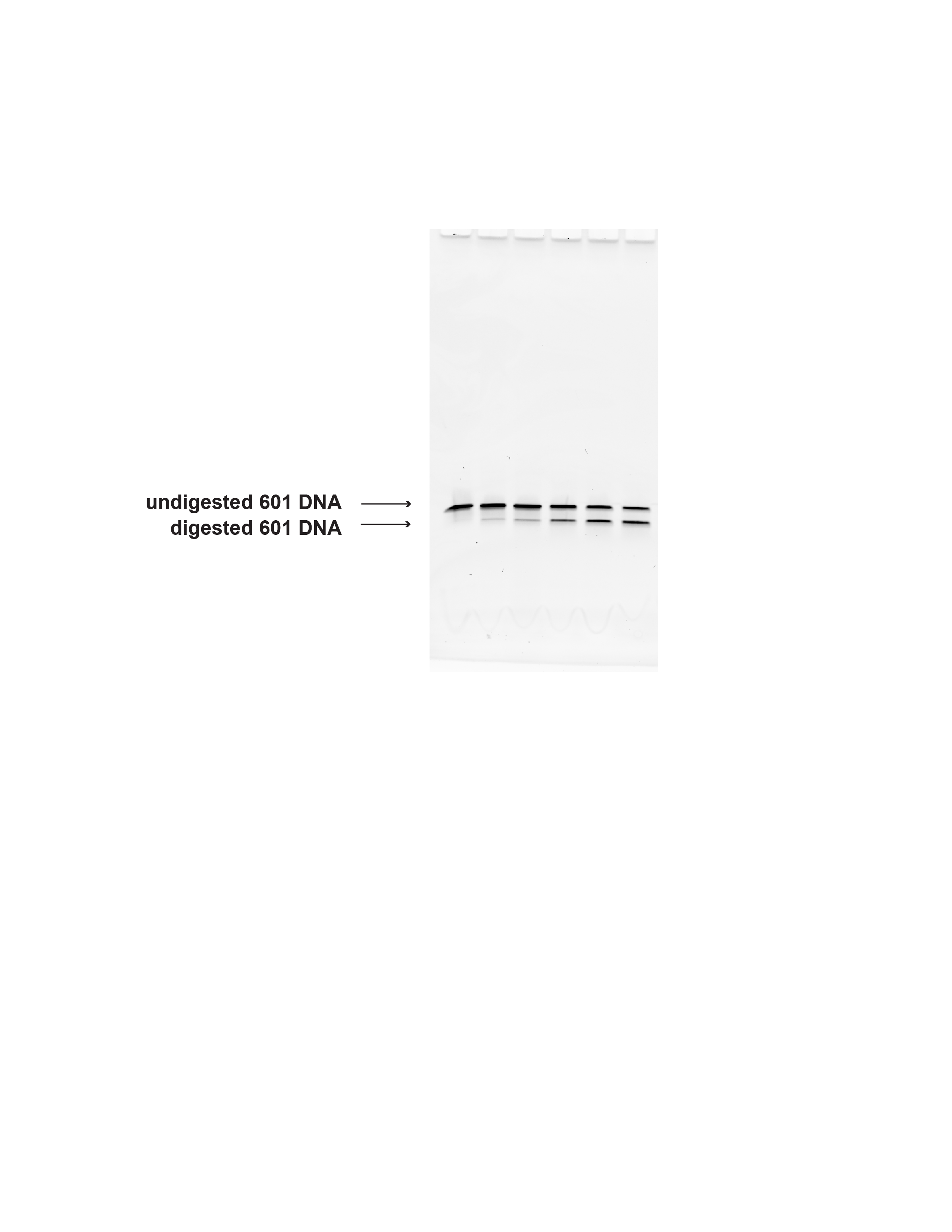

Supplement: Figure 7—figure supplement 1—source data 1. [file elife-71502-fig7-figsupp1-data1.zip › Figure 7-figure supplement 1B-source data 5.tif]

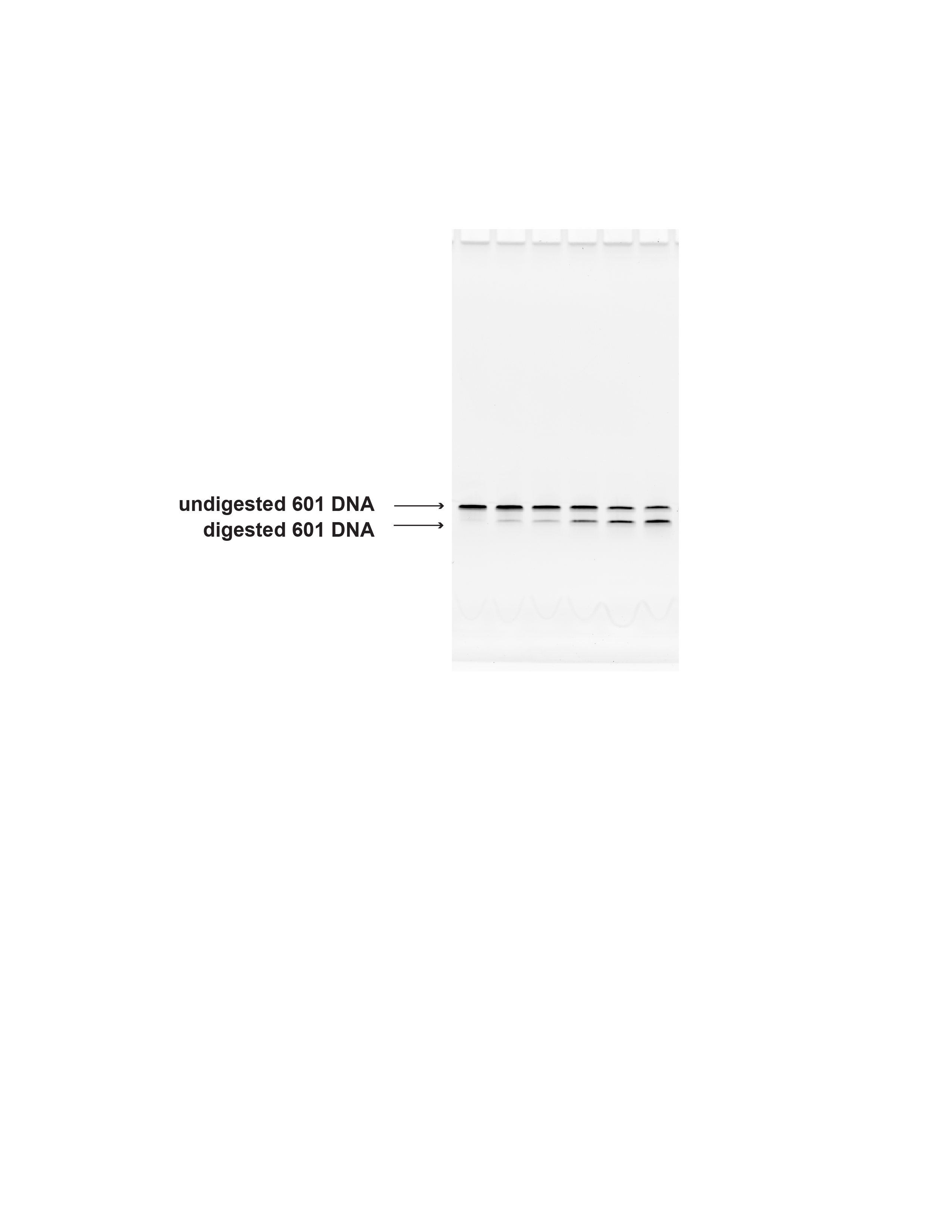

Supplement: Figure 7—figure supplement 1—source data 1. [file elife-71502-fig7-figsupp1-data1.zip › Figure 7-figure supplement 1B-source data 6.tif]

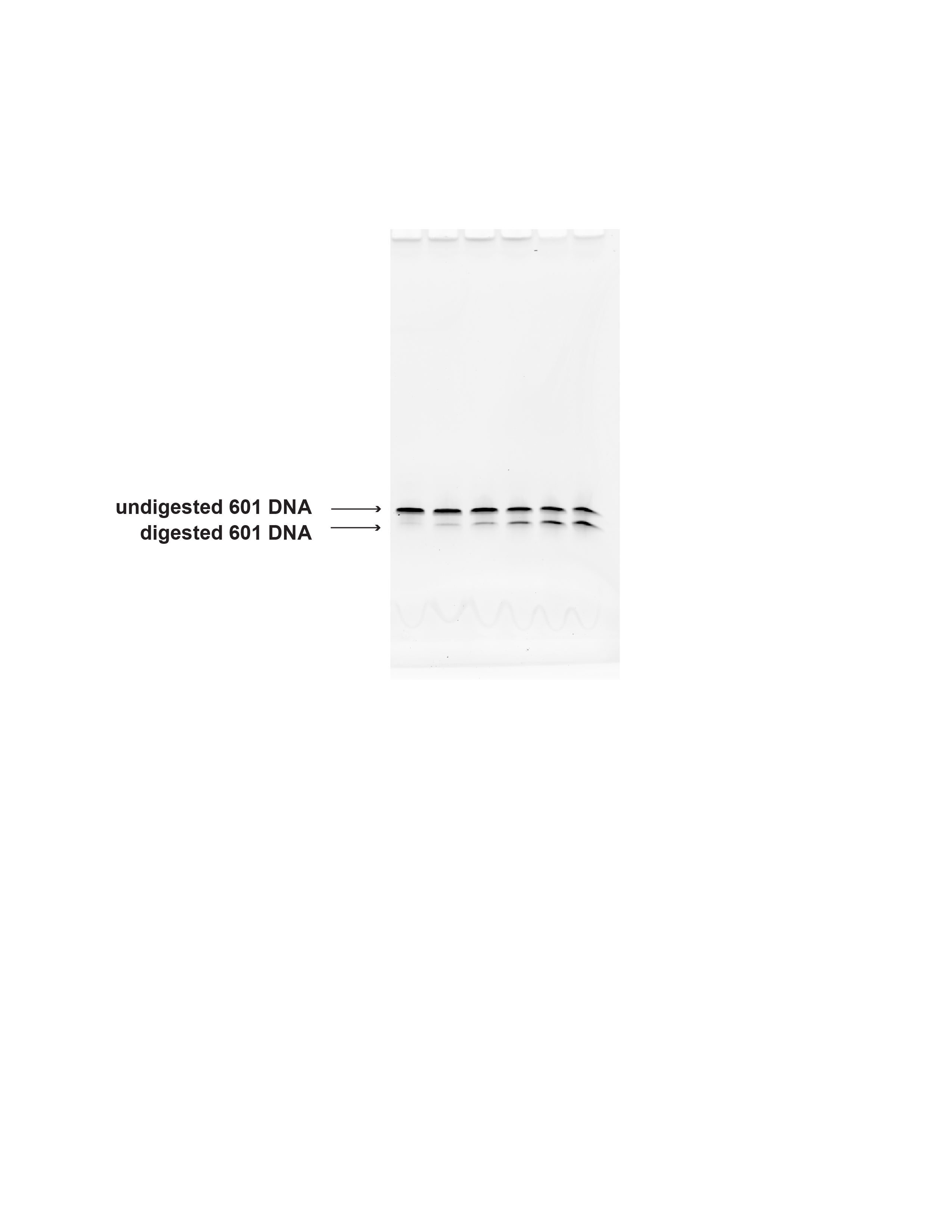

Supplement: Figure 7—figure supplement 1—source data 1. [file elife-71502-fig7-figsupp1-data1.zip › Figure 7-figure supplement 1B-source data 7.tif]

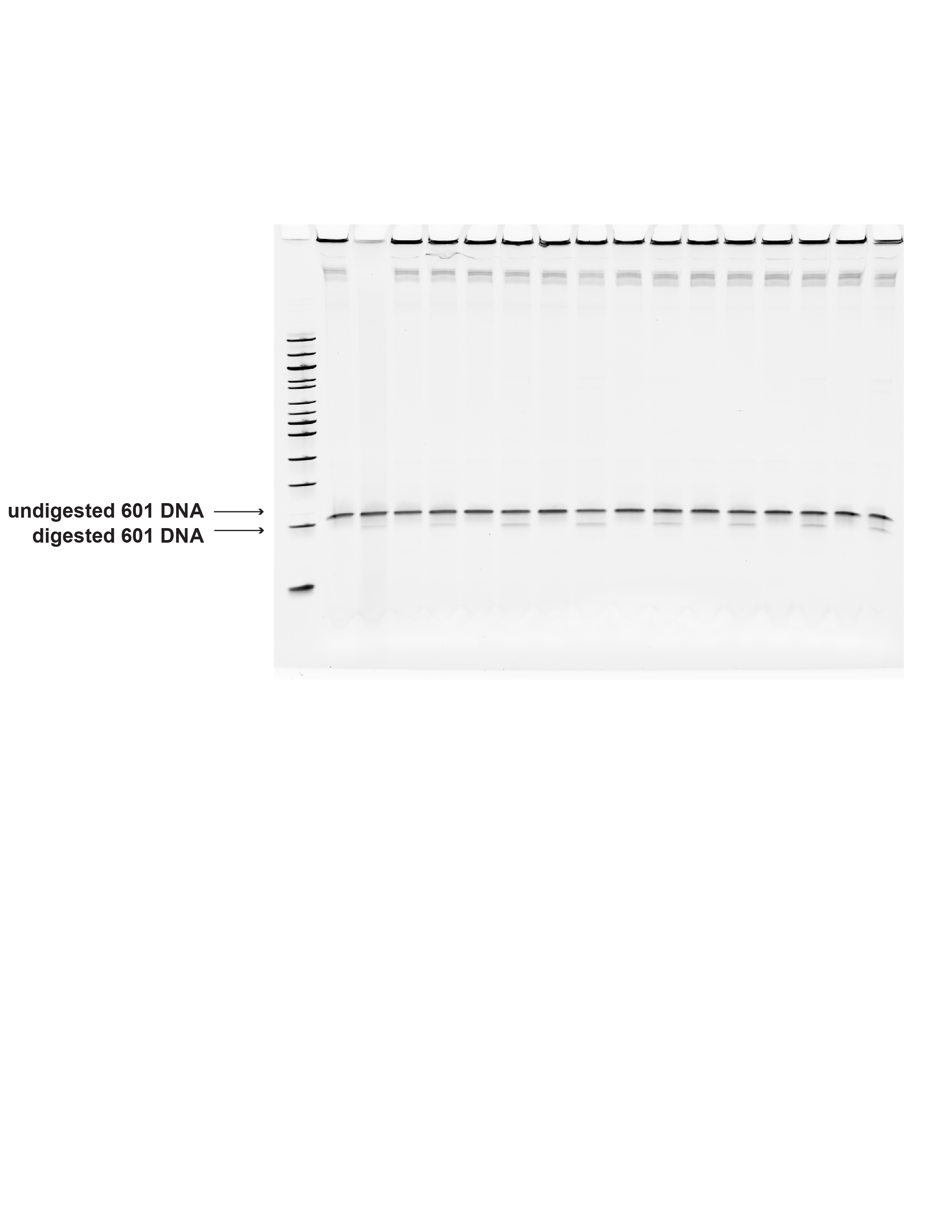

Supplement: Figure 7—figure supplement 1—source data 1. [file elife-71502-fig7-figsupp1-data1.zip › Figure 7-figure supplement 1C-source data 1.tif]

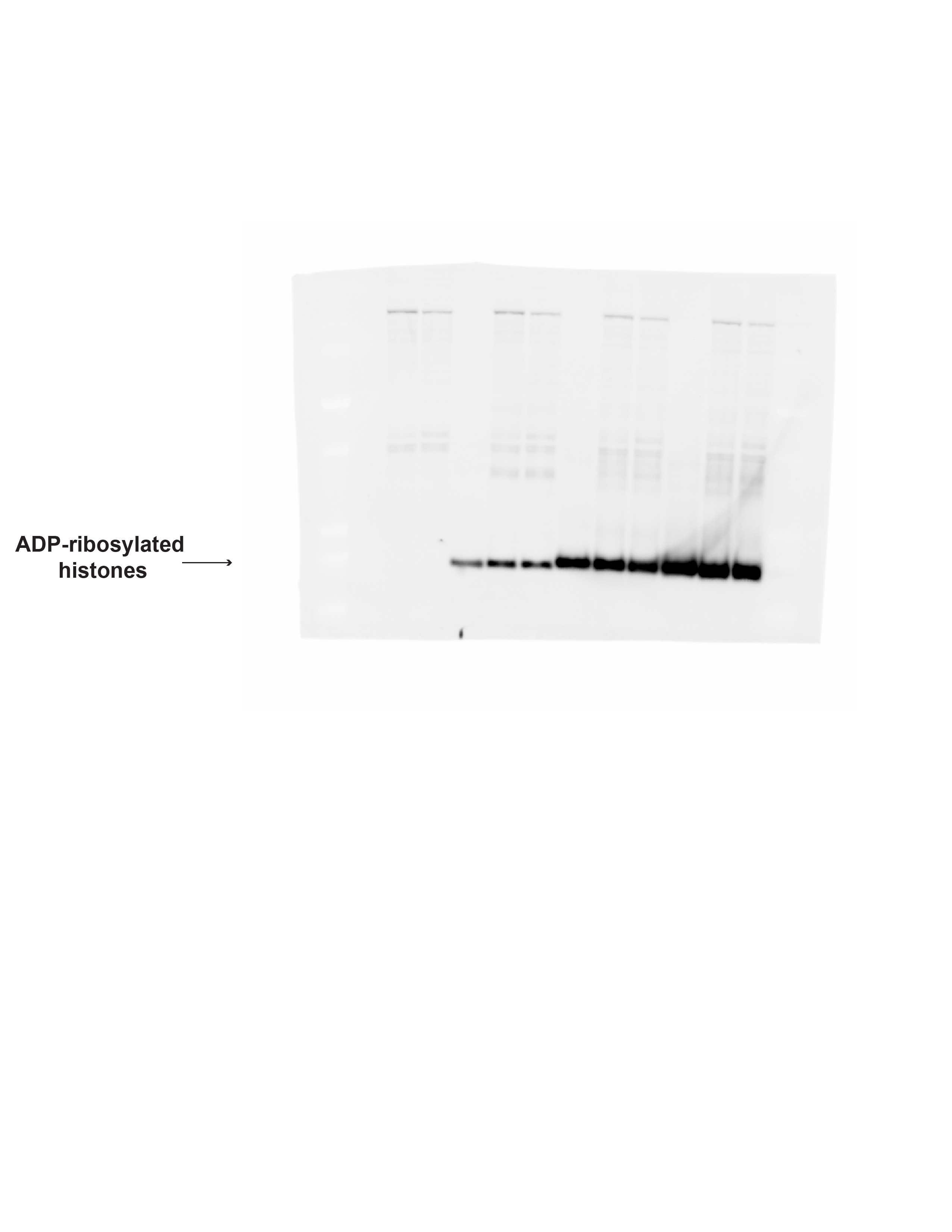

Supplement: Figure 7—figure supplement 1—source data 1. [file elife-71502-fig7-figsupp1-data1.zip › Figure 7-figure supplement 1D-source data 1.tif]

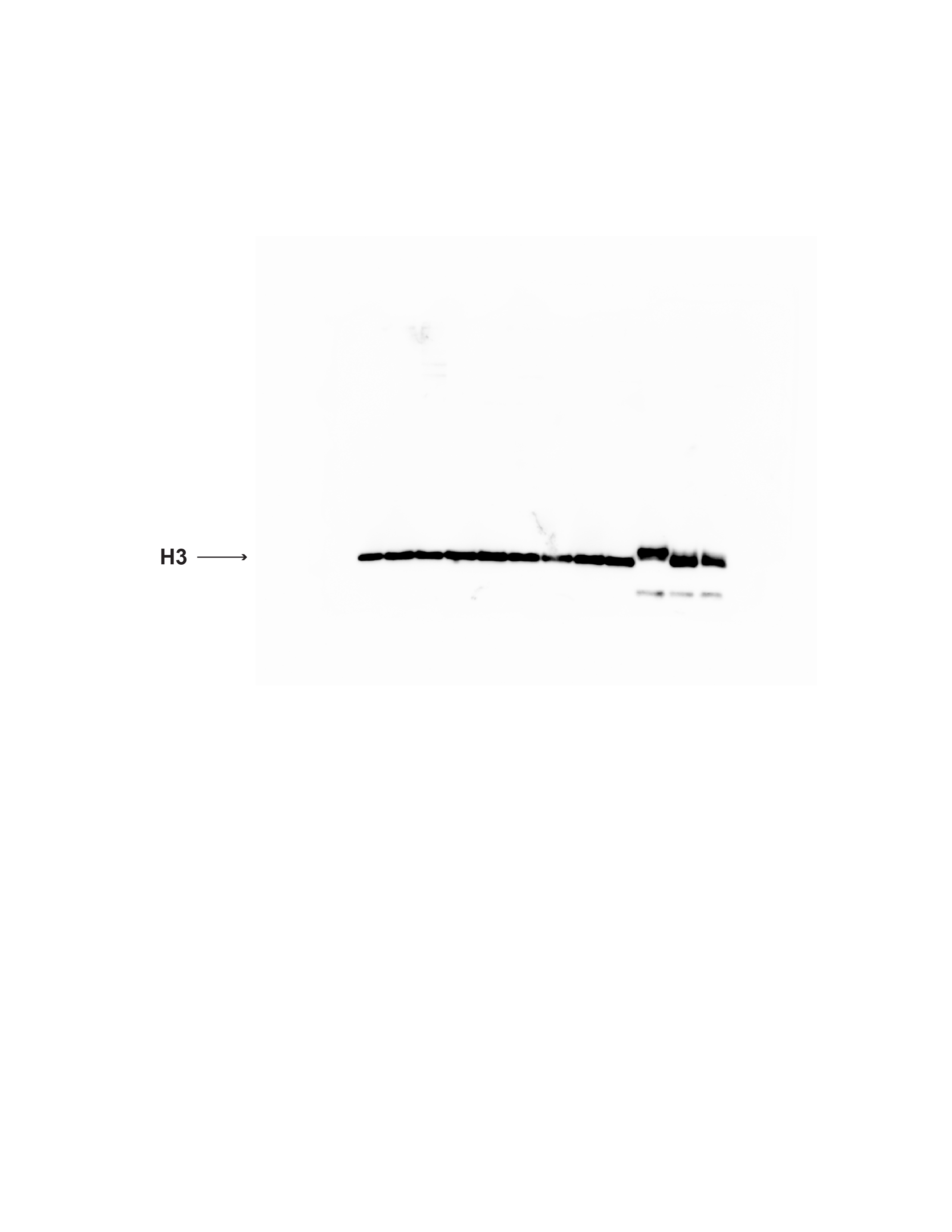

Supplement: Figure 7—figure supplement 1—source data 1. [file elife-71502-fig7-figsupp1-data1.zip › Figure 7-figure supplement 1D-source data 2.tif]

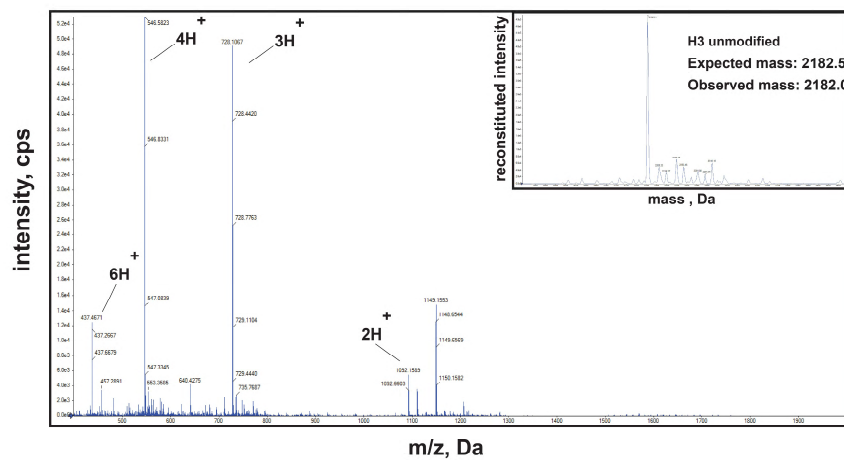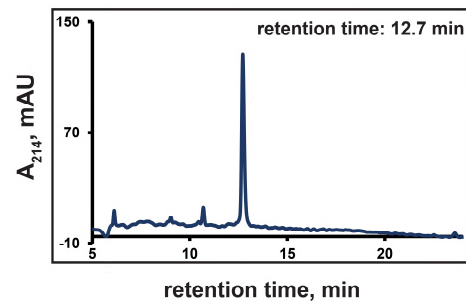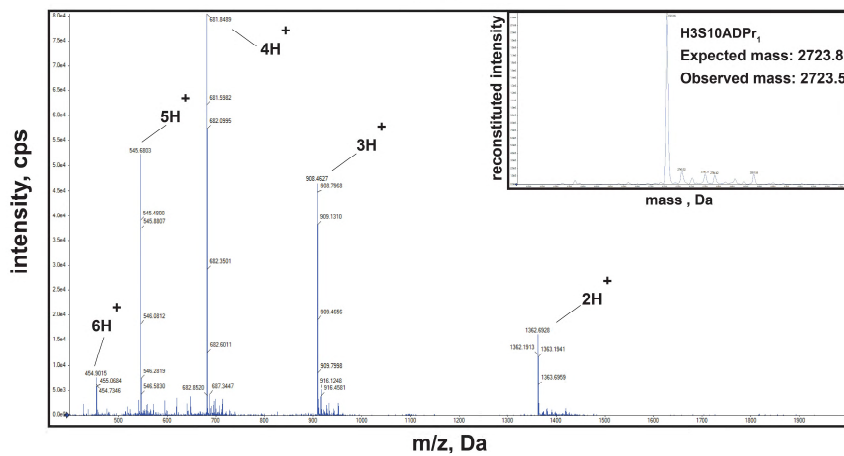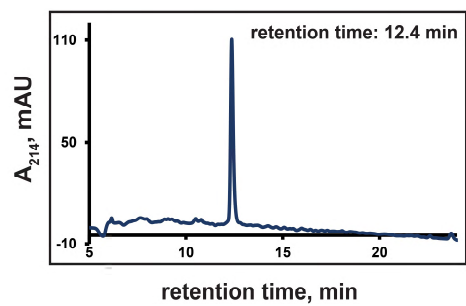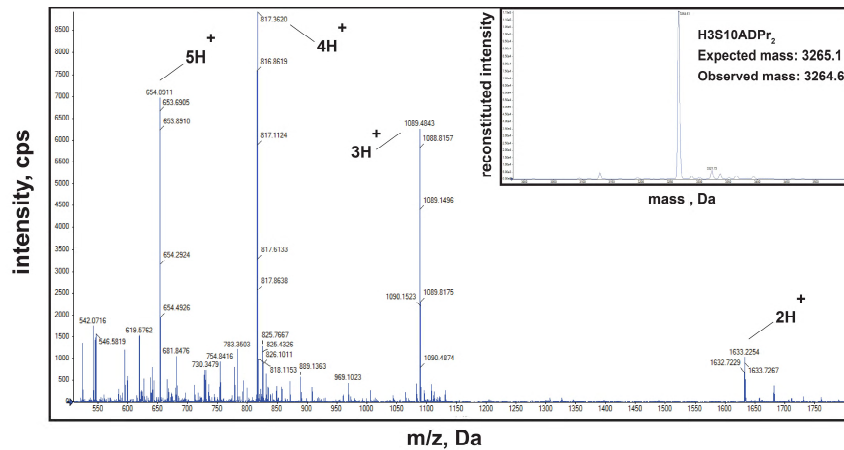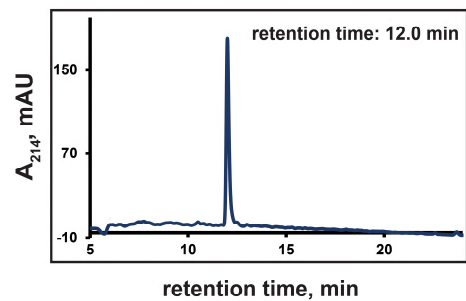

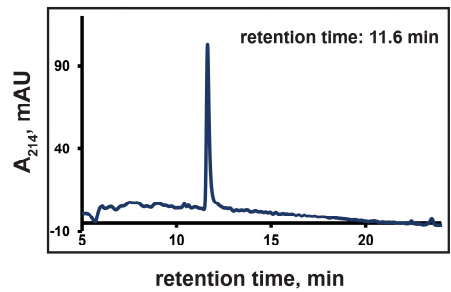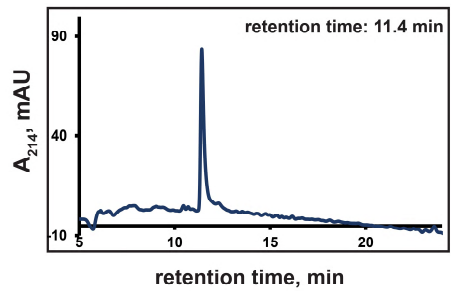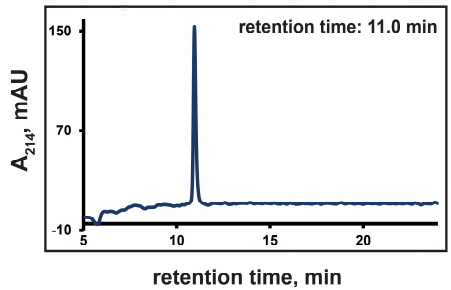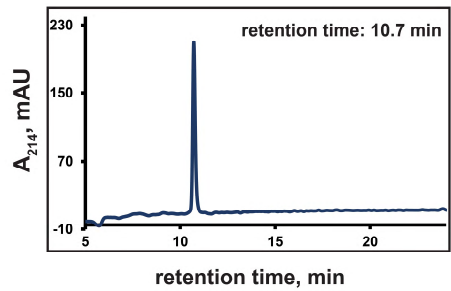

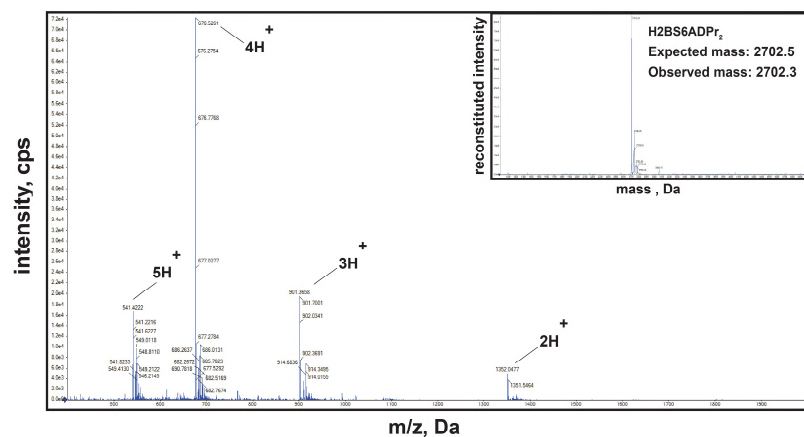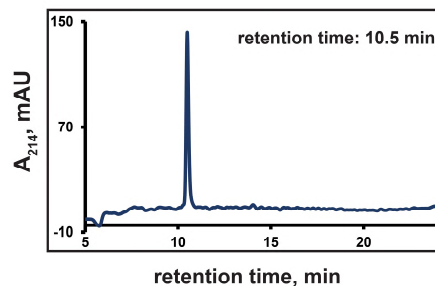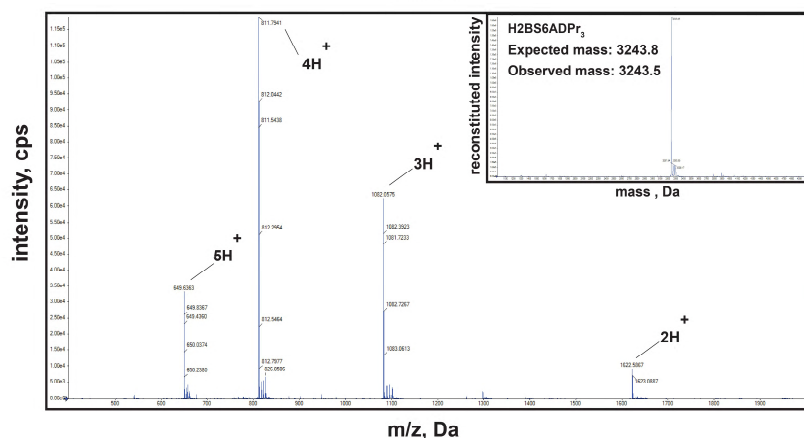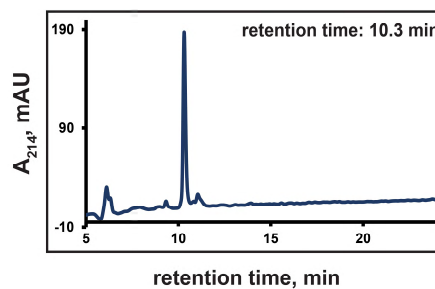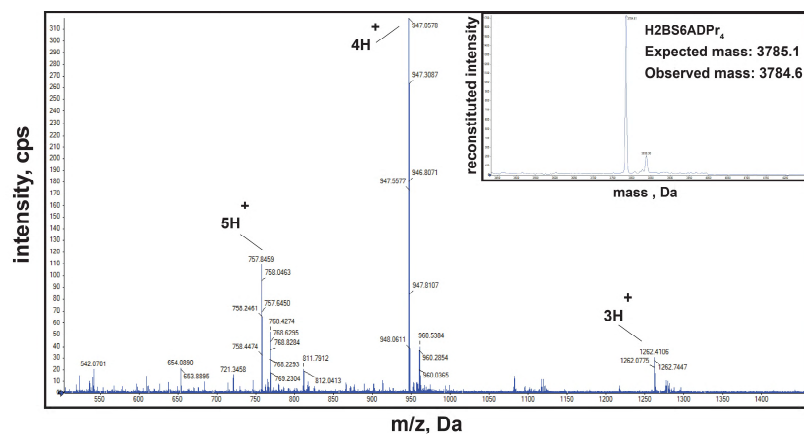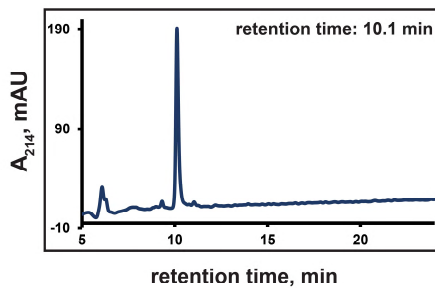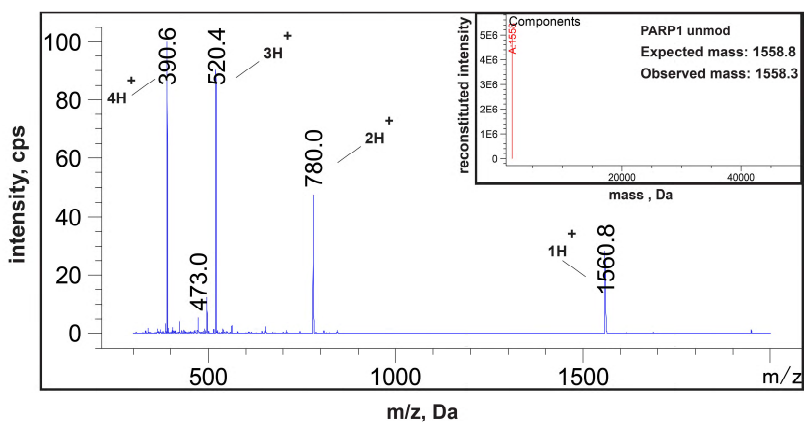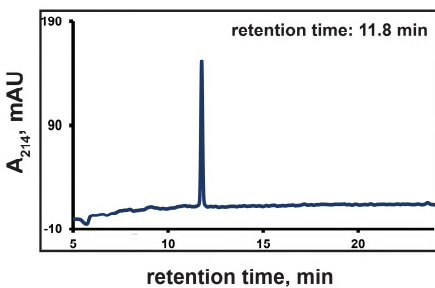

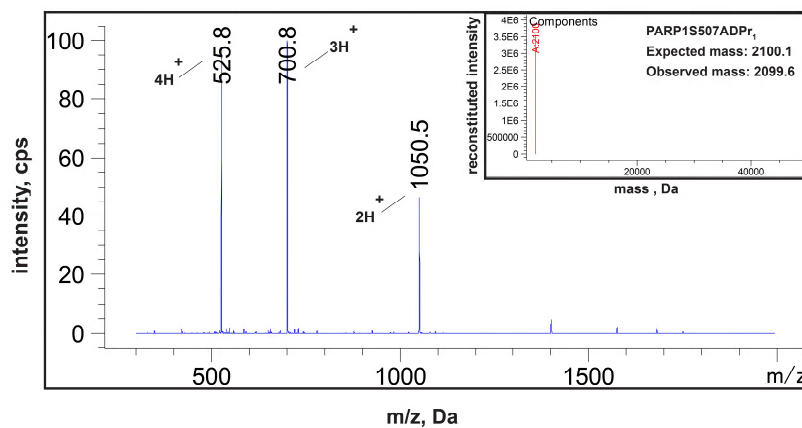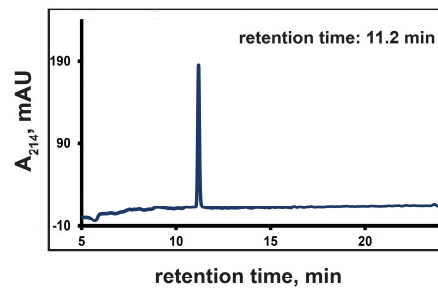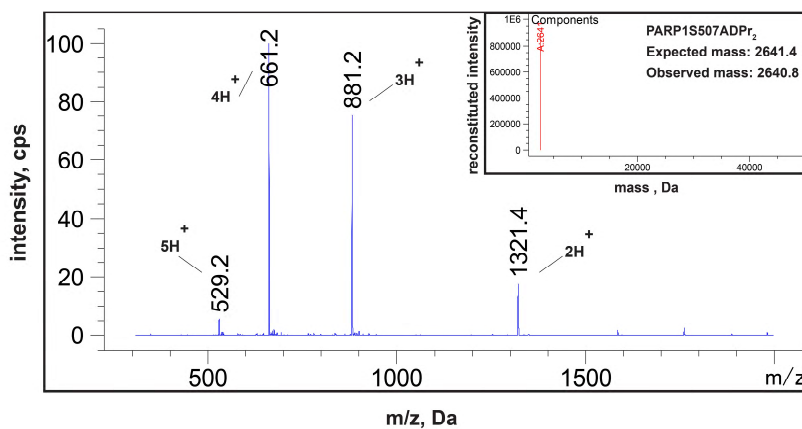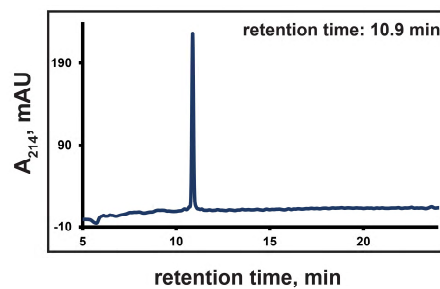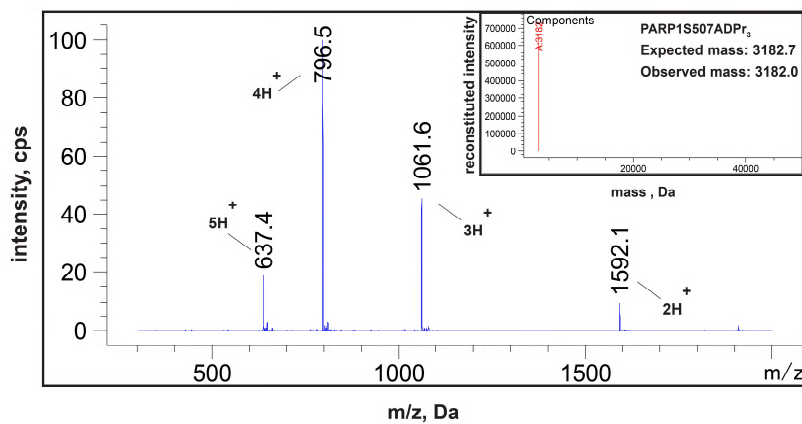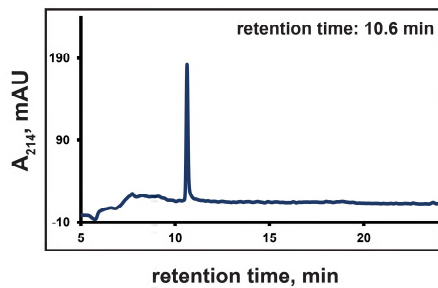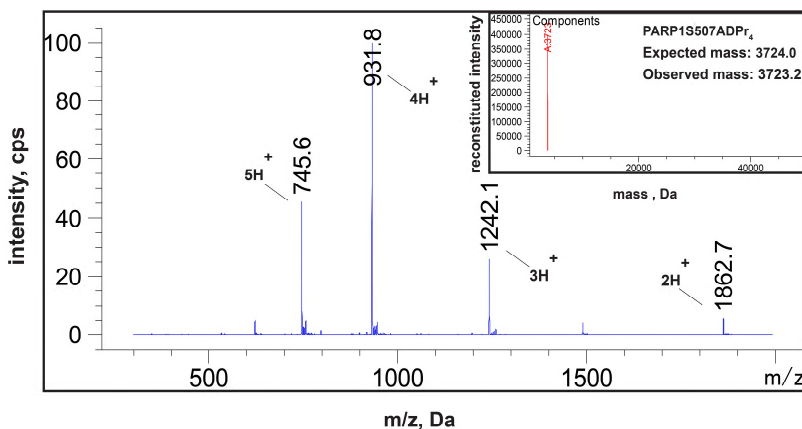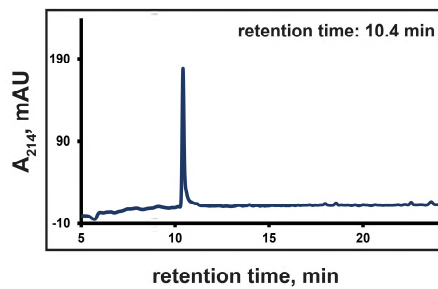

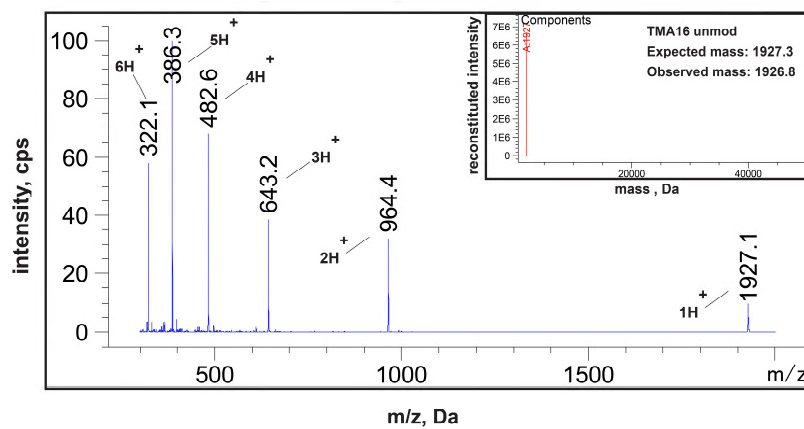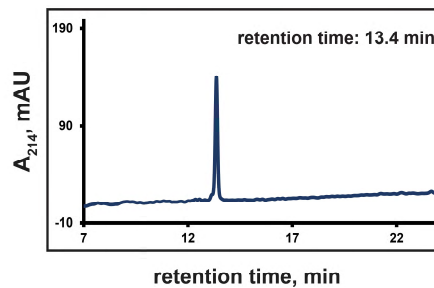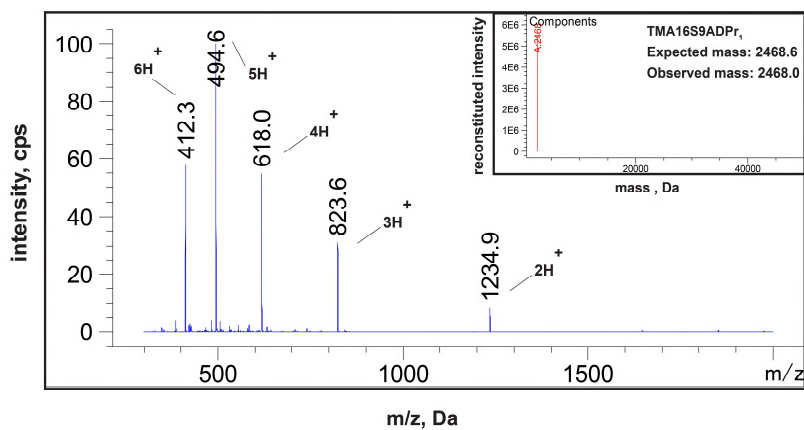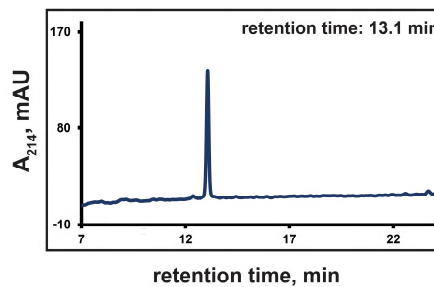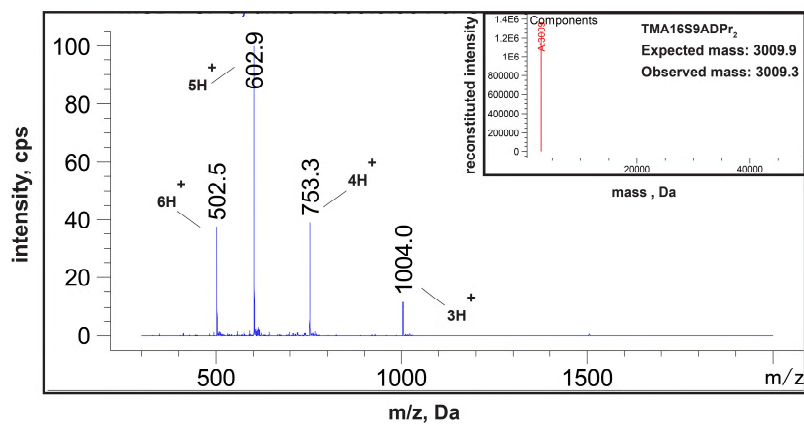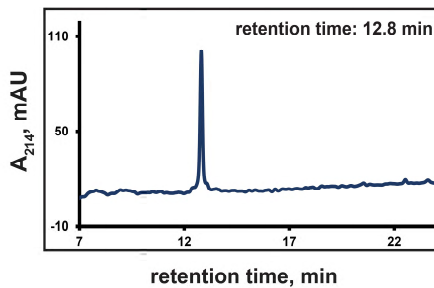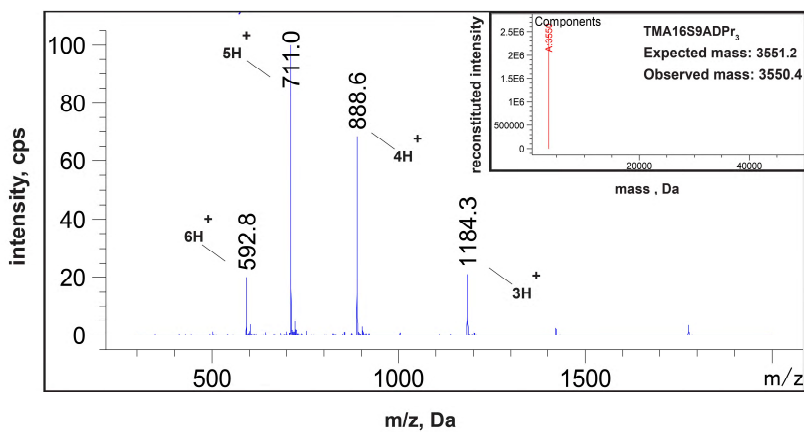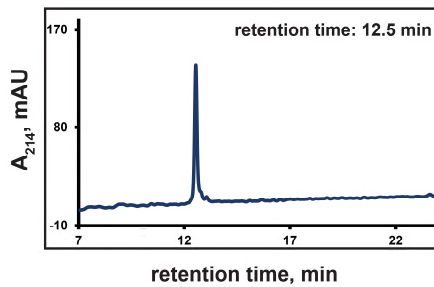

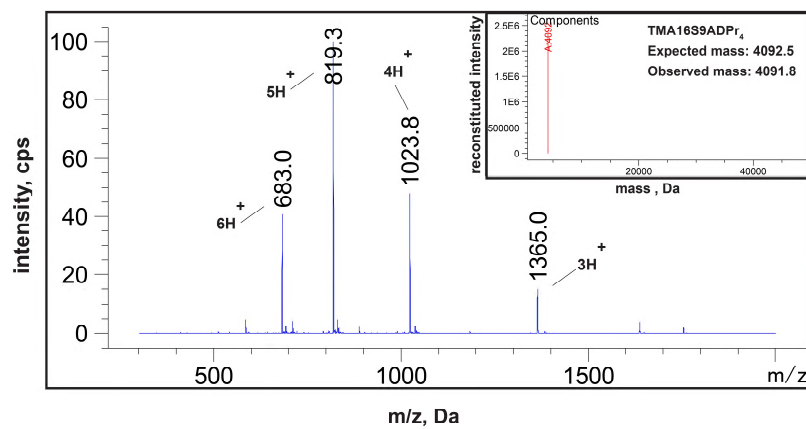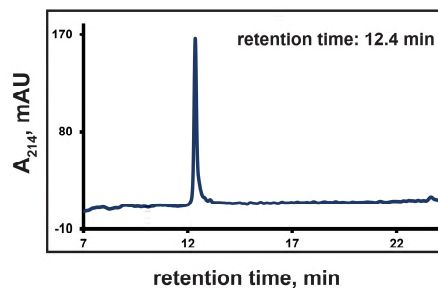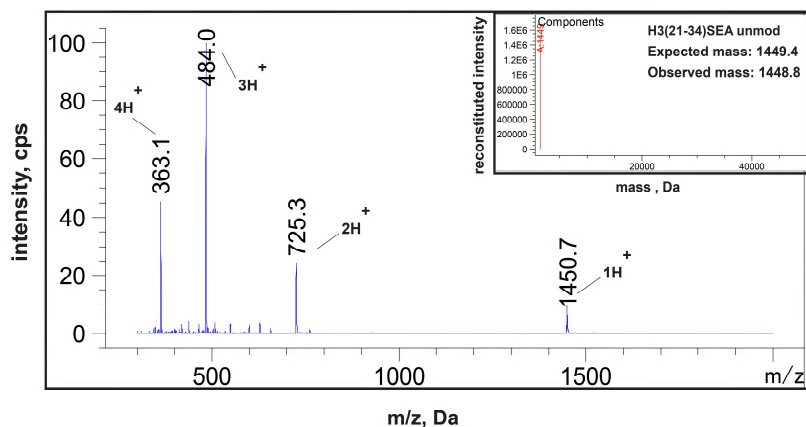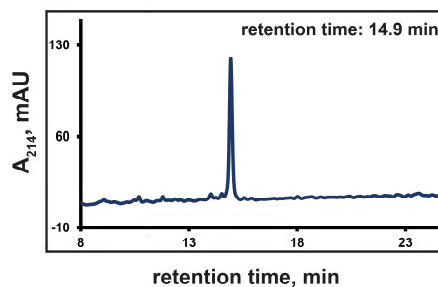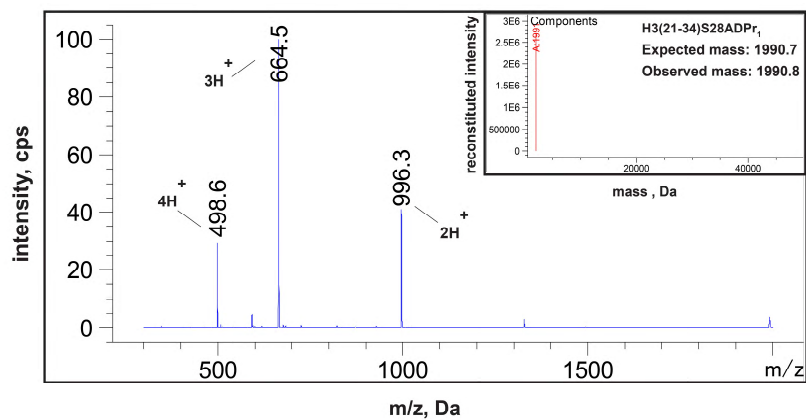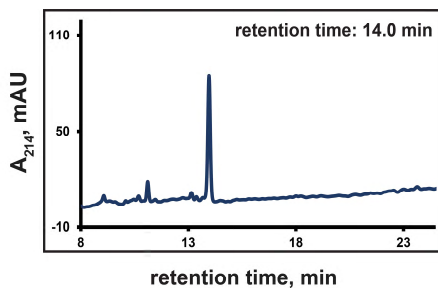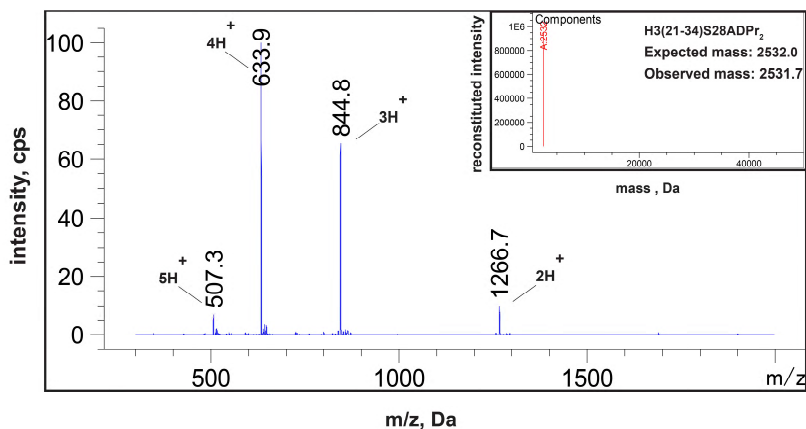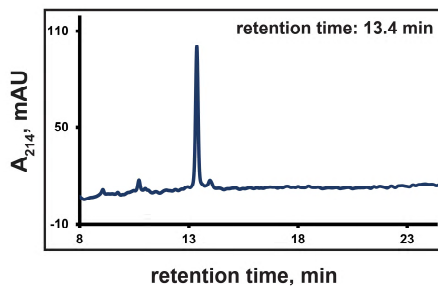

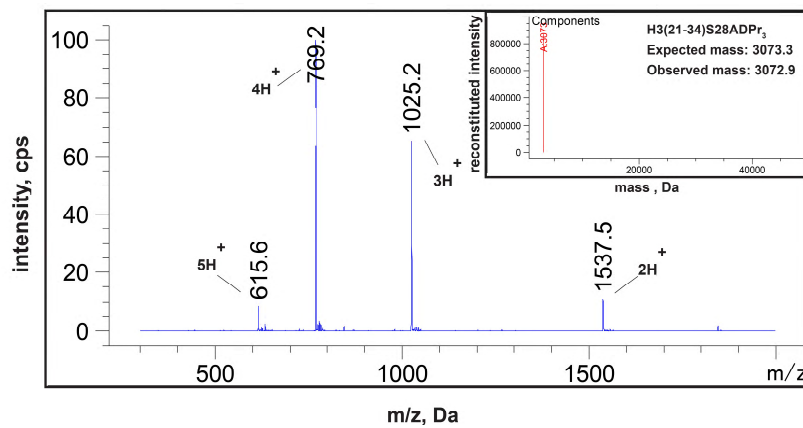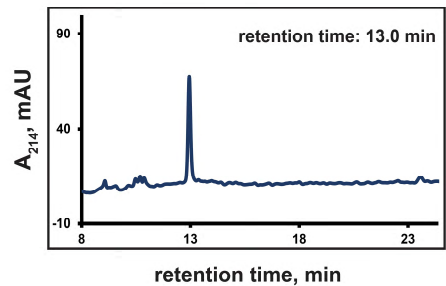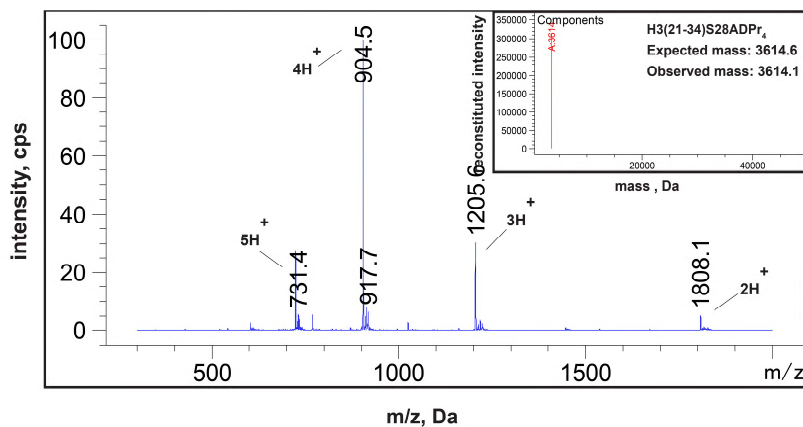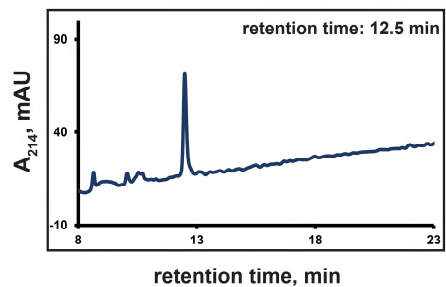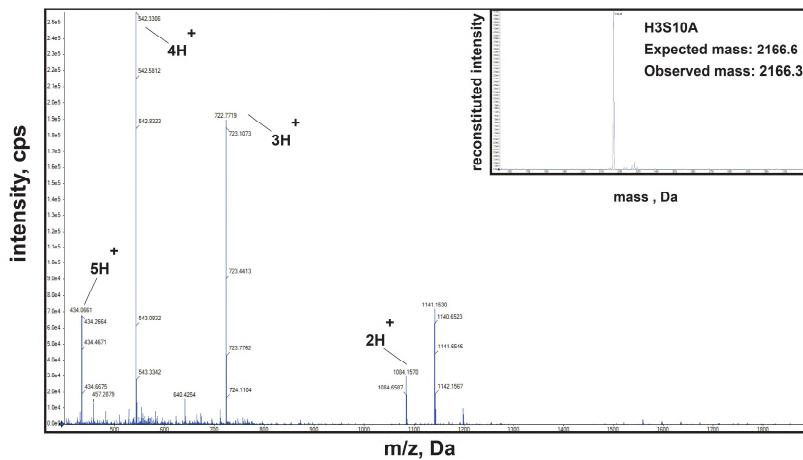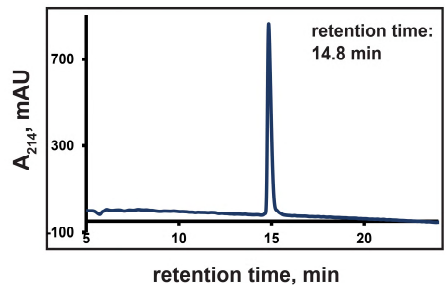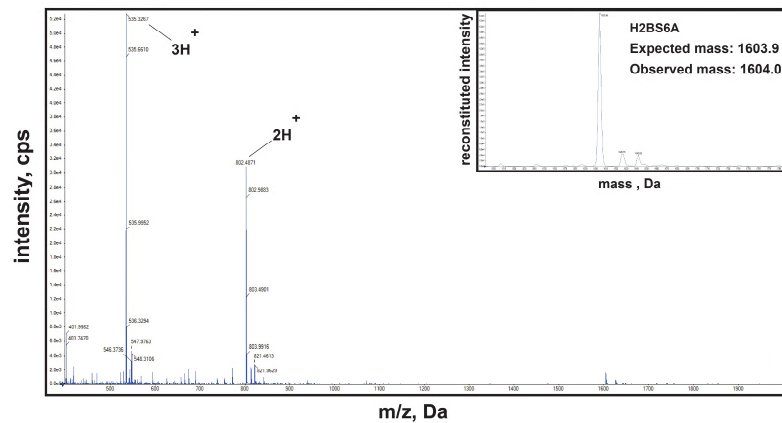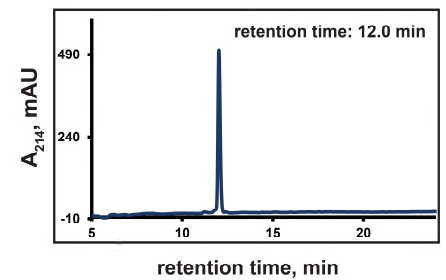

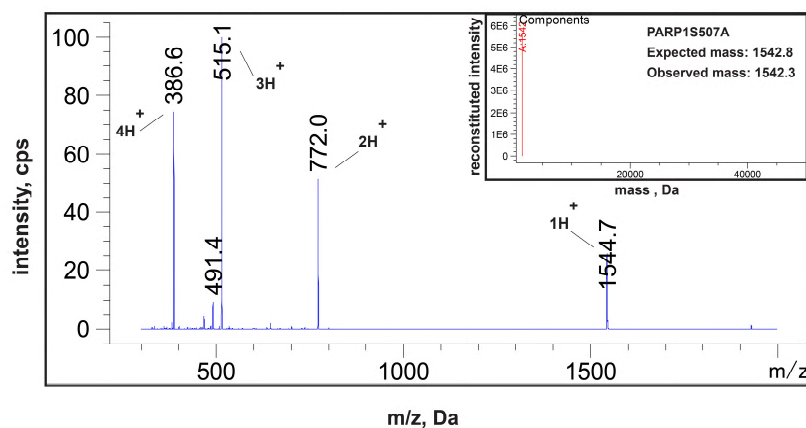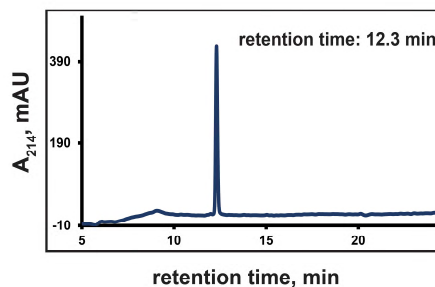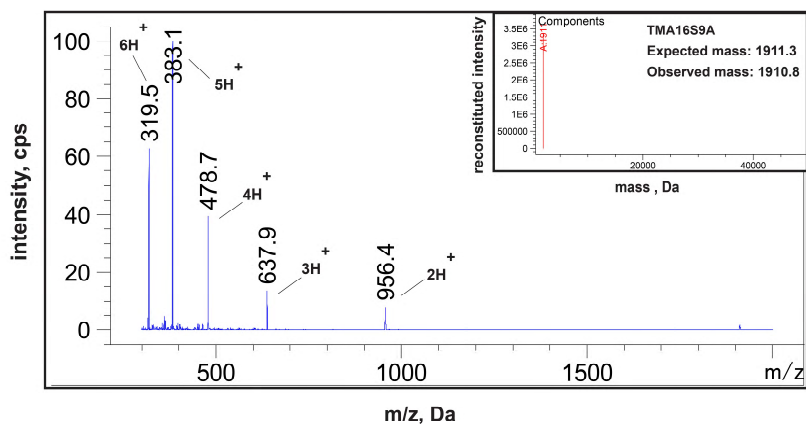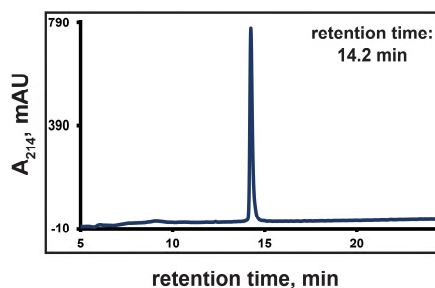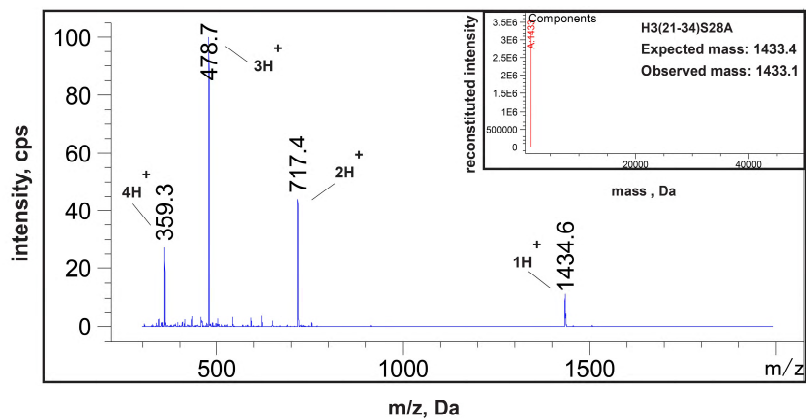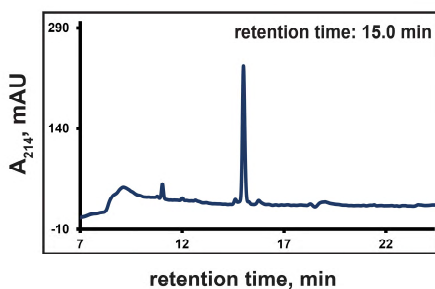

Supplement: Supplementary file 1. [file elife-71502-supp1.pdf]

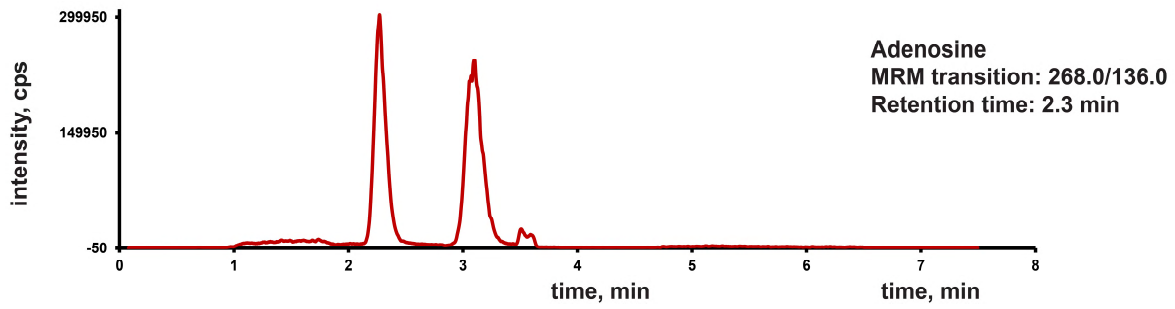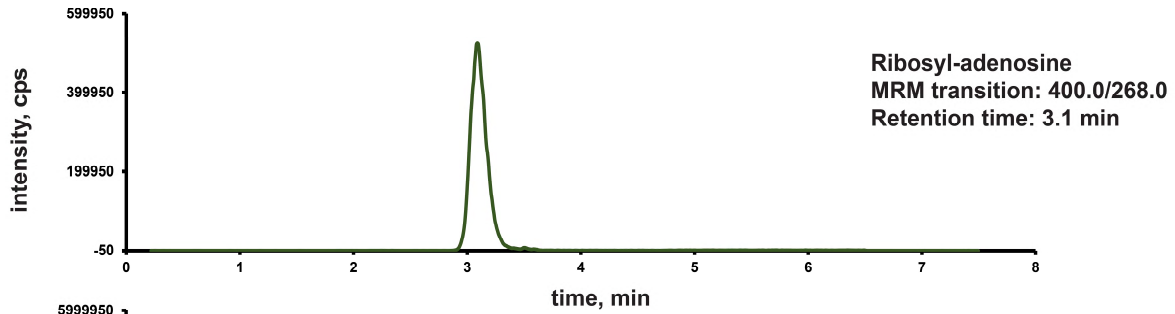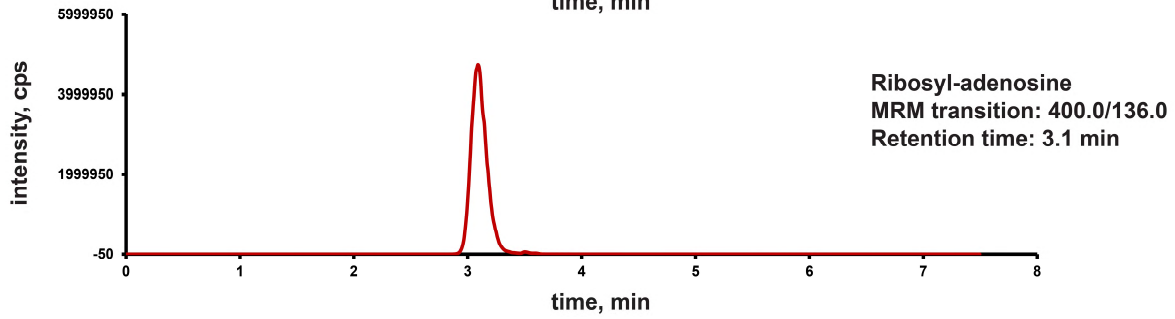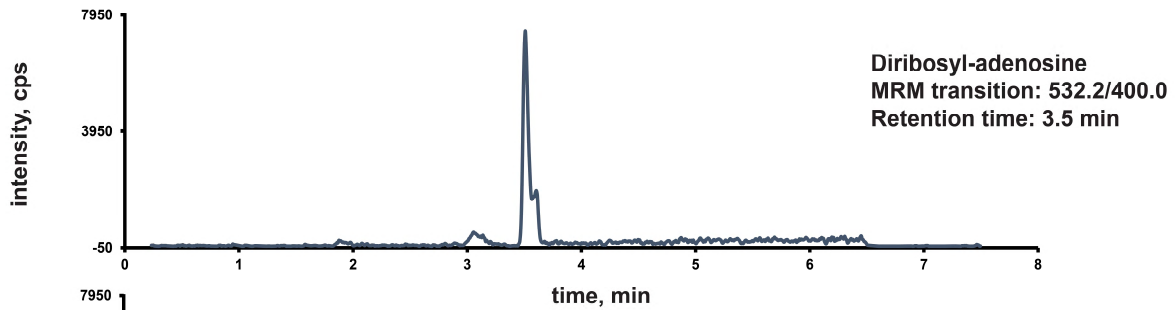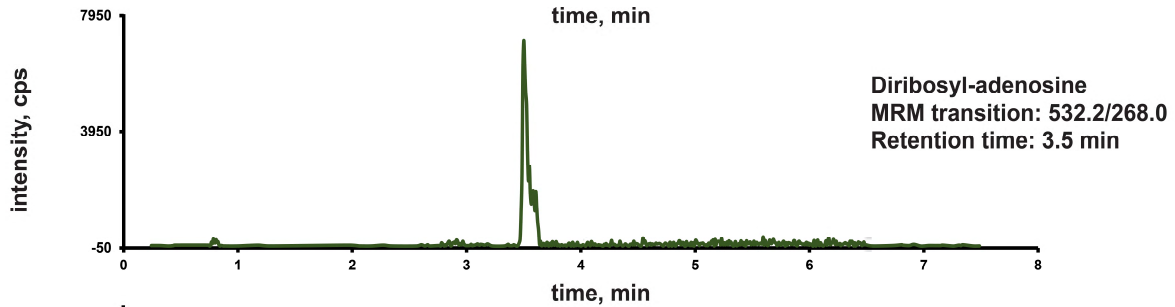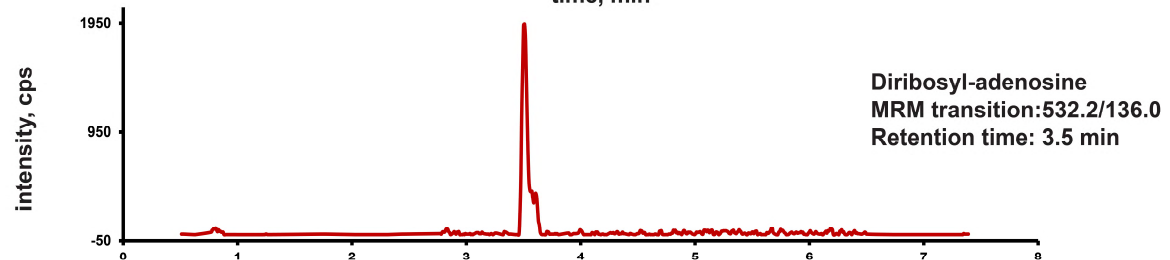

Supplement: Supplementary file 2. — The spectra were produced on a Sciex QTRAP 6500+ mass spectrometer. [file elife-71502-supp2.zip › Supplementary_File_2.pdf]

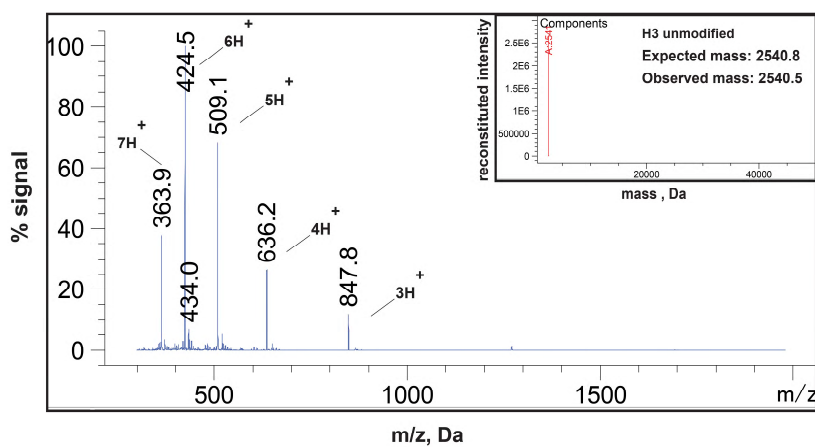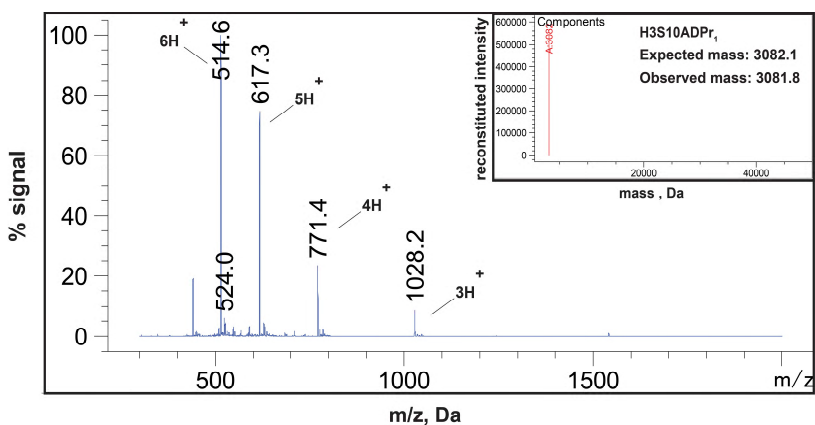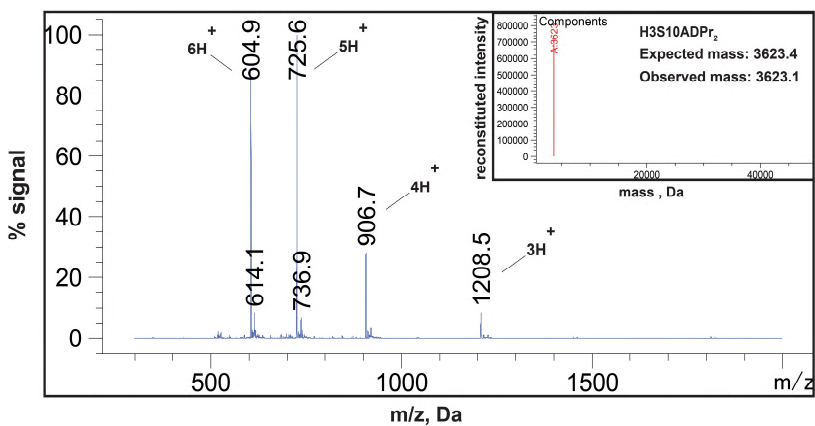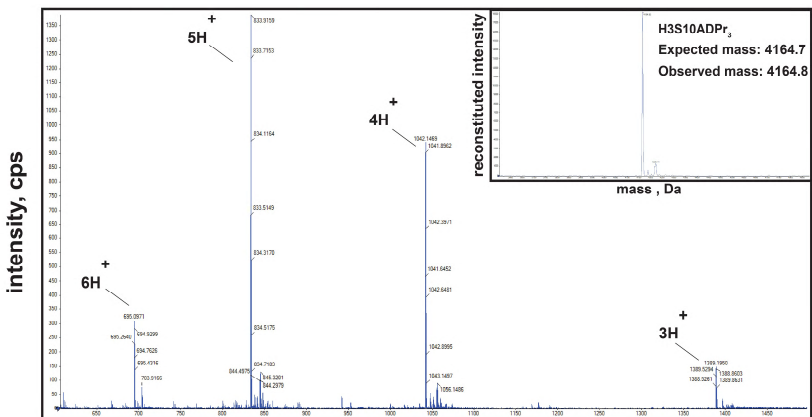

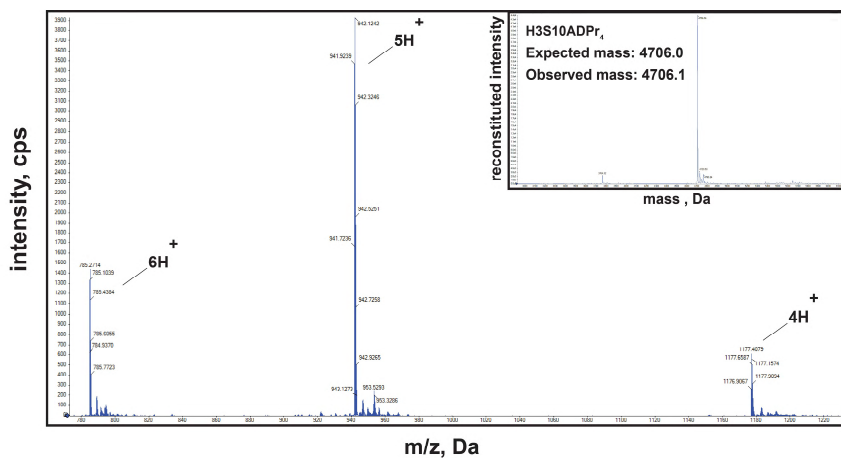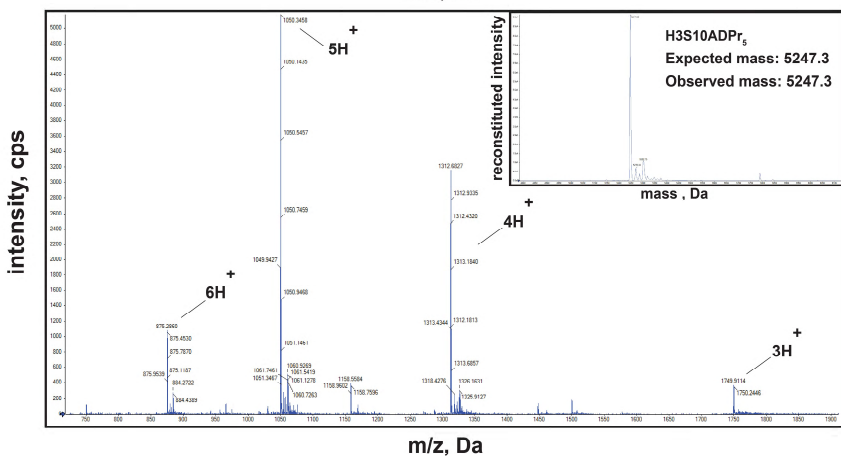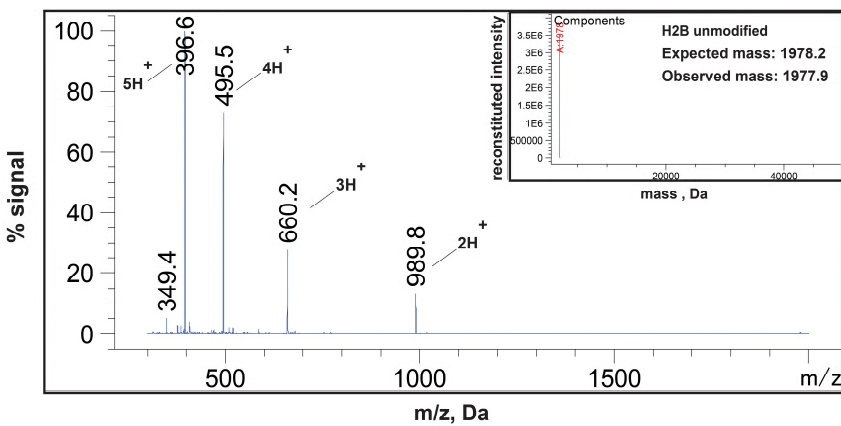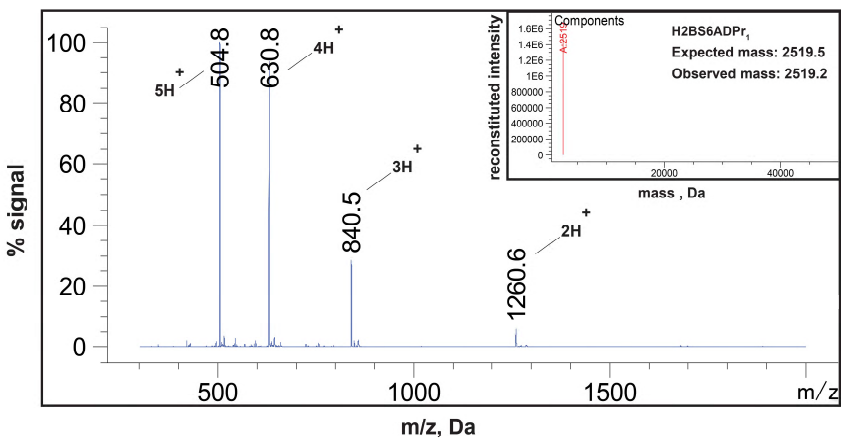

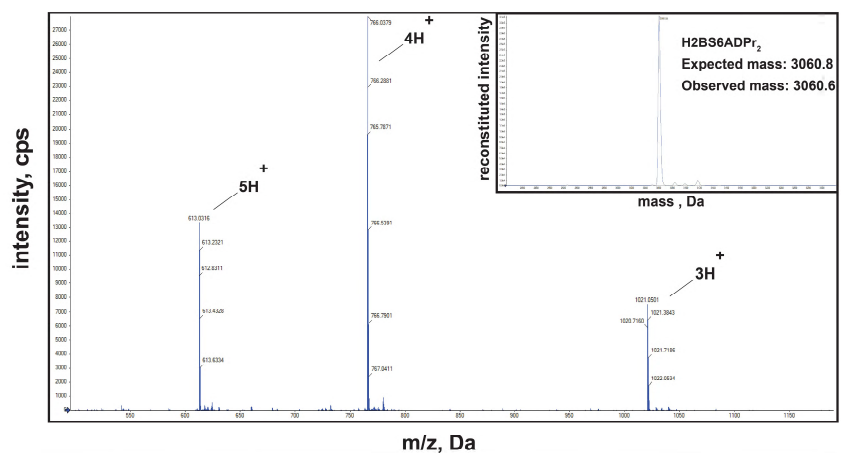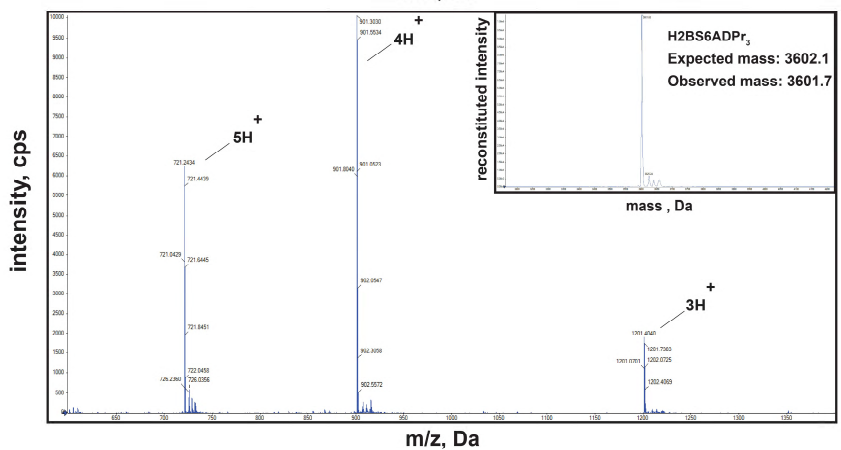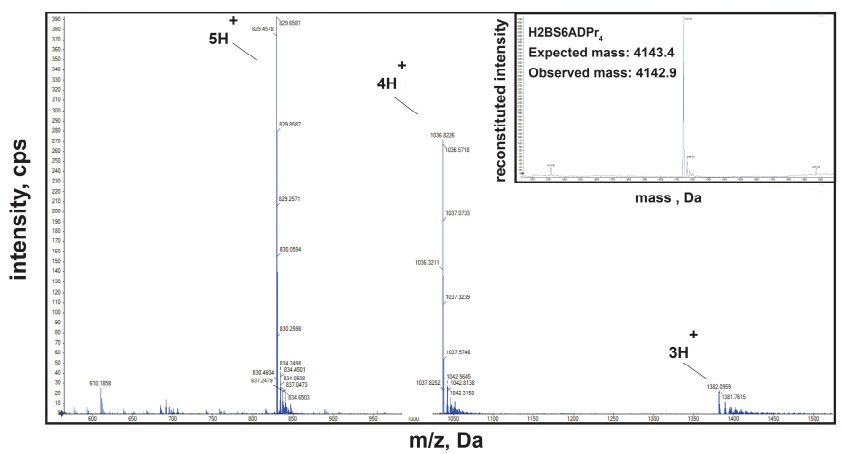

Supplement: Supplementary file 3. [file elife-71502-supp3.pdf]

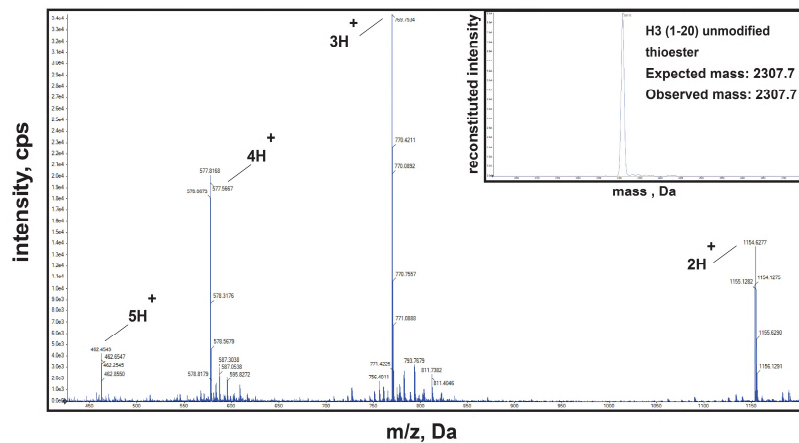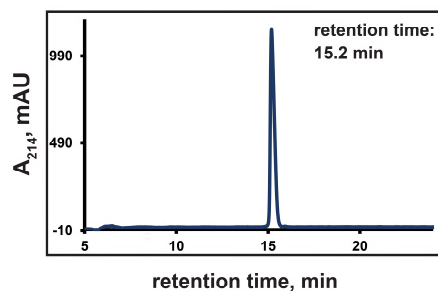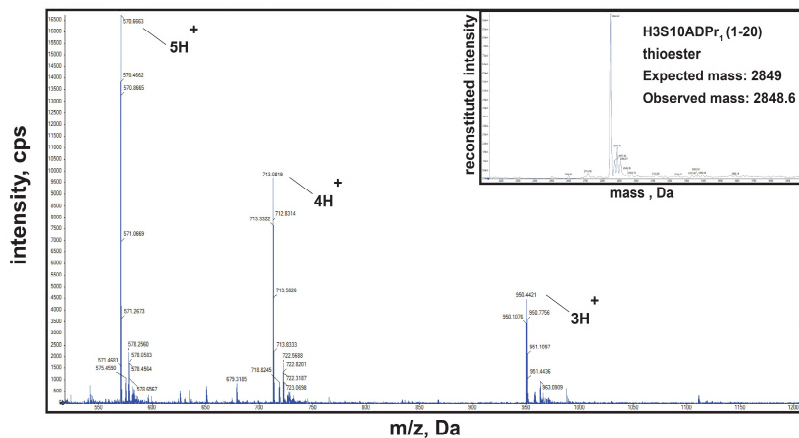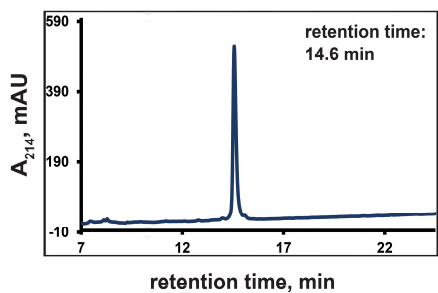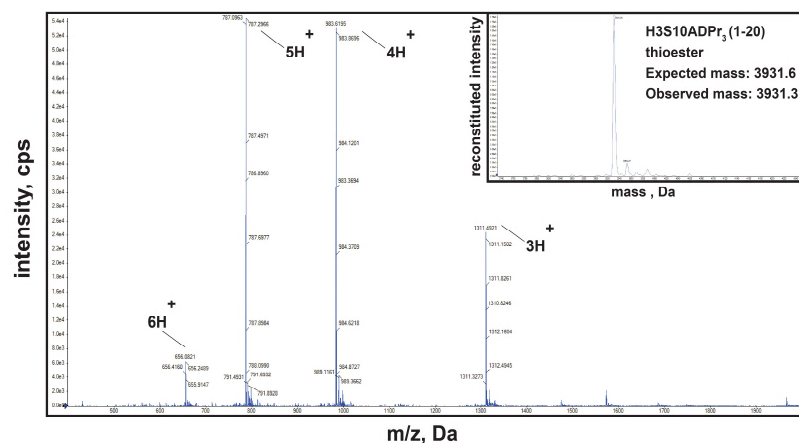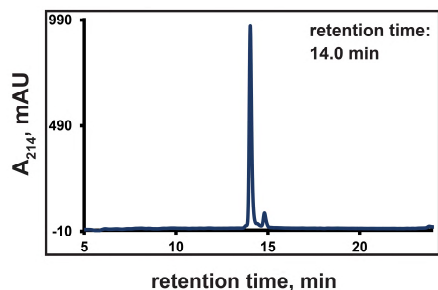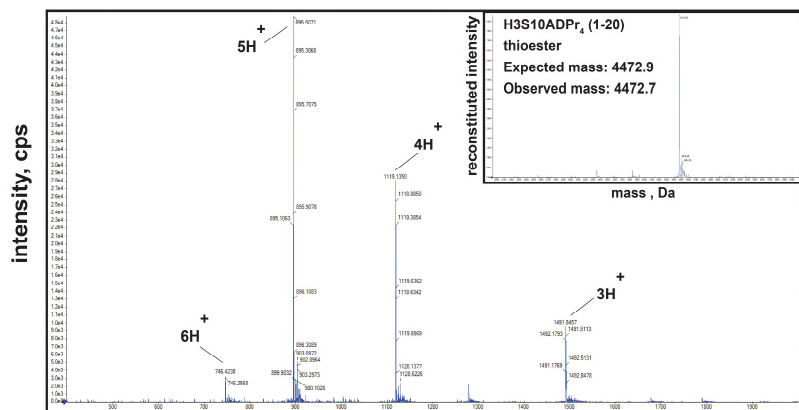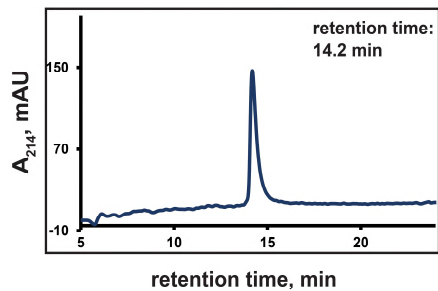

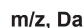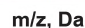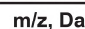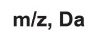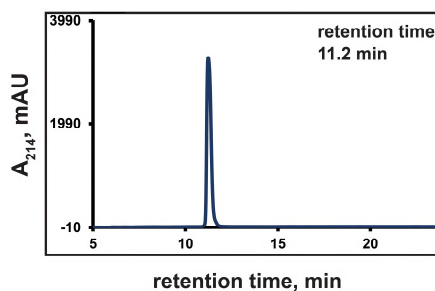

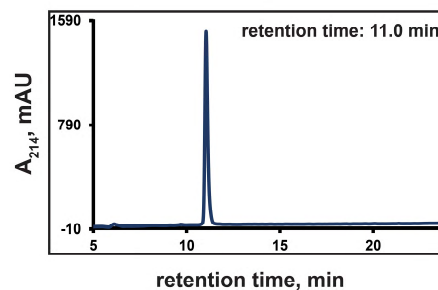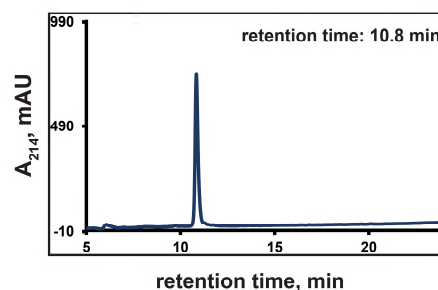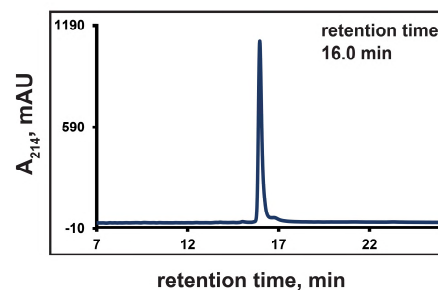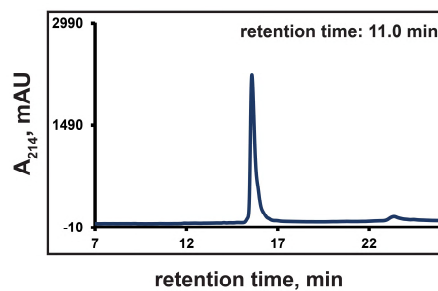

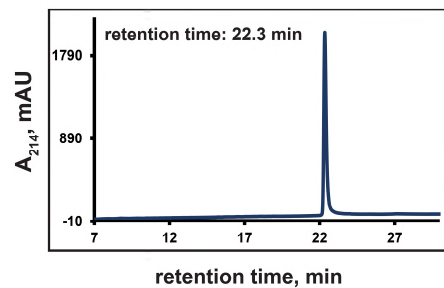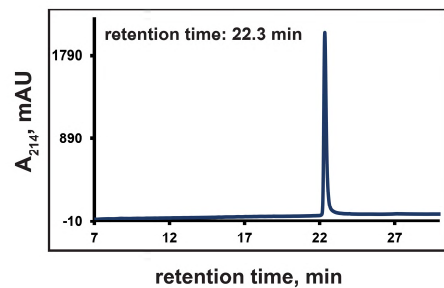

Supplement: Supplementary file 5. [file elife-71502-supp5.pdf]

**A**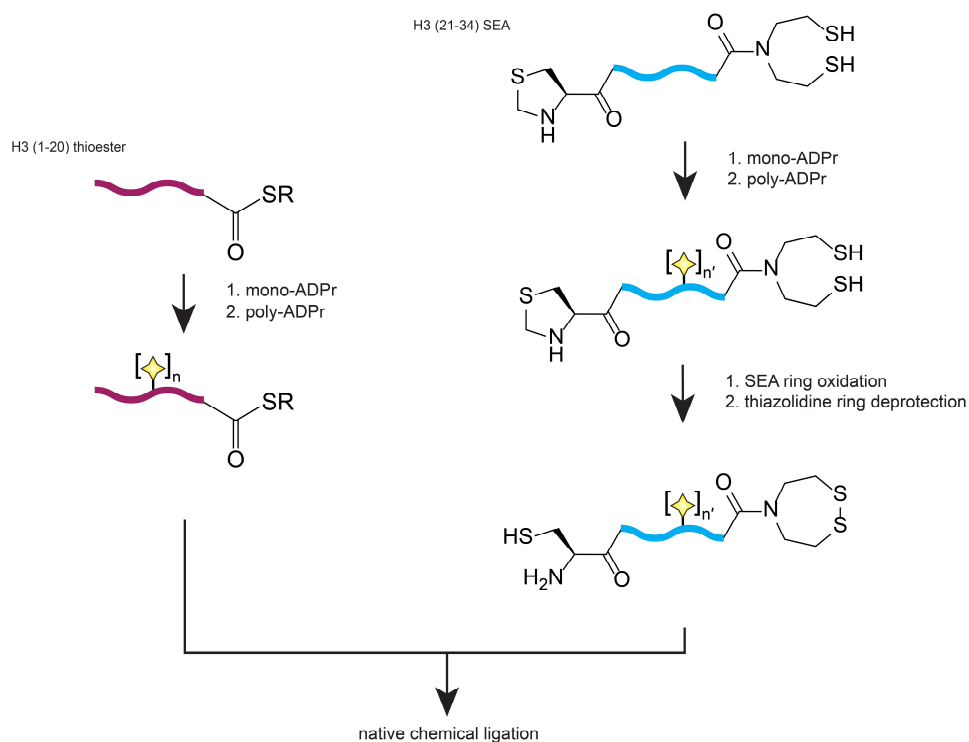**B**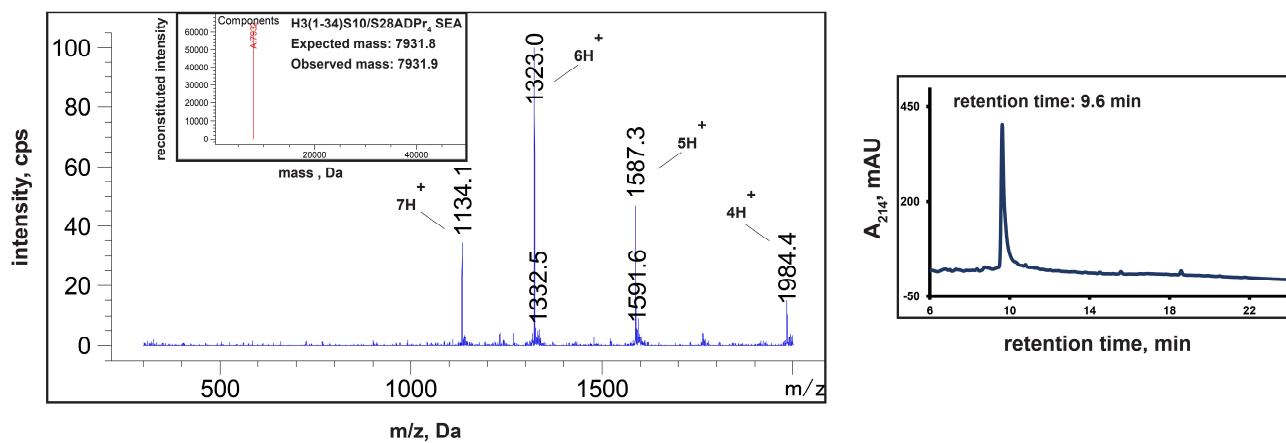

Supplement: Supplementary file 9. — A, A schematic depicting the strategy for C-terminal ligation of ADP-ribosylated peptides. SEA = bis(2-sulfanylethyl)amido functional group. B, ESI-MS and RP-HPLC characterization of a C-terminal ligation product which has tetra-ADP-ribose on S10 and S28 residues of the H3 peptide (amino acids 1–34), as well as a reduced C-terminal SEA moiety. RP-HPLC gradient is from 0%–80% Solvent B (2–22 min). The yellow star represents ADP-ribose modification. ‘n’ and ‘n׳’ represent the number of ADP-ribose units on the corresponding modification sites. [file elife-71502-supp9.pdf]
