# Supplementary material for: Serine ADP-ribosylation marks nucleosomes for ALC1-dependent chromatin remodeling
Source: eLife. 2021 Dec 7;10:e71502. doi: 10.7554/eLife.71502 (PMC8683085; doi:10.7554/eLife.71502)
Supplement: Supplementary file 4. [file elife-71502-supp4.docx]

| **Peptide** | **K_d,app_ (nM)** | **95% CI** | **R^2^** |
| --- | --- | --- | --- |
| H3 unmodified | n.d. | n.d. | 0.17 |
| H3S10ADPr_1_ | 1301 | 987.7 to 1740 | 0.98 |
| H3S10ADPr_2_ | 762.4 | 690.6 to 842.4 | 0.99 |
| H3S10ADPr_3_ | 64.56 | 54.55 to 76.31 | 0.98 |
| H3S10ADPr_4_ | 65.43 | 53.46 to 79.95 | 0.98 |
| H3S10ADPr_5_ | 42.51 | 33.80 to 53.31 | 0.97 |
| H2B unmodified | n.d. | n.d. | 0.49 |
| H2BS6ADPr_1_ | 1642 | 1411 to 1922 | 0.99 |
| H2BS6ADPr_2_ | 526.5 | 484.5 to 572.4 | 0.99 |
| H2BS6ADPr_3_ | 84.78 | 71.67 to 100.2 | 0.99 |
| H2BS6ADPr_4_ | 96.35 | 77.05 to 120.2 | 0.98 |

**Affinities of Af1521 macrodomain* for ADP-ribosylated histone peptides**

**Affinities of ALC1 macrodomain for ADP-ribosylated histone peptides**

| **Peptide** | **K_d,app_ (nM)** | **95% CI** | **R^2^** |
| --- | --- | --- | --- |
| H3 unmodified | n.d. | n.d. | 0.28 |
| H3S10ADPr_1_ | n.d. | n.d. | 0.36 |
| H3S10ADPr_2_ | n.d. | n.d. | 0.40 |
| H3S10ADPr_3_ | 21.22 | 17.06 to 26.33 | 0.98 |
| H3S10ADPr_4_ | 27.24 | 19.12 to 38.69 | 0.94 |
| H3S10ADPr_5_ | 27.93 | 22.43 to 34.74 | 0.98 |
| H2B unmodified | n.d. | n.d. | 0.29 |
| H2BS6ADPr_1_ | n.d. | n.d. | 0.11 |
| H2BS6ADPr_2_ | 4909 | 2080 to 46956^#^ | 0.91 |
| H2BS6ADPr_3_ | 30.64 | 23.17 to 40.38 | 0.96 |
| H2BS6ADPr_4_ | 36.95 | 28.18 to 48.30 | 0.96 |

* : the Af1521 macrodomain is from the commercially available anti-pan-ADP-ribose binding reagent and has the immunoglobulin Fc fused to it.

n.d. : indicates that the value could not be determined with reliability because of a poor fit to the non-linear regression model being used.

# : indicates that the value is a 90% CI instead of 95%, because the 95% CI could not be assigned a numerical limit.

Abbreviations

CI : confidence interval

R^2^ : r-squared value or the coefficient of determination

app : apparent
