# Supplementary material for: Serine ADP-ribosylation marks nucleosomes for ALC1-dependent chromatin remodeling
Source: eLife. 2021 Dec 7;10:e71502. doi: 10.7554/eLife.71502 (PMC8683085; doi:10.7554/eLife.71502)
Supplement: Supplementary file 6. [file elife-71502-supp6.docx]

| **Nucleosome** | **k (min^-1^)** | **95% CI** | **R^2^** |
| --- | --- | --- | --- |
| unmodified | 0.0001379 | 8.073e-005 to 0.0001952 | 0.62 |
| H3S10ADPr_1_ | 0.0003232 | 5.836e-005 to 0.0005893 | 0.30 |
| H3S10ADPr_3_ | 0.01002 | 0.009478 to 0.01057 | 0.99 |
| H3S10ADPr_4_ | 0.01037 | 0.008533 to 0.01231 | 0.92 |
| H2BS6ADPr_1_ | 0.0001646 | 0.0001004 to 0.0002290 | 0.65 |
| H2BS6ADPr_3_ | 0.01536 | 0.01312 to 0.01775 | 0.95 |
| H2BS6ADPr_4_ | 0.02776 | 0.02207 to 0.03464 | 0.93 |
| H2BS6/H3S10ADPr_3_ | 0.04024 | 0.03546 to 0.04566 | 0.98 |
| H2BS6/H3S10ADPr_4_ | 0.05152 | 0.04040 to 0.06597 | 0.95 |
| unmodified+H3S10ADPr_4_ peptide | 8.323e-005 | 3.061e-005 to 0.0001359 | 0.41 |
| unmodified+H2BS6ADPr_4_ peptide | 1.886e-005 | 0 to 8.611e-005 | 0.02 |
| unmodified+PARP1 (20 nM) | 0.001667 | 0.001166 to 0.002173 | 0.76 |
| unmodified+PARP1 (100 nM) | 0.001970 | 0.001518 to 0.002425 | 0.85 |
| unmodified+PARP1 SerADPr_long_ | 0.005027 | 0.004190 to 0.005880 | 0.92 |
| unmodified+PARP1 SerADPr_short_ | 0.003826 | 0.003256 to 0.004403 | 0.93 |

**Chromatin remodeling rate constants for single-substrate assays with ALC1**

$

$

$

**Chromatin remodeling rate constants for single-substrate assays with CHD4**

| **Nucleosome** | **k (min^-1^)** | **95% CI** | **R^2^** |
| --- | --- | --- | --- |
| unmodified | 0.01010 | 0.008834 to 0.01142 | 0.96 |
| H3S10ADPr_1_ | 0.01049 | 0.008671 to 0.01240 | 0.92 |
| H3S10ADPr_3_ | 0.008974 | 0.007868 to 0.01011 | 0.96 |
| H3S10ADPr_4_ | 0.009624 | 0.008744 to 0.01053 | 0.98 |
| H2BS6ADPr_1_ | 0.007561 | 0.006142 to 0.009034 | 0.90 |
| H2BS6ADPr_3_ | 0.01141 | 0.009590 to 0.01333 | 0.93 |
| H2BS6ADPr_4_ | 0.01092 | 0.008884 to 0.01307 | 0.91 |
| H2BS6/H3S10ADPr_3_ | 0.009619 | 0.008354 to 0.01093 | 0.95 |
| H2BS6/H3S10ADPr_4_ | 0.007857 | 0.006470 to 0.009295 | 0.91 |

$ : indicates that the data did not fit well with the non-linear regression model being used but the values have been plotted in the associated figure (Figure 5E).
