# Supplementary material for: Serine ADP-ribosylation marks nucleosomes for ALC1-dependent chromatin remodeling
Source: eLife. 2021 Dec 7;10:e71502. doi: 10.7554/eLife.71502 (PMC8683085; doi:10.7554/eLife.71502)
Supplement: Supplementary file 7. [file elife-71502-supp7.docx]

**Chromatin remodeling rate constants for multi-substrate assays with ALC1**

**H3 substrate pool**

| **Nucleosome** | **k (min^-1^)** | **95% CI** | **R^2^** |
| --- | --- | --- | --- |
| unmodified (5′_1_) | n.d. | n.d. | -0.11 |
| unmodified (5′_9_) | n.d. | n.d. | 0.15 |
| H3S10ADPr_1_ | n.d. | n.d. | -0.23 |
| H3S10ADPr_3_ | 0.03784 | 0.02821 to 0.05097 | 0.91 |
| H3S10ADPr_4_ | 0.03280 | 0.02194 to 0.04905 | 0.84 |

**H2B substrate pool**

| **Nucleosome** | **k (min^-1^)** | **95% CI** | **R^2^** |
| --- | --- | --- | --- |
| unmodified (5′_1_) | n.d. | n.d. | -0.35 |
| unmodified (5′_9_) | n.d. | n.d. | 0.21 |
| H2BS6ADPr_1_ | n.d. | n.d. | 0.06 |
| H2BS6ADPr_3_ | 0.03637 | 0.02566 to 0.05170 | 0.88 |
| H2BS6ADPr_4_ | 0.06686 | 0.04344 to 0.1042 | 0.90 |

n.d. : indicates that the value could not be determined with reliability because of a poor fit to the non-linear regression model being used.
