# Supplementary material for: Serine ADP-ribosylation marks nucleosomes for ALC1-dependent chromatin remodeling
Source: eLife. 2021 Dec 7;10:e71502. doi: 10.7554/eLife.71502 (PMC8683085; doi:10.7554/eLife.71502)
Supplement: Supplementary file 10. [file elife-71502-supp10.docx]

**DNA nucleotides described in this study**

| **Identifier** | **Sequence (5′ -> 3′)** |
| --- | --- |
| ALC1-KO gRNA1 | GTCGCCTGCATATGTTACAC |
| ALC1-KO gRNA2 | GACCACCTGACTGAGGCTAG |
| PARP stimulating DNA | GCTGGTTCGCGAACCAGC |
| 601 amplification 45-Forward | GGCCGCTCTAGAACTAGTGG |
| 601 amplification 8-Reverse | CTCTGATGCTGGAGAATCC |
| 601 amplification 5′ 1 | GCCATCACGCCACAGTTTCGATCCGATATCGCTGTTCACC |
| 601 amplification 5′ 2 | CGCTGACGCACTCAAATGCGATCCGATATCGCTGTTCACC |
| 601 amplification 5′ 3 | CGGCTAAGAGTAGGTTGGGGATCCGATATCGCTGTTCACC |
| 601 amplification 5′ 4 | CTTACTAAGGCCATCGCGGGATCCGATATCGCTGTTCACC |
| 601 amplification 5′ 5 | CACGATTCAACTACGCCGCGATCCGATATCGCTGTTCACC |
| 601 amplification 5′ 6 | CGTGACGACGTTCCTGCTAGATCCGATATCGCTGTTCACC |
| 601 amplification 5′ 7 | CGCTGCAGACACTATACCGGATCCGATATCGCTGTTCACC |
| 601 amplification 5′ 8 | GCAGCGACATCCACTTGAGGATCCGATATCGCTGTTCACC |
| 601 amplification 5′ 9 | CAGCAGTTCGTCTCTGCTGGATCCGATATCGCTGTTCACC |
| 601 amplification 5′ 10 | CAAGACTCGCCTTACGGCTGATCCGATATCGCTGTTCACC |
| qPCR 5′ 1 | GCCATCACGCCACAGTTTC |
| qPCR 5′ 2 | CGCTGACGCACTCAAATGC |
| qPCR 5′ 3 | CGGCTAAGAGTAGGTTGGG |
| qPCR 5′ 4 | CTTACTAAGGCCATCGCGG |
| qPCR 5′ 5 | CACGATTCAACTACGCCGC |
| qPCR 5′ 6 | CGTGACGACGTTCCTGCTA |
| qPCR 5′ 7 | CGCTGCAGACACTATACCG |
| qPCR 5′ 8 | GCAGCGACATCCACTTGAG |
| qPCR 5′ 9 | CAGCAGTTCGTCTCTGCTG |
| qPCR 5′ 10 | CAAGACTCGCCTTACGGCT |
| Universal qPCR-Reverse | CTCTGATGCTGGAGAATCCCG |
