# Supplementary material for: Serine ADP-ribosylation marks nucleosomes for ALC1-dependent chromatin remodeling
Source: eLife. 2021 Dec 7;10:e71502. doi: 10.7554/eLife.71502 (PMC8683085; doi:10.7554/eLife.71502)
Supplement: Supplementary file 11. [file elife-71502-supp11.docx]

**Antibodies used in this study**

| **Antibody** | **Source** | **Identifier** | **Dilution** |
| --- | --- | --- | --- |
| IRDye 800CW Goat anti-Rabbit IgG (H+L) | Thermo-Fisher Scientific | NC9401842 | 1:5000 |
| IRDye 680RD Goat anti-Mouse IgG (H+L) | Thermo-Fisher Scientific | NC0252290 | 1:5000 |
| Anti-pan-ADP-ribose binding reagent | Millipore Sigma | MABE1016 | 1:1000 |
| Histone H3 (D1H2) XP® Rabbit mAb #4499 | Cell Signaling Technologies | 4499S | 1:8000 |
| Histone H2B (D2H6) Rabbit mAb #12364 | Cell Signaling Technologies | 12364S | 1:2000 |
| Histone H2A (L88A6) Mouse mAb #3636 | Cell Signaling Technologies | 3636A | 1:600 |
| PARP (46D11) Rabbit mAb #9532 | Cell Signaling Technologies | 9532S | 1:1000 |
| CHD1L (E1I8C) Rabbit mAb #13460 | Cell Signaling Technologies | 13460S | 1:1000 |
| CHD1 (D8C2) Rabbit mAb #4351 | Cell Signaling Technologies | 4351S | 1:1000 |
| SNF2H (D4W6N) Rabbit mAb #38410 | Cell Signaling Technologies | 38410S | 1:1000 |
| CHD4 (D8B12) Rabbit mAb #11912 | Cell Signaling Technologies | 11912S | 1:1000 |
| Brg1 (P680) Rabbit Antibody #3514 | Cell Signaling Technologies | 3514S | 1:1000 |
| Anti-histone H3 antibody mouse ab10799 | Abcam | 10799 | 1:1000 |
